# TORCphysics: a physical model of DNA-topology-controlled gene expression

**DOI:** 10.1093/nar/gkag126

**Published:** 2026-02-18

**Authors:** Victor Velasco-Berrelleza, Penn Faulkner Rainford, Aalap Mogre, Craig J Benham, Charles J Dorman, Carsten Kröger, Susan Stepney, Sarah A Harris

**Affiliations:** School of Mathematical and Physical Sciences, University of Sheffield, Hounsfield Road, Sheffield, S3 7RH, United Kingdom; Department of Computer Science, University of York, York, YO10 5DD, United Kingdom; Department of Microbiology, School of Genetics and Microbiology, Moyne Institute of Preventive Medicine, Trinity College Dublin, College Green, Dublin, D02 PN40, Ireland; Department of Mathematics, University of California, Davis, One Shields Avenue, Davis, CA 95616, United States; Department of Microbiology, School of Genetics and Microbiology, Moyne Institute of Preventive Medicine, Trinity College Dublin, College Green, Dublin, D02 PN40, Ireland; Department of Microbiology, School of Genetics and Microbiology, Moyne Institute of Preventive Medicine, Trinity College Dublin, College Green, Dublin, D02 PN40, Ireland; Department of Computer Science, University of York, York, YO10 5DD, United Kingdom; School of Mathematical and Physical Sciences, University of Sheffield, Hounsfield Road, Sheffield, S3 7RH, United Kingdom

## Abstract

DNA superhelicity and transcription are intimately related because changes to DNA topology can influence gene expression and vice versa. Information is transferred through the modulation of local DNA torsional stress, where the expression of one gene may influence the superhelical level of neighbouring genes, either promoting or repressing their expression. In this work, we introduce a one-dimensional physical model that simulates supercoiling-mediated regulation. This TORCphysics model takes as input a genome architecture represented either by a plasmid or chromosomal DNA sequence with ends constrained under specific biological conditions and computes the molecule’s output. Our findings demonstrate that the expression profiles of genes are directly influenced by the gene circuit design, including gene location, the positions of topological barriers, promoter sequences, and topoisomerase activity. The novelty that TORCphysics offers is versatility, where users can define distinct activity models for different types of proteins and protein-binding sites. The aim of this research is to establish a flexible framework for developing physical simulations of gene circuits to deepen our comprehension of the intricate mechanisms involved in gene regulation.

## Graphical Abstract

**Graphical Abstract:**
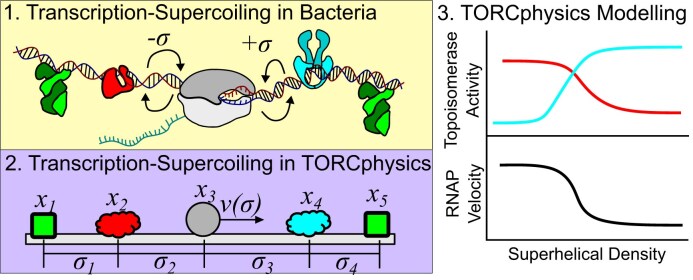

## Introduction

The level of over- or underwinding of the DNA double helix, known as supercoiling, is a fundamental topological property of DNA. In bacteria, DNA is typically maintained in a negatively supercoiled (underwound) state. The DNA is therefore under torsional stress, as turns have been removed from the double helix [1]. This underwound state is regulated by a delicate balance between the activities of topoisomerases, enzymes that modulate superhelical stress. In bacteria, topoisomerase I (type IA) removes supercoils via single-strand breaks, while gyrase (type IIA) introduces negative supercoils by cutting and rejoining both strands in an ATP-dependent manner [2–5]. In the absence of ATP, gyrase is capable of relaxing negatively supercoiled DNA [6]. A recent FRET-based kinetics study on fluorescent labelled plasmids shows that both enzymes follow classical Michaelis-Menten kinetics in *Escherichia coli* [7].

DNA supercoiling is intimately related to transcription. It can control promoter activity and influence various stages of transcription [4, 8, 9]. During closed transcription complex formation, DNA supercoiling influences the binding rate of RNA polymerases (RNAPs) by altering the geometric orientation of the $-10$ and $-35$ promoter elements that are separated by the promoter spacer length [10]. In open-complex formation, negative supercoiling may reduce the free energy required for strand separation at the promoter, thereby facilitating transcription bubble formation [11–13]. During elongation, RNAP acts as a mobile topological barrier, generating positive supercoils downstream and negative supercoils upstream, which can propagate and influence the expression of distal genes as well as the gene being transcribed [14–18]. Both positive and negative supercoils can stall RNAPs [17, 19], while topoisomerases help alleviate torsional stress, thereby enabling transcription to resume [14, 15, 18, 20–22]. Recent evidence suggests that topoisomerase I physically interacts with transcribing RNAPs upstream, and this interaction may play a crucial role in transcript elongation [23–27].

Quantitatively predicting how DNA supercoiling modulates gene expression introduces further complexity into models that rely solely on transcription factors to promote or repress gene expression [28]. Several models have emerged to investigate the coupling between supercoiling and transcription [14, 18, 29–42]. These models vary in focus: some emphasize transcription initiation [14, 31, 32, 38], others explore the collective motion of RNAPs during elongation and its effects on transcription bursting [34–36, 38], and others investigate how transcription is influenced by supercoiling diffusion [33, 34, 40]. While most of these works investigate transcription-supercoiling dynamics in two-gene architectures with genes arranged in different orientations [14, 35, 41], a recent study focused on single-gene architectures, showing that even in this minimal system, DNA supercoiling plays a crucial role in modulating gene expression [18].

In this work, we introduce TORCphysics, a novel, versatile, and fast one-dimensional physical model that accounts for the interactions between DNA and proteins such as RNAP and topoisomerases through DNA supercoiling, with a particular focus on transcription. The novelty that TORCphysics offers lies in its versatility to incorporate and combine existing models while also allowing users to define new mechanisms for biomacromolecules of interest. The framework can simulate both circular DNA sequences (plasmids) and linear chromosomal segments. Taking inspiration from previous works, here we define new models that simulate the stochastic binding of topoisomerases to supercoiled DNA, the interaction between topoisomerase I and transcribing RNAPs, a twist injection ratio introduced by elongating RNAPs, and the impact of DNA supercoiling throughout different stages of transcription initiation and elongation. We assume all changes to DNA superhelicity introduced by bound proteins are expressed exclusively as changes in twist: we do not account for changes in writhe or any resulting structures such as plectonemes. Even with these assumptions, our results provide mechanistic insight into gene regulation through DNA supercoiling. By revisiting previous experimental studies, we calibrate our models and propose potential mechanisms that may explain observed experimental phenomena. Our results demonstrate that DNA-binding proteins both contribute and respond to local DNA supercoiling, revealing complex mechanisms even in simple genetic architectures such as single-gene systems. TORCphysics provides a flexible platform for simulating genetic circuits, enabling users to adjust the complexity of molecular interactions to investigate, compare, and explain experimental findings.

## Methods–CoSMoS overview

To develop the TORCphysics system, we follow the CoSMoS (Complex Systems Modelling and Simulation) approach, as described in detail in [43]. Here, we provide a brief overview of the approach.

**Figure 1.**
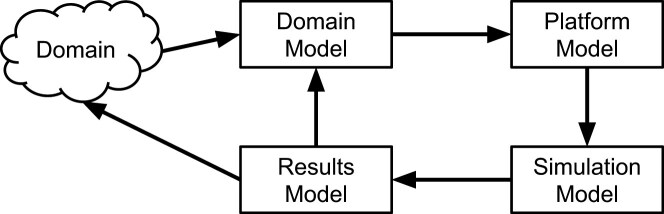
The modelling components of a CoSMoS simulation development. The CoSMoS approach formalizes the construction of models and simulations of complex systems and here is applied to transcription regulation through DNA supercoiling in the TORCphysics scheme.

CoSMoS involves a variety of components: domain, domain model, platform model, simulation platform, and results model (Fig. 1). Each of these components plays a particular role the design, implementation, and use of a simulation.

### Domain

This is (a view of) the particular real-world system being simulated. The TORCphysics domain is DNA supercoiling and transcription in bacteria. It is described in the ‘Introduction’ section.

### Domain model

This is a scientific model of the relevant parts of the domain. It can incorporate understanding gained from domain experiments and observations, and hypotheses of the particular underlying mechanisms of interest. Crucially, the domain model is expressed in terms of relevance to the domain scientists, is validated by them as an accurate representation of the domain, and does not include simulation implementation details.

The TORCphysics domain model is a model of the physical processes involved in supercoiling and gene expression, given in terms of ordinary differential equations. It is detailed in the ‘Domain model’ section.

### Platform model

This is a software engineering model, or requirements specification, of the simulation software to be developed. It maps the domain model into computational terms and includes necessary implementation detail. The mapping may make assumptions, approximations, and other changes in order to make a computational simulation feasible. For example, a continuous time domain model will need to be converted to a discrete time platform model.

The platform model captures the low-level (possibly hypothesized) domain model mechanisms, explicitly omitting the high-level (emergent) properties observed in domain experiments. This ensures that those emergent properties are not ‘hardcoded’ into the simulation, thereby allowing them to emerge (or not) from the hypothesized mechanisms. For example, the domain model might include gene expression levels hypothesized to be caused by particular supercoiling mechanisms. The platform model would include the mechanisms but not hardcode the expression levels: these would emerge as consequences of the mechanisms during simulation experiment runs. If they match the domain values, this provides evidence to support the mechanisms; if they do not, other mechanisms may need to be sought.

The TORCphysics platform model is described in the ‘Platform model’ section.

### Simulation platform

This is the software implementation of the platform model, calibrated against experimental data as required. The calibrated platform is used to perform simulation experiments. Note the logical distance of the simulation code from the domain: it is not a direct ‘coding up’ of domain concepts. A CoSMoS simulator is carefully engineered in a manner explicitly designed to ensure that any emergent properties in the results have not been accidentally hardcoded in; hence, the hypothesized mechanisms can be rigorously evaluated.

The TORCphysics simulation platform code is available from https://github.com/Victor-93/TORCphysics/tree/TORCphysics_paper. The TORCphysics simulation platform’s calibration is detailed in the ‘Simulation platform’ Methods section. More information about the TORCphysics repository and code availability is provided in Supplementary Methods, Section 1.

### Results model

This descriptive model captures the outputs from the simulation platform. It captures the results from simulation experiments in a form that can be directly compared to domain (real-world) experimental results and, potentially, to the domain itself, thereby testing whether the hypothesized low-level mechanisms can result in the observed high-level properties.

The TORCphysics experiments and results are discussed in the ‘Results and discussion’ section, with different versions of different hypothesized mechanisms matching the domain observations to different degrees. Finally, the sensitivity analysis of TORCphysics is described and discussed in the Supplementary Methods, Section 2. This analysis shows that variations translate into corresponding changes in its physical description within the model in the expected way, and consequently that our parameterization procedure remains stable so long as the parameters chosen are physically reasonable.

## Methods–TORCphysics

TORCphysics provides a physical model of supercoiling-mediated regulation of gene expression in gene circuits that are sufficiently small that supercoiling propagates effectively instantaneously. Our model accounts for the stochastic transcription initiation for both supercoiling-sensitive and non-sensitive genes (promoters), while also considering the influence of stochastic binding of proteins such as topoisomerases (topoisomerase I and gyrase) and RNAPs. The model quantifies the mechanical impact of these proteins on DNA, specifically addressing supercoiling as changes in the twisting of the double helix; writhe and resulting forms such as plectonemes are not accounted for in the current model. The local structural variations in twist propagate along the DNA, allowing interactions among various bound enzymes and binding sites.

TORCphysics aims to provide the number of RNA transcripts made per second from each gene being considered. It also provides the dynamics of the gene circuit in terms of the interactions between proteins and DNA, transcription events, and changes in the superhelical density of the DNA as a function of time. Protein binding to DNA depends on both sequence and local superhelical density, which is parameterized as a pre-processing step. Currently TORCphysics does not consider translation or messenger RNA (mRNA) degradation and assumes that there is sufficient ATP for DNA gyrase always to be active and introduce negative supercoils, which corresponds to an environment where the bacteria live in rich media.

The novelty of TORCphysics lies in its ability to incorporate and combine existing models while providing the versatility for users to define new mechanisms. In the present study, we integrate the models described in the ‘Domain model’ section. Among the new components introduced are the stochastic binding of topoisomerases modulated by sigmoidal functions; their linear and mechanical effects on supercoiled DNA in terms of the twist angle; the twist generated by transcribing RNAPs; the three-step transcription model describing promoter kinetics; and the RNAP tracking by topoisomerase I model. Although many of these components are inspired by previous work, to our knowledge, no existing framework has combined them in this manner. We therefore believe that TORCphysics represents a valuable platform for the community to use and build upon.

To support the incorporation of custom mechanisms in the future, we consider three different levels in which TORCphysics can be modified. Level 1 modifications will simply add or make a small change to an already existing TORCphysics function. A level 2 modification would add a mechanism requiring slight changes to the TORCphysics code, e.g., DNA looping and R-loop formation. Lastly, level 3 requires substantial modifications to the software and potentially the workflow. Examples include reactions in the environment, such as ATP/ADP ratio, translation, and mRNA degradation. Supplementary Methods, Section 3 shows how these modifications could be incorporated into TORCphysics in more in detail, and also provides examples of how users can implement them.

### Domain model

#### Calculating the superhelical density within TORCphysics

DNA topology is described through the linking difference $\Delta \mathrm{Lk}= \mathrm{Lk} - \mathrm{Lk}_0$, where $\mathrm{Lk}=\mathrm{Tw}+\mathrm{Wr}$ is the linking number defined as the total twist (Tw) and writhe (Wr) of the topological domain. $\mathrm{Lk}_0$ refers to the relaxed linking number. The superhelical density $\sigma$ can be expressed in terms of the linking difference as:


(1) \begin{eqnarray*}
\sigma = \frac{\Delta \mathrm{Lk}}{\mathrm{Lk}_0}.
\end{eqnarray*}

Assuming that changes in topology are exclusively from twist ($\mathrm{Wr}=0$), and that the relaxed linking number corresponds to the total twist of a B-DNA structure of length $L$ (in base pairs), such that $\mathrm{Lk}_0=w_0L$, where $w_0$ (in rad/bp) is the relaxed twist density of B-DNA, the superhelical density can be reformulated in terms of the additional twist $\phi$ (in radians) applied to, or removed from, the relaxed structure


(2) \begin{eqnarray*}
\sigma = \frac{\phi }{w_0L}.
\end{eqnarray*}

For a topological domain defined by a DNA region constrained at its two ends $x_{i}$ and $x_{i+1}$, the superhelicity within the domain will be isolated from the outside regions. Twisting this region of the DNA by a twist angle of $\phi _i$ from its relaxed state, the resulting local superhelical density $\sigma _i$ is given by


(3) \begin{eqnarray*}
\sigma _i = \frac{\phi _i}{\omega _0 L_{i} },
\end{eqnarray*}

where $L_i=|x_{i+1}-x_{i}|$ is the length of the topological domain. This assumes that there is no contribution from writhe (i.e. no plectoneme formation) and neglects any non B-DNA motifs such as cruciforms (including stem regions) or single-stranded regions [44].

Although TORCphysics does not explicitly account for contributions to superhelicity beyond twist, it can approximate the corresponding change in writhe ($\Delta \mathrm{Wr}$) and twist ($\Delta \mathrm{Tw}$) of supercoiled structures by partitioning the linking difference $\Delta \mathrm{Lk} = \Delta \mathrm{Tw} + \Delta \mathrm{Wr}$ and assuming an equilibrium twist-to-writhe ratio of $1:3$ [1, 45]. Thus, $\Delta \mathrm{Tw} = 0.25 \times \Delta \mathrm{Lk}$ and $\Delta \mathrm{Wr} = 0.75 \times \Delta \mathrm{Lk}$. While writhe is not directly modelled, this approximation is suitable for comparing simulations with experimental data where writhe is quantified [45].

#### Topoisomerase activity on DNA

**Figure 2.**
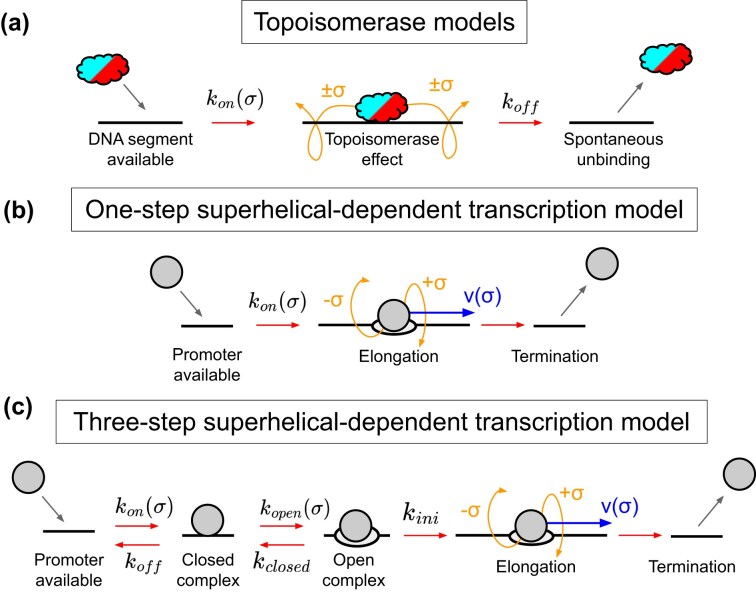
Representation of physical models of enzyme dynamics. (**a**) Models of topoisomerse I (red) and gyrase (cyan) activities in superhelical DNA, where they can bind, modify and unbind the DNA. (**b**) One-step model to describe superhelical-dependant transcription, where elongation starts immediately after the RNAP (grey) binds the DNA. (**c**) Three-step model for describing superhelical-dependant transcrition with several initiation stages before irreversible elongation.

Figure 2a shows the model we use for describing topoisomerase activity, which is characterized by binding, followed by changes in DNA twist, and finally unbinding from the DNA. We model the binding of topoisomerase I and gyrase on DNA through rate equations (4) and (5). Similar to previous studies [14, 40], we employ sigmoidal functions that modulate the binding rates of topoisomerase I and gyrase as a function of the local superhelical density


(4) \begin{eqnarray*}
k_{\mathrm{topoI}}(\sigma ) = \frac{k_{\mathrm{on,topoI}} E_{\mathrm{topoI}}}{1+\exp {((\sigma -\sigma _{t,\mathrm{topoI}})/\sigma _{w,\mathrm{topoI}})}},
\end{eqnarray*}

(5) \begin{eqnarray*}
k_{\mathrm{gyrase}}(\sigma ) = \frac{k_{\mathrm{on,gyrase}} E_{\mathrm{gyrase}}}{1+\exp {(-(\sigma -\sigma _{t,\mathrm{gyrase}})/\sigma _{w,\mathrm{gyrase}})}},
\end{eqnarray*}

where $k_{\mathrm{topoI}}$ and $k_{\mathrm{gyrase}}$ denote the supercoiled-dependent binding rates of topoisomerase I and gyrase respectively (in $s^{-1}$), $k_{\mathrm{on}}$ corresponds to the basal binding rates (in $nM^{-1}s^{-1}$), $E$ is the enzyme concentration (in $nM$), and $\sigma _t$ and $\sigma _w$ refer to the threshold and width of the sigmoidal function respectively (see Table 1). Here we assume the binding of topoisomerase I and gyrase to DNA is determined only by the DNA superhelical density $\sigma$, and is sequence independent. Depending on the parameterization of the sigmoidal functions, both topoisomerase I and gyrase can bind to negatively or positively supercoiled regions of DNA.

**Table 1.** TORCphysics parameters

Parameter	Value	Varies	Source	Definition
** $\omega _0$ (rad/bp)**	$2\pi /10.5$	No	Intrinsic to B-DNA	Relaxed B-DNA twist density
** $\Delta t$ (s)**	1	No	Simulation	Simulation time-step
** $v_0$ (bp/s)**	30	No	Jie Ma *et al*. [19]	RNAP transcribing velocity
** $\gamma$ **	$0.157 \pm 0.121$	No	RNAPTracking section	RNAP twist injection ratio
	0.01		Chatterjee *et al*. [37]	
** $\kappa$ pN$^{-1}$**	0.5	No	Sevier *et al*. [39]	Torque scaling factor in RNAP velocity
** $\tau _0$ pN**	12	No	Sevier *et al*. [39]	RNAP stalling torque
** $l_{\mathrm{topoI}}$ (bp)**	20	No	Zhang *et al*. [49]	Topoisomerase I binding site size
** $l_{\mathrm{gyrase}}$ (bp)**	30	No	Vanden Broeck *et al*. [50]	Gyrase binding site size
** $l_{\mathrm{RNAP}}$ (bp)**	30	No	Kang *et al*. [56]	RNAP binding site size
** $E_{\mathrm{topoI}}$ (nM)**	17.0	Yes	Wang *et al*. [7]	Topoisomerase I concentration
** $E_{\mathrm{gyrase}}$ (nM)**	44.6	Yes	Wang *et al*. [7]	Gyrase concentration
** $k_{\mathrm{on,topoI}}$ (nM$^{-1}$s$^{-1}$)**	$(3.54 \pm 0.042) \times 10^{-4}$	No	Topoisomerase section	Topoisomerase I binding rate
	$1.06 \times 10^{-4}$		Geng *et al*. [40]	
** $k_{\mathrm{on,gyrase}}$ (nM$^{-1}$s$^{-1}$)**	$(2.17 \pm 0.0145) \times 10^{-4}$	No	Topoisomerase section	Gyrase binding rate
	$0.40 \times 10^{-4}$		Geng *et al*. [40]	
** $k_{\mathrm{off,topoI}}$ (s$^{-1}$)**	$0.207 \pm 0.110$	No	Topoisomerase section	Topoisomerase I unbinding rate
	0.500		Geng *et al*. [40]	
** $k_{\mathrm{off,gyrase}}$ (s$^{-1}$)**	$0.186 \pm 0.076$	No	Topoisomerase section	Gyrase unbinding rate
	0.500		Geng *et al*. [40]	
** $k_{\phi ,\mathrm{topoI}}$ **	$15.11 \pm 3.04$	No	Topoisomerase section	Topoisomerase I twist injection rate
** $k_{\phi ,\mathrm{gyrase}}$ **	$13.94 \pm 3.42$	No	Topoisomerase section	Gyrase twist injection rate
** $\sigma _{t,\mathrm{topoI}}$ **	$0.012 \pm 0.010$	No	Topoisomerase section	Sigmoid for topoisomerase I binding (threshold)
	$-0.040$		El Houdaigui *et al*. [14]	
** $\sigma _{t,\mathrm{gyrase}}$ **	$-0.059 \pm 0.001$	No	Topoisomerase section	
	0.0145		Geng *et al*. [40]	Sigmoid for gyrase binding (threshold)
	0.010		El Houdaigui *et al*. [14]	
** $\sigma _{w,\mathrm{topoI}}$ **	$(4.25 \pm 1.64) \times 10^{-3}$	No	Topoisomerase section	Sigmoid for topoisomerase I binding (width)
	$12.0 \times 10^{-3}$		El Houdaigui *et al*. [14]	
** $\sigma _{w,\mathrm{gyrase}}$ **	$(12.3 \pm 0.2) \times 10^{-3}$	No	Topoisomerase section	Sigmoid for gyrase binding (width)
	$12.2 \times 10^{-3}$		Geng *et al*. [40]	
	$25.0 \times 10^{-3}$		El Houdaigui *et al*. [14]	
** $\sigma _{0,\mathrm{gyrase}}$ **	$-0.146 \pm 0.0034$	No	Topoisomerase section	Gyrase maximum superhelicity
** $\alpha _{E,\mathrm{topoI}}$ **	$18.61 \pm 6.78$	No	RNAPTracking section	Topoisomerase I binding enhancer factor
** $d_{\mathrm{topoI}}$ (bp)**	$448 \pm 40$	No	RNAPTracking section	Enhancer-RNAP effective distance
** $k_{\text{on, promoter}}$ (s$^{-1}$)**	[0.1, 0.33]	Yes	Gene architecture section	Closed-complex formation rate (forward)
** $k_{\text{off, promoter}}$ (s$^{-1}$)**	[0.2, 0.4]	Yes	Gene architecture section	RNAP unbinding rate
** $k_{\text{open, promoter}}$ (s$^{-1}$)**	[0.01, 0.42]	Yes	Gene architecture section	Open-complex formation rate
** $k_{\text{closed, promoter}}$ (s$^{-1}$)**	[0.08, 0.45]	Yes	Gene architecture section	Closed-complex formation rate (reversed)
** $k_{\text{ini, promoter}}$ (s$^{-1}$)**	[0.05, 0.42]	Yes	Gene architecture section	Transcription initiation rate
** $\sigma _{e,\text{on, promoter}}$ **	$[-0.060,-0.045]$	Yes	Gene architecture section	Gaussian for promoter binding (threshold)
** $\epsilon _{\text{e, promoter}}$ **	[0.005, 0.014]	Yes	Gene architecture section	Gaussian for promoter binding (width)
** $\sigma _{m,\text{on, promoter}}$ **	$[-0.067, -0.075]$	Yes	Gene architecture section	Sigmoid for promoter melting (threshold)
	$-0.042$		El Houdaigui *et al*. [14]	
** $\epsilon _{\text{m, promoter}}$ **	[0.0089, 0.0093]	Yes	Gene architecture section	Sigmoid for promoter melting (width)
	0.005		El Houdaigui *et al*. [14]	

Binding rates *k*_on_ from Geng *et al*. [40] were adjusted to match simulation conditions.

Once bound, both enzymes can alter the local twist and therefore the superhelical density in a topological domain. To this end, we define the models for the change in twist which are linear with respect to the superhelical density:


(6) \begin{eqnarray*}
\frac{\mathrm{ d} \phi _{\mathrm{topoI}}}{\mathrm{ d}t} = - k_{\phi ,\mathrm{topoI}}\omega _0 \sigma,
\end{eqnarray*}

(7) \begin{eqnarray*}
\frac{\mathrm{ d} \phi _{\mathrm{gyrase}}}{\mathrm{ d}t} = k_{\phi ,\mathrm{gyrase}}\omega _0 ( \sigma _0 - \sigma ),
\end{eqnarray*}

where $d\phi _{\mathrm{topoI}}$ and $d\phi _{\mathrm{gyrase}}$ denote the twist angle induced by topo I and gyrase activity respectively, $k_{\phi }$ is the rate constant that defines the number of base-pairs by which the DNA is either under or over-twisted per second (bp/s), $\omega _0$ is the twist density (rad/bp) as in equation (3), and $\sigma _0$ is the superhelical density threshold at which gyrase can have an effect on the DNA (see Table 1). Past this threshold, gyrase is not able to introduce additional negative supercoils into the DNA, and it holds the superhelical density at $\sigma _0$ while it remains bound. The angular twist rate can also be expressed as $k^{\prime }_\phi = k_\phi \omega _0$ (rad/s); however, we use $k_\phi$ as this offers an intuitive interpretation in terms of the number of base pairs completely over/under-twisted per second. Specifically, $k_\phi = 1$ (bp/s) means that one base pair is fully twisted every second, which corresponds to ~0.6 radians per second. To our knowledge, this assumption of linear dependency on superhelicity is specific to TORCphysics.

Lastly, we consider the unbinding of topoisomerases to be a spontaneous process, with each enzyme having an associated unbinding rate $k_{\mathrm{off}}$. Note that topoisomerases can remain bound to DNA for multiple cycles, performing several rounds of enzymatic activity before dissociating.

#### One-step superhelical-dependent transcription model

Similar to previous studies [14, 31, 40], the one-step model treats superhelical-sensitive transcription initiation as an instant binding/initiation, where RNAPs are recruited to available promoters and transcription immediately starts (see Fig. 2b). We model this behaviour by employing a sigmoid function that represents the energy required to melt the promoter, represented by a free energy function $U_\mathrm{melt}(\sigma )$


(8) \begin{eqnarray*}
k_{\mathrm{on}}(\sigma ) = k_{\mathrm{on}} \exp \left(- U^{\prime }_{\mathrm{melt}}(\sigma ) \right),
\end{eqnarray*}

with


(9) \begin{eqnarray*}
U^{\prime }_{\mathrm{melt}}(\sigma ) = \frac{\mu }{1 + \exp \left(-{(\sigma - \sigma _m)}/{\epsilon _m}\right)},
\end{eqnarray*}

where $ k_{\mathrm{on}}$ denotes the basal rate of RNAP binding, $ \sigma _m$ denotes the threshold for melting, and $ \epsilon _m$ is the sigmoid width. In practice, rather than directly using the free energy function $U_\mathrm{melt}(\sigma )$ (in kcal/mol), we use a dimensionless rescaled version, $U^{\prime }_\mathrm{melt}(\sigma )$, that captures the promoter response to melting via parameters $\sigma _m$ and $\epsilon _m$. The dimensionless parameter $\mu \approx 2.3$ is chosen so that in the inhibitory regime ($\sigma \gg \sigma _m$) the rate $k_\mathrm{on}(\sigma )$ is reduced to $10\%$ of its value, i.e., $k_{\mathrm{on}}(\sigma \rightarrow \infty ) = k_{\mathrm{on}} \exp (-\mu ) = 0.1 \, k_{\mathrm{on}}$ (see Supplementary Methods, Section 4). The free energy $U_\mathrm{melt}$ (and its scaled version $U^{\prime }_\mathrm{melt}$) is sequence-dependent and can be parametrized using the SIST algorithm [13] to calculate strand-separation profiles at various superhelical densities. Because of this, binding rate modulation is highly sensitive to GC content, with GC-rich sequences melting more slowly than AT-rich regions, which are more prone to strand separation. The procedure for parameterizing $U_\mathrm{melt}(\sigma )$ with SIST is detailed in Supplementary Methods, Section 4.

Once RNAPs bind their promoters, they automatically transition to the elongation stage, where the RNAP advances along the gene, inducing negative supercoils upstream and positive downstream, until RNAP reaches the termination site. Within this model (equation 10) the build up of supercoils may stall the enzyme. Once the supercoils are alleviated, the RNAP can proceed until it reaches the termination site. These dynamics are captured by the following equations:


(10) \begin{eqnarray*}
v = \frac{\mathrm{ d}x}{\mathrm{ d}t} =\frac{v_0}{1+\exp \left( \kappa (\tau (\sigma ) - \tau _0) \right)},
\end{eqnarray*}

(11) \begin{eqnarray*}
\frac{\mathrm{ d} \phi }{\mathrm{ d}t} = \pm v \gamma \omega _0.
\end{eqnarray*}

The movement speed of RNAPs is based on the mechanical framework proposed in [39]. The parameter $ \tau _0 = 12\, \text{pN nm}$ corresponds to the stalling torque, and the scaling factor $ \kappa = 0.5\, \mathrm{pN}^{-1} \mathrm{nm}^{-1}$. The torque $ \tau (\sigma )$ (see Fig. 2) is modelled using Marko’s elastic model of supercoiled DNA [46] (see Supplementary Methods Section 5 and Supplementary Table S1 for details). We use an RNAP transcription velocity of $v_0 = 30$ bp/s, and if the torque exceeds the stalling threshold $\tau _0$, the RNAP stalls. While RNAP velocity is likely variable under realistic biological conditions, we assume a constant velocity for the purposes of this work. We compute the magnitude of the torque exerted on the RNAP as $\tau = \left| \tau _\mathrm{downstream} - \tau _\mathrm{upstream} \right|$, with the torque function and the resulting RNAP velocity shown in Supplementary Fig. S1. The parameter $ \gamma$ is dimensionless and quantifies the twist injected by the RNAP per base pair transcribed. The value of $ \gamma$ ranges from 0 to 1, where $ \gamma = 0$ corresponds to no twist being imparted on the DNA by the RNAP, implying that the RNAP smoothly rotates around the DNA during elongation, and $ \gamma = 1$ represents a scenario where each base pair transcribed results in complete under- or over-twisting of the DNA. The value of $\gamma$ depends on several factors, such as the viscosity of the surrounding medium, and the size of the transcription complex, which may even include the translational machinery. Here we assume that $\gamma$ is a constant; however, this parameter may vary with transcript length.

Finally, RNAPs advance until they reach a terminator site, where they instantly unbind the DNA.

#### Three-step superhelical-dependent transcription model

To better capture transcription and its impact on DNA, we propose a three-step model where RNAPs transition through a series of reversible stages of transcription until the elongation phase, as shown in Fig. 2c. Upon binding to a promoter, the RNAP forms a closed complex, the probability of which is modulated by the local superhelical state through an elastic function $ G_{\mathrm{elastic}}(\sigma )$, related to the optimal orientation for the promoter [10]. This is followed by a transition to an open complex, which is governed by the energy required to melt the promoter, represented by the rescaled free energy function $ U^{\prime }_{\mathrm{melt}}(\sigma )$. These two key processes are described by


(12) \begin{eqnarray*}
k_{\mathrm{on}}(\sigma ) = k_{\mathrm{on}} \exp \left(-G_{\mathrm{elastic}}(\sigma )\right),
\end{eqnarray*}

(13) \begin{eqnarray*}
k_{\mathrm{open}}(\sigma ) = k_{\mathrm{open}} \exp \left(-U^{\prime }_{\mathrm{melt}}(\sigma )\right),
\end{eqnarray*}

where


(14) \begin{eqnarray*}
G_{\mathrm{elastic}}(\sigma ) = \frac{(\sigma - \sigma _e)^2}{2 \epsilon _e^2}.
\end{eqnarray*}

$U^{\prime }_\mathrm{melt}$ corresponds to equation (9), $ k_{\mathrm{on}}$ and $ k_{\mathrm{open}}$ denote the basal rates of RNAP binding and promoter melting respectively, $ \sigma _e$ and $ \sigma _m$ denote the thresholds for binding and melting, $ \epsilon _e$ and $ \epsilon _m$ are the widths of the corresponding distributions. The open complex formation rate $k_\mathrm{open}$ is equivalent to equation (8) in the one-step superhelical-dependent transcription model. The elastic function $ G_{\mathrm{elastic}}(\sigma )$, which modulates the binding rate $ k_{\mathrm{on}}(\sigma )$, is equivalent to the spacer length model proposed by Forquet *et al*. [10]. While that work employed a specific parameterization of $ \sigma _e$ and $ \epsilon _e$, here we use general model for the spacer modulation that can be adapted to an arbitrary promoter sequences and spacer length.

Here, both $U_\mathrm{melt}$ and $G_\mathrm{elastic}$ are sequence-dependent functions that must be pre-computed before TORCphysics simulations because of their computational cost. $U_\mathrm{melt}$ is obtained by running the SIST algorithm multiple times at different superhelical densities, with run time increasing considerably depending on the sequence context. The sequence-dependent characteristic of $G_\mathrm{elastic}$ are discussed in Supplementary Methods, Section 6.

From the open-complex formation, the RNAP then transitions to the irreversible elongation stage according rate $k_\mathrm{ini}$, where the RNAP advances along the gene, inducing supercoils. From there on, the model implements the same set of equations for the elongation phase as in the one-step superhelical-dependant transcription model (see equations 10 and 11). Ranges for the promoter-dependent rates and Gaussian parameters (e.g. widths $\epsilon$ and thresholds $\sigma$) from our calibrations are shown in Table 1.

In this three-step model, each step is reversible prior to elongation initiation (see Fig. 2c). The reverse transitions are governed by their respective rates: $k_{\mathrm{closed}}$ for the return from the open- to the closed-complex, and $k_\mathrm{off}$ for unbinding from the closed-complex. Although in reality $k_\mathrm{off}$ and $k_{\mathrm{closed}}$ may depend on the superhelical density, in this model we treat them as constants for simplicity and to minimize the number of physical parameters required to model transcription initiation of single promoters.

Finally, elongating RNAPs unbind instantaneously upon reaching a terminator site.

#### Modelling the tracking of RNAP by topoisomerase I

To mimic the behaviour observed in the ChIP-Seq data reported by Sutormin *et al*. [25], where topoisomerase I tracks the position of transcribing RNAPs, we extend the model above (as in ‘Topoisomerase activity on DNA’ section) so that the presence of transcribing RNAPs increases the binding rate of DNA topoisomerase I. The expanded binding model for topoisomerase I is of the form:


(15) \begin{eqnarray*}
k_\mathrm{topoI}(\sigma , r) = \left\lbrace \begin{array}{@{}l@{\quad }l@{}}k_\mathrm{topoI}(\sigma ) & \text{if } r > d \\\alpha _{E} k_\mathrm{topoI}(\sigma ) & \text{if } r \le d \end{array}\right.,
\end{eqnarray*}

where $r$ is the downstream distance between the topoisomerase I binding site and transcribing RNAP (e.g. topoisomerase I follows behind RNAP) $k_\mathrm{topoI}(\sigma )$ is binding rate as in equation (4), $d$ is an effective distance, and $\alpha _E$ is a multiplier that increases the binding rate of topoisomerase I in the vincinty of RNAP. The binding of topoisomerase I is now influenced by both the superhelical density within the DNA and by the presence of transcribing RNAPs.

### Platform model

We now turn to how the domain model above is implemented as a computer simulation by defining a computational platform model.

#### The biomacromolecules within TORCphysics

**Figure 3.**
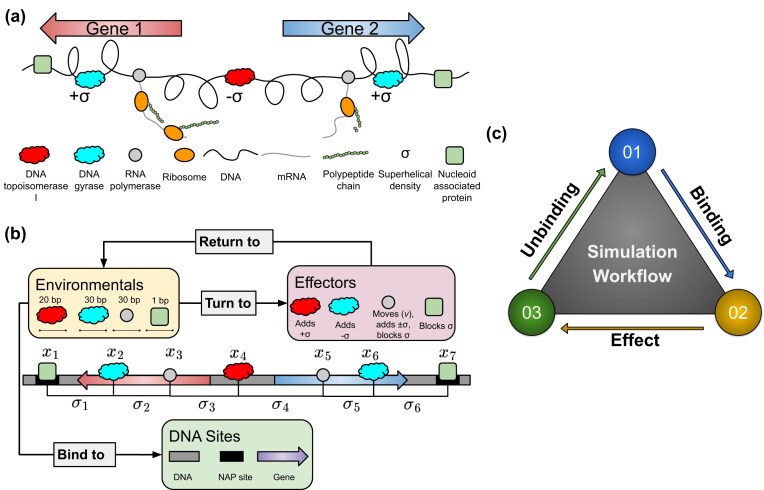
(**a**) Schematic representation of transcription-supercoiling coupling in bacteria using a two-gene system as an example, where RNAPs induce positive supercoils ahead and negative supercoils behind during transcription. (**b**) TORCphysics representation of the transcription-supercoiling coupling in the same two-gene system, where bound proteins are associated with positions $x_i$ and define topological domains characterized by superhelical densities $\sigma _i$. Translation and transertion are not simulated in TORCPhysics; therefore, elements involved in these mechanisms, such as ribosomes, polypeptide chains, and RNA degradation, are not included. (**c**) TORCphysics simulation workflow, where system integration is achieved through cycles of protein binding, effect, and unbinding.

Figure 3a and b illustrates the various biomacromolecules considered within the TORCphysics framework and the physical abstraction we use to represent each one. The DNA is modelled in one dimension, and since it cannot writhe, the only contribution to supercoiling is due to twist. Within this model, the DNA can interact with RNAPs, DNA topoisomerase I, DNA gyrase and nucleoid-associated proteins (NAPs), in a supercoiling-dependent manner. DNA-bound molecules are characterized by a position $x_i$ and are associated with a superhelical density $\sigma _i$. Protein binding sites can be sequence-specific with well-defined positions (e.g., promoters for RNAPs) or non-specific, allowing proteins to bind anywhere along the DNA (as in the case of topoisomerases). Transcription-supercoiling coupling is captured by considering the supercoiling induced by transcribing RNAPs in accordance with the twin-supercoiling domain model [15], and we assume that all bound proteins, except topoisomerases, act as topological barriers preventing supercoiling diffusion. Although there are no direct interactions between bound molecules modelled within TORCphysics (unless explicitly defined through a specific sub-model), they interact indirectly because they all both affect and are affected by the superhelical density $\sigma$.

TORCphysics predicts the transcriptional output of a gene as a function of time by considering the evolution of the system in blocks of time $\Delta t$ ($\Delta t = 1.0$ s) during which proteins can bind, affect the DNA or unbind. Transcribing RNAPs move fowards with velocity $v$ inducing supercoils and acting as moving barriers. In contrast, topoisomerases and NAPs are stationary: topoisomerases locally modify the superhelical density, whereas NAPs block the diffusion of supercoils. In this work, we do not explicitly model the binding and unbinding of NAPs, but we assume they are always present at the ends in the gene architecture experiments. Since transcription occurs at timescales in the order of minutes/seconds, and twist diffuses much faster than plectonemes (on the range of kb$^2$/s) [47], we assume that supercoiling propagates and equilibrates instantly (within one timestep) in a given region.

Binding and unbinding probabilities of biomacromolecules to a specific DNA site over time $\Delta t$ are assumed to be independent random events, and so are modelled as Poisson processes [48]


(16) \begin{eqnarray*}
P = e^{- k(\sigma )\Delta t} k(\sigma ) \Delta t,
\end{eqnarray*}

where the rate $k(\sigma )$ may be modulated by the local superhelical density $\sigma$. In the present study, these events, as well as transitions between closed- and open-complex formation and transcription initiation, are modelled as stochastic processes. These constitute the primary sources of randomness in the simulations. In contrast, all other mechanistic effects are treated deterministically. For example, the motion of elongating RNAPs and their twist injection (equations 10 and 11), as well as the supercoiling introduced by topoisomerases (equations 6 and 7), are deterministic processes as long as the respective biomacromolecules remain bound to DNA.

#### Simulation workflow in TORCphysics

In order to quantify the interactions between DNA and biomacromolecules such as RNAPs, topoisomerases, and NAPs, our simulation workflow as defined within the TORCphysics framework consists of three stages: binding, effect, and unbinding (see Fig. 3c), described below. These stages compute the binding and unbinding of molecules in the environment (the environmentals) to DNA sites by way of their assigned binding and unbinding models within TORCphysics. When bound, these molecules become effectors. Each effector has an assigned effect model in TORCphysics, which defines how it interacts with DNA. By iterating over these stages, TORCphysics simulates the interaction between the biomacromolecules present and the DNA.

TORCphysics does not currently include reactions within the environment nor depletion of *environmentals*, so the concentrations of *environmentals* remain constant throughout the simulation. The exception is mRNA because each transcription event produces one mRNA molecule, which accumulates in the environment without degradation. While translation, mRNA degradation, and environmental depletion in general are biologically important, these require extensions to TORCphysics (level 3 modifications; see Supplementary Methods, Section 3.3).

##### *Binding* within TORCphysics

During the binding stage of the TORCphysics workflow, we consider the interaction between the DNA and its input environment, which is composed of the environmentals (e.g. topoisomerases, RNAPs, NAPs). These environmentals all have distinct properties: (i) concentrations $E$, (ii) sizes $l$, (iii) DNA binding/unbinding behaviour, and (iv) specific effector models. The concentrations and sizes are shown in Table 1. The current version of TORCphysics does not account for environmental interactions that may influence transcription and protein binding, such as chemical reactions or molecular crowding. All binding models considered in this work are supercoiling dependent. Topoisomerases bind DNA non-specifically according to the TORCphysics model, and so the workflow considers the DNA as split into discrete regions of 20 base pairs for topoisomerase I [49], and 30 base pairs for gyrase [50], and assesses the probability of binding to each using equations (4) and (5), respectively. These sizes were chosen based on crystal [49] and cryo-EM [50] structures of *E. coli* topoisomerase I and gyrase bound to DNA. RNAPs bind only to promoters before they progress along the gene (as in ‘One-step superhelical-dependent transcription model’ and ‘Three-step superhelical-dependent transcription model’ Methods Sections). Promoters/genes are defined as specific DNA sites having start and end positions, directionalities, and particular binding rates/models, such as the one-step or three-step superhelical-dependent transcription models (see equations 8 and 12). NAPs can be modelled to bind to specific DNA sites but have no directionality. The probability of these events is quantified through equation (16).

##### *Effect* within TORCphysics

Once environmentals (e.g. topoisomerases, RNAPs, and NAPs) have bound, they become effectors that have a mechanical impact on the local structure of the DNA: (i) position $x$ on the DNA, (ii) excluded volume on the DNA due to their size $l$ , and (iii) they define a topological domain with associated twist $\phi$ and supercoiling $\sigma$ (see Fig. 3b). Within TORCphysics, the mechanical impact of effector $i$ is quantified according to their change in position along the DNA $\Delta x_i$ and change in local twist $\Delta \phi _i$. Topoisomerases acting as effectors do not move along the DNA ($\Delta x_i=0$), but do change the twist according to equations (6) and (7). RNAPs act as topological barriers moving with velocity $v$ while injecting negative supercoils upstream and positive downstream (see equation 11). NAPs do not move and do not introduce twist, but they do block supercoil diffusion. Other NAPs, such as LacI, can form DNA–NAP–DNA bridges that create two topological domains. While TORCphysics is capable of modelling this behaviour, it is not explored in the present study.

##### *Unbinding* within TORCphysics

The unbinding of *effectors* is determined by the unbinding models. Topoisomerases unbind spontaneously according to an unbinding rate $k_\mathrm{off}$ (see Table 1). RNAPs unbind when they reach a transcription termination site or may unbind before transcription initiation within the three-step transcription model (see the ‘Three-step superhelical-dependent transcription model’ section and Fig. 2c).

#### Topological domains and barriers

We define topological domains as regions with an associated superhelical density $\sigma _i$. The trapped twist $\phi _i$ within a domain is evenly distributed across all base pairs between $x_i$ and $x_{i+1}$, where these coordinates correspond to the topological barriers of the region. Thus, the length of the domain is given by $x_{i+1} - x_i$. Figure 3b illustrates how topological domains are represented in the TORCphysics model, where bound effectors establish the coordinates and local superhelical densities that describe the state of the genetic circuit.

Since bound proteins define the coordinates $(\sigma _i, x_i, x_{i+1})$ of a topological domain, many domains are dynamic, with mobile boundaries and variable supercoiling. RNAPs, for example, can act as mobile barriers. In contrast, NAPs such as HU, IHF, or dimerized LacI can create domains with static boundaries $x_i$ by wrapping, bending, or holding two DNA segments together [9]. TORCphysics does not explicitly model DNA bending, but these proteins are assumed to block the propagation of supercoils. The trapped supercoils may subsequently be modified by other biomacromolecules. Topoisomerases can also bind spontaneously, defining new $(\sigma _i, x_i)$ coordinates. However, they are not considered domain creators, as their action induces twist on both sides of their binding site. For instance, topoisomerase with position $x_4$ in Fig. 3b relaxes supercoils on both sides, decreasing both $\sigma _3$ and $\sigma _4$.

#### Plasmid and chromosomal DNA configurations

TORCphysics can simulate both bacterial chromosomal DNA and plasmids. While bacterial chromosomes are predominantly circular, at the local level the DNA can be treated as a linear structure with fixed boundaries, that neither move nor allow supercoils to escape. Supplementary Fig. S2a shows an example of such a system, where $x_1$ and $x_7$ represent the circuit boundaries, assumed to be NAPs. These boundary NAPs remain bound throughout the simulation. In this configuration, transcription is more susceptible to supercoiling because the boundaries cause rapid accumulation of supercoils generated by elongating RNAPs: negative supercoils build up upstream of transcription, while positive supercoils accumulate downstream. Plasmids, on the other hand, are modelled as circular structures with no fixed boundaries (see Supplementary Fig. S2b). Since TORCphysics is a one-dimensional model, circularity is mimicked using artificial mobile boundaries defined by $x_0=x_N+1$ and $\sigma _0=\sigma _{N}$, where $N$ is the number of bound proteins. Supplementary Fig. S2b illustrates this method with $N=2$. The positions of the artificial boundaries, $x_0$ and $x_3$, are updated at every time step so that the distances from $x_0$ to $x_1$ and $x_2$ to $x_3$ correspond to the distance from $x_2$ to $x_1$ (circling around from left to right). Therefore, the left artificial boundary $x_0$ mimics the last bound effector$x_{N=2}$ while the right boundary $x_{N+1=3}$ mimics the first bound effector$x_1$. Similarly, the superhelicity $\sigma _0$ is updated to match the superhelicity trapped between $x_2$ and $x_3$, i.e. $\sigma _0=\sigma _{N=2}$. In these circular systems, transcription is less sensitive to supercoiling, since the supercoils induced during elongation propagate in both directions around the plasmid and cancel each other out. This behaviour emerges naturally from the TORCphysics model. The ‘Calibrating the stochastic activity of topoisomerases from experimental data’ section implements the plasmid configuration, while ‘Calibrating promoter supercoiling responses from genetic architecture experiments’ and ‘Calibrating the tracking of RNAP by Topoisomerase I from ChIP sequencing data’ sections implement linear configurations. It is well established that gene expression can be different for DNA on plasmids and embedded in the bacterial chromosome, predominantly due to transcription-supercoiling coupling [51]. This difference arises from genetic geography, where the placement and orientation of neighboring promoters and topological barriers shape the local superhelical landscape, which can directly influence gene expression. As a result, when chromosomal promoters are placed into plasmids, they may present different behaviour, which in principle can be takin into account in models where supercoiling and transcription are coupled (for example, the modelling work from Houdaigui *et al*. [14] and Grohens *et al*. [52]).

### Simulation platform

Here, we explain how TORCphysics is used to perform computational experiments. We first define the system to be simulated, we then find the biophysical parameters through a calibration process, and we then use the optimal parameter set to perform the final simulation, which provides quantities such as the production rate of RNA from the genetic circuit, the population of biomacromolecules along the DNA, and the superhelical densities within the genetic circuits. The calibration processes use a random search algorithm to estimate the best set of biophysical parameters to describe the system. The stochastic nature of the Poisson processes used to represent binding and unbinding events means that it is necessary to perform multiple (typically 100) independent simulations for each set of parameters during the search. Simulations of a typical genetic circuit containing 5 kb over a simulation time of 2 h require several minutes to run in real time on a single CPU. Therefore, each computational experiment requires ~2400 CPU h.

#### Calibrating the stochastic activity of topoisomerases from experimental data

**Figure 4.**
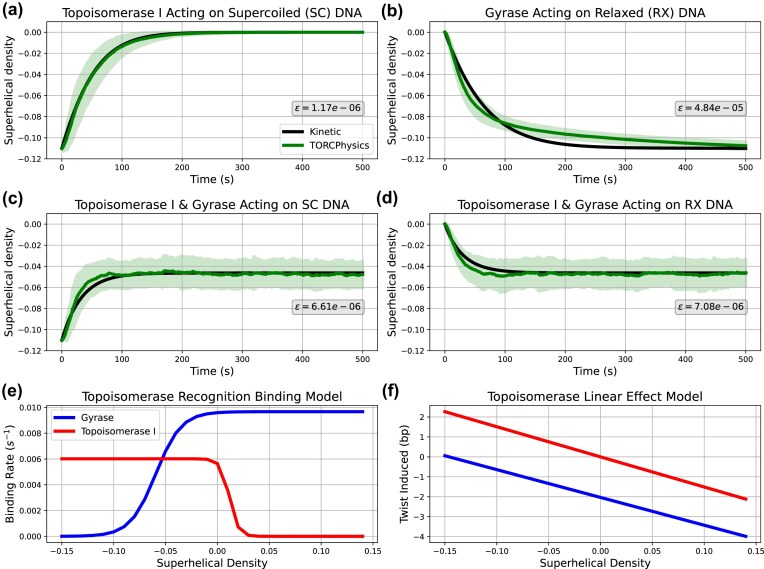
Stochastic topoisomerase activity parameterized from experimental data [7]. TORCphysics results using the best parameter set from the calibration process (described in section ‘Calibrating the stochastic activity of topoisomerases from experimental data’) in green, compared with kinetic curves inferred from experimental data in black, across different scenarios: (**a**) Topoisomerase I acting on negatively supercoiled DNA, (**b**) gyrase acting on relaxed DNA, (**c**) both topoisomerases acting on negatively supercoiled DNA, and (**d**) both enzymes acting on relaxed DNA. Shaded green areas represent the standard deviation obtained from multiple simulations. Superhelical-dependent binding rates (**e**) from equations (4) and (5), and rates of twist (**f**) from equations (6) and (7).

Here, we calibrate the biophysical models of the stochastic activity of topoisomerases on superhelical DNA. Wang *et al*. [7] used a fluorescent marker to quantify how fast topoisomerase I and gyrase relax and negatively supercoil DNA, respectively, and show that both topoisomerases exhibit classic Michaelis-Menten kinetics (see Supplementary Table S2). In Supplementary Methods, Section 7, we show how we construct reference curves for the change in DNA superhelical density $\sigma _\mathrm{kinetic}$ with time for the two topoisomerases (see Supplementary Figs S3 and S4), both individually and in combination (shown in Fig. 4). To discretize these kinetic processes in time for use in TORCphysics models, we simulated an equivalent plasmid (2757 bp), topoisomerase concentrations (see Table 1) and timescale $T$ ($T=500$ s) as used by Wang *et al*. [7] to obtain a DNA superhelical density $\sigma _\mathrm{TP}$. We tested 8000 random parameter sets for equations (4)–(7), and for each performed an ensemble of 100 repeat simulations, calculating an average superhelical density $\sigma _\mathrm{TP}$ per ensemble. We then selected the set of parameters that best fit the reference curves $\sigma _\mathrm{kinetic}$ by minimizing the objective function


(17) \begin{eqnarray*}
\epsilon (z) = \sum _{i=1}^{4} \sum _{j=1}^{T} \left| \sigma _{\mathrm{kinetic}, i, j} - \sigma _{\mathrm{TP}, i, j}(z) \right|^2,
\end{eqnarray*}

where $z$ denotes to the parameter space, $j$ labels the time data points (1 to $T$), $i$ labels four scenarios: (i) topo I acting on negatively supercoiled DNA, (ii) gyrase on relaxed DNA, (iii) both enzymes acting on supercoiled DNA, and (iv) both enzymes acting on relaxed DNA (see Fig. 4). The parameter space $z$ includes 11 parameters: binding rates $k_\mathrm{on}$, sigmoid parameters $\sigma _w$ and $\sigma _t$, unbinding rates $k_\mathrm{off}$, twist rates $k_\phi$ and the maximal superhelical density that gyrase can impose $\sigma _0$. Supplementary Fig. S5 shows the distribution of losses, confirming that the calibration process successfully converged to acceptable solutions for the purposes of this work. Additionally, the sensitivity analysis (see Supplementary Methods, Section 2) demonstrates the robustness of the implemented models and shows that variations in individual parameters are consistent with the underlying physical quantities they represent. Lastly, notice that in this experiment there is no transcription, and the introduction or relaxation of supercoils is due solely to the action of topoisomerases.

#### Calibrating promoter supercoiling responses from genetic architecture experiments

We now calibrate promoter activity using the transcription models (presented in the ‘One-step superhelical-dependent transcription model’ and ‘Three-step superhelical-dependent transcription model’ sections) by matching TORCphysics outputs with the changes in gene expression rates as a function of gene architecture measured by Boulas *et al*. [18]. Those experiments used a plasmid containing two genes coding for fluorescent proteins, where one is flanked by two LacI binding sites. When bound, the LacI-looping protein isolates the inner gene. That experiment studied how the ratio of fluorescence changes when different DNA fragment lengths are inserted between the upstream barrier and the promoter of the enclosed gene. The experiment investigated three promoters with different strengths, ranging from weak to strong (see promoter sequences in Supplementary Table S3). For all three promoters, they observed that gene expression increases as the upstream barrier is positioned further away.

To evaluate the ability of TORCphysics to reproduce those experimental observations, we performed simulations for circuits composed of flanking barriers (modelled as NAPs) with a single gene in between, where the shortest distance between the upstream barrier and the promoter is 101 bp and the longest 5051 bp, and the gene length is 900 bp. The stochastic activity of topoisomerases is modelled using the parameterization described above (as in the ‘Calibrating the stochastic activity of topoisomerases from experimental data’ section). To mimic transcription, we consider three physical models of increasing complexity:

- V0: One-step transcription model.
- V1: Three-step transcription model.
- V2: Three-step transcription model with topoisomerase I RNAP tracking.

Since both the one- and three-step transcription models have expressions that depend on the free energy required to melt DNA ($U_{\mathrm{melt}}$), which depends on both the DNA sequence and the superhelical levels, we first computed the stress-induced DNA duplex destabilization profiles (SIDD) [12] for the three promoter sequences before beginning the calibration process. To achieve this, we added a flanking sequence of 250 G/C base pairs on each side to each promoter sequence. Using the SIST algorithm [13], we calculated the free energy required to melt each promoter at various superhelical densities (see Supplementary Fig. S6). Finally, we fitted a sigmoidal function to model open complex formation as a function of the melting energy (see Supplementary Fig. S7).

We then perform a calibration process for the three promoters, running 60 simulations lasting 1.5 h of simulation time, for each upstream distance $i$. From these, we calculate the susceptibility $s$, defined as the expression rate $k$ divided by the expression rate at a reference upstream distance $s_i=k_i/k_{i=\mathrm{ref}}$. We select the reference upstream distance 250 bp for comparison with Boulas data [18]. We conducted 300 random tests for V0 and 1300 for V1 and V2, evaluating each test performance according to the objective function


(18) \begin{eqnarray*}
\epsilon (z) = \sum _{i=1}^{l} \left| s_{\mathrm{exp},i} - s_{\mathrm{TP}, i}(z) \right|^2 + \left| \hat{\sigma }_{\mathrm{exp},i} - \hat{\sigma }_{\mathrm{TP}, i}(z) \right|^2,
\end{eqnarray*}

where $s_{\mathrm{exp},i}$ denotes the experimental averaged susceptibility for the upstream distance $ i$, $ s_{\mathrm{TP},i}(z)$ is the corresponding simulated susceptibility, $\hat{\sigma }_{\mathrm{exp},i}$ is the experimental standard error and $\hat{\sigma }_{\mathrm{TP},i}$ is the simulation standard error. The parameter space $ z$ includes the promoter rate $k_\mathrm{on}$ in case of V0 (one parameter to fit), while for V1 and V2 it includes the promoter rates of the three-step transcription model, as well as the width $ \sigma _e$ and threshold $\epsilon _e$ from the elastic function $ G_{\mathrm{elastic}}$ (seven parameters to fit, see Equations (12) and (13); Fig. 2c). Since no experimental measurements of the absolute expression rates were provided, these cannot be included in the calibration process. Therefore optimization is performed for the susceptibility and then solutions are selected based on how well they reproduce the relative expression rates observed in the experimental data.

Finally, for V0 and V1, the twist injection rate parameter $\gamma$ is calibrated independently by increasing $\gamma$ from 0.01 to 1.0, whereas in V2 it is obtained through the topoisomerase I tracking protocol (see ‘Calibrating the tracking of RNAP by Topoisomerase I from ChIP sequencing data’ section). For V0, the optimal solutions are found with $\gamma = 0.05$, for V1 they are found with $\gamma = 0.1$. Supplementary Fig. S8 presents the distribution of losses across the three physical models (V0, V1, and V2), demonstrating that the calibration processes successfully converged to acceptable solutions for the purposes of this work. Lastly, the sensitivity analysis (see Supplementary Methods Section 2 and Supplementary Figs S9 and S10) indicates that for all three models (V0–V2), gene expression is highly sensitive to variations in promoter kinetics, either in the form of binding/opening rates and/or the geometric modulations for the closed-complex formation (spacer length and flexibility). The parameterization of each promoter allows for a unique responsiveness to supercoiling, demonstrating that the three-step transcriptional model is well suited to model promoter diversity.

#### Calibrating the tracking of RNAP by topoisomerase I from ChIP sequencing data

The ChIP-Seq data obtained by Sutormin *et al*. [25] suggests that DNA topoisomerase I tracks transcribing RNAPs in *E. coli*. To assess the importance of this phenomenon on gene regulation, we include this interaction within TORCphysics using the three-step transcription and the tracking topoisomerase I models (as in sections ‘Three-step superhelical-dependent transcription model’ and ‘Modelling the tracking of RNAP by topoisomerase I’, respectively).

**Figure 5.**
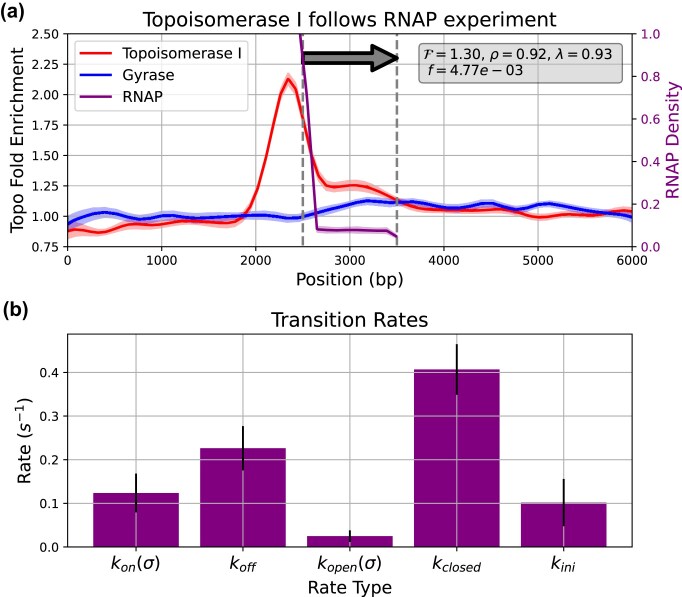
(**a**) Fold enrichment profiles for topoisomerase I (red), gyrase (blue), and RNAP normalized density (purple), obtained from the topoisomerase I tracking RNAP calibration process (described in section ‘Calibrating the tracking of RNAP by Topoisomerase I from ChIP sequencing data’), using the optimal set of parameters. The grey arrow represents the transcription unit (TU). Parameter $\mathcal {F}$ denotes the mean fold enrichment of topoisomerase I within the TU, $\rho$ represents the correlation coefficient between RNAP and topoisomerase I positions, $\lambda (z)$ indicates the correlation between RNAP density in TORCphysics and ChIP-seq data from Sutormin *et al*. [25], and $f$ represents the loss function (see equation 19). (**b**) Transition rates used in the three-step superhelical-dependent transcription model (see Fig. 2). Bars indicate average values, and error bars represent standard deviations across the top 5% of best calibration sets. Both $k_\mathrm{on}$ and $k_\mathrm{open}$ rates are superhelical density dependent ($\sigma )$.

To mimic the ChIP-Seq analysis, we simulated a 6000 bp sequence (this is the typical size of a topological domain found in *E. coli* [53]) containing an averaged sized gene of 1000 bp [54] (see Fig. 5). We assume the DNA ends are constrained by topological barriers, and that the superhelical density can be modified only by the action of topoisomerases. We use the parameters for topoisomerase activity previously obtained (see the ‘Calibrating the stochastic activity of topoisomerases from experimental data’ section), set the initial superhelical density to $\sigma =-0.046$, and run each simulation for 1000 s (16.6 min) of simulation time.

We quantify the relationship between the transcribing RNAPs and DNA topoisomerase I by calculating the Pearson correlation coefficient $\rho$. Using ChIP-Seq [25] to deduce the two enzyme position densities within the transcribing unit (TU) gives $\rho =0.94$ (see Supplementary Fig. S11). The fold-enrichment $\mathcal {F}$ compares the topoisomerase I activity in the presence of transcribing RNAPs with the case where there is no transcription. Calculating an average over the TU from the ChIP-Seq data gives $\mathcal {F}=1.24$ (see Supplementary Fig. S11). We also calculate the correlation coefficient $\lambda$ between the simulated RNAPs and the experimental RNAP signal within the TU to parametrize the action of transcribing RNAPs using the three-step transcription model (see the ‘Three-step superhelical-dependent transcription model’ section). We implement the following four step calibration protocol:

- Step 1: We start with a reference system where transcription is turned off and only topoisomerase I and gyrase interact with DNA by way of the stochastic topoisomerase model. We launch a set of eight simulations and calculate position densities for both enzymes. Each simulation runs for 1 h of simulation time.
- Step 2: Transcription is now turned on, and we perform 3000 random tests with different parameterizations to calculate the objective function $f(z)$ (see equation 19). For each test, we run 28 sets composed of eight simulations each, which are combined to give 28 independent histograms describing the position densities for RNAP, topoisomerase I, and gyrase. The fold-enrichments $\mathcal {F}$ are computed and averaged within the TU, and the mean position densities are obtained per molecule (RNAP, topo I, and gyrase). We build 28 independent histograms to exploit the parallelization capabilities of the HPC cluster and to reduce stochastic variability. Averaging across multiple histograms provides more stable estimates of the fold-enrichment $\mathcal {F}$ and the correlation coefficient $\rho$, ensuring that the calibration procedure is not biased by an individual simulation (see Supplementary Fig. S12).
- Step 3: The mean densities are used to compute the correlation coefficient $\rho$ between RNAP and topoisomerase I.
- Step 4: The calibration process is complete when the parametrization set that minimizes the objective function has been determined.

The objective function minimsed during the calibration is


(19) \begin{eqnarray*} f(z) = (\mathcal {F}_\mathrm{exp} - \mathcal {F}_\mathrm{TP}(z))^2 + (\rho _\mathrm{exp} - \rho _\mathrm{TP}(z))^2 + (1 - \lambda (z))^2, \end{eqnarray*}

where $z$ denotes the parameter space to be calibrated, $\mathcal {F}_\mathrm{exp}=1.24$ is the mean fold-enrichment of topoisomerase I within the TU measured experimentally, $\mathcal {F}_\mathrm{TP}(z)$ is the fold-enrichment calculated from simulations, $\rho _\mathrm{exp}=0.94$ is the experimental correlation coefficient between RNAPs and topo I, $\rho _\mathrm{TP}(z)$ is the correlation coefficient from the simulations, and $\lambda (z)$ is the correlation between the experimental RNAP density and the density within the TU region obtained from simulations.

The parameter space $ z$ comprises the effective distance $ d$ and multiplier $ \alpha _E$ from the topoisomerase I tracking model (equation 15), the rate of twist $ \gamma$ induced during transcription (equation 11), and the promoter rates $ k_{\mathrm{on}}(\sigma )$, $ k_{\mathrm{off}}(\sigma )$, $ k_{\mathrm{open}}(\sigma )$, $ k_{\mathrm{closed}}$, and $ k_{\mathrm{ini}}$ for the three-step transcription model (see Fig. 2c). For the elastic function $ G_{\mathrm{elastic}}$ used in the three-step transcription model, we select the values $ \sigma _e = -0.06$ and $ \epsilon _e = 1/\sqrt{ n k_\theta \alpha ^2}$ (see Supplementary Methods, Section 6). This parametrization is equivalent to the spacer length model proposed by Forquet *et al*. [10]. For the melting energy function $ U_{\mathrm{melt}}$, we use $ \sigma _m = -0.042$ and $ \epsilon _m = 0.005$, which correspond to those used in the thermodynamic model from El Houdaigui *et al*. [14] for the *pelE* promoter [55]. We use this fixed parametrization to represent the supercoiling response of an average bacterial promoter. The distribution of losses shown in Supplementary Fig. S13 demonstrates that the calibration process presented here converged to acceptable solutions. Lastly, the sensitivity analysis (see Supplementary Methods Section 2 and Supplementary Fig. S14) confirms the robustness of the model, as variations in individual parameters lead to consistent physical effects without fundamentally altering the behaviour of the system.

## Results and discussion

### Modelling the stochastic activity of DNA topoisomerase I and DNA gyrase on supercoiled DNA

In bacteria, the activity of topoisomerase enzymes is crucial for maintaining an adequate negative superhelical level [2–4]. Here, based on the kinetic parameters measured by Wang *et al*. [7] in *E. coli*, we have derived a discretized biophysical model for the stochastic activity of topoisomerases on supercoiled DNA.

Figure 4 shows the superhelical densities inferred from the steady-state kinetics [7], compared with the average superhelical densities obtained from simulations using the optimal parameter set from the calibration process (see Table 1). Overall, there is strong agreement between the experimentally determined kinetics and the TORCphysics results, with relatively small mean squared errors on the order of $ \epsilon \approx 10^{-5}$ for all four cases. For topoisomerase I acting on supercoiled DNA, the model more accurately captures the relaxation curve, with a very small error ($ \epsilon \approx 10^{-6}$; see Fig. 4a). In contrast, for gyrase acting on relaxed DNA (Fig. 4b), the model generally reproduces the kinetic behaviour but tends to overestimate the amount of torsion (negative supercoils) introduced. Topoisomerase I acts faster than gyrase, completely relaxing hyper-negative supercoils within 100 s, while gyrase takes ~200 s to negatively supercoil the DNA. In the combined scenarios where both enzymes act on supercoiled/relaxed DNA (Fig. 4c and d), the model performs well over longer timescales, although there is an overestimation of the superhelical density in the first 100 s before the steady-state plateau is reached. Lastly, Supplementary Fig. S15 shows the approximate changes in twist and writhe calculated using the partition method (described in ‘Calculating the superhelical density within TORCphysics’ section). The calculations result in relaxed structures with $\Delta \mathrm{Wr} = 0$ when topoisomerase I acts alone, because topoisomerase I relaxes supercoils. However, when gyrase acts alone, highly plectonemic structures with $\Delta \mathrm{Wr} \approx -20$ arise because gyrase can only introduce negative supercoils. Plectonemic structures with $\Delta \mathrm{Wr} \approx -10$ arise when both topoisomerases act together. These values are consistent with writhe ($\Delta \mathrm{Wr} = -9.4$) measurements of plasmids of similar size (2686 bp) and comparable superhelical density ($\sigma =-0.047$, $\Delta \mathrm{Lk} = -12$) [45], supporting the suitability of this approximation for comparison with experimental data.

Figure 4e and f show the binding rates and twists induced by DNA topoisomerase I and gyrase obtained from the calibration process. For the grid sizes, enzyme concentrations, and kinetic rates in Table 1, and at superhelical densities where they are fully active, we observe that both enzymes bind to the DNA at an approximate rate of one every 2 s, and remain bound for around 5 s before unbinding. A binding event every 2 s aligns with single-molecule experiments of DNA gyrase activity *in vivo* [57] and is 3 to 5 times more frequent than values used in previous stochastic models [40] (see Table 1). In terms of unbinding, our predictions are about five times higher than in previous models calibrated for *E. coli* [40]. However, our values are consistent with single-molecule experiments of gyrase activity around the replisome [57]. The set of estimated parameters indicate that both DNA topoisomerase I and gyrase maintain significant presence on the DNA across physiological superhelical densities $\sigma =[-0.11, 0.05]$, and that both enzymes exhibit similar behaviour in terms of binding and unbinding rates, as well as catalytic activity $k_\phi$ (see Table 1). Experimental and theoretical studies have also reported that both enzymes have comparable activity [7, 14, 40] and propose sigmoidal functions to model activity. Although the threshold parameters $\sigma _t$ used are different, the width parameter $\sigma _w$ is similar across these studies. For instance, El Houdaigui *et al*. [14] report a width twice as broad as in TORCphysics, while Geng *et al*.’s model [40] yields an almost identical width parameter for gyrase (see Table 1). However, El Houdaigui’s model differs from TORCphysics topoisomerases by acting continuously in every topological domain.

In principle, topoisomerase I could bind to and relax positive supercoils (see equations 4 and 6). Our sensitivity analysis (see Supplementary Figs S16 and S17) indicates that topoisomerase I is unlikely to act upon positively supercoiled DNA (see Supplementary Methods, Section 2). However, the experimental data used for the parameterization currently only includes negatively supercoiled DNA, but this could be updated to include positive supercoiling when the relevant experimental data becomes available. To evaluate the long-time scale behaviour of gyrase, we performed extended simulations lasting up to 3000 s (see Supplementary Fig. S18), which shows that the global superhelical density continues to decrease slowly over time. We tested a parameterization where the asymptote was fixed at $\sigma = -0.11$ (see Supplementary Fig. S19). The model then does not reproduce the relaxation curves for gyrase over shorter times, and provide less favorable agreement with the behaviour seen when gyrase and topoisomerase I act together. Therefore, we favour the initial calibration.

**Figure 6.**
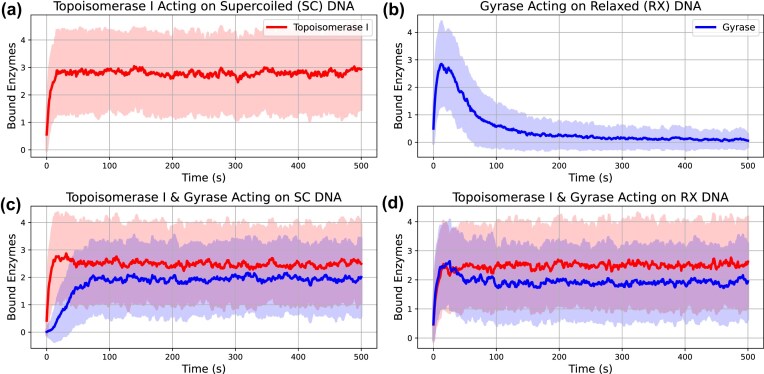
Convergence of the calibration of the stochastic topoisomerase activity from experiments, described in the ‘Calibrating the stochastic activity of topoisomerases from experimental data’ section, using the best parameter set. The plots show the average number of topoisomerase I (red) and gyrase (blue) molecules bound to DNA over time across different scenarios: (**a**) topoisomerase I acting on negatively supercoiled DNA, (**b**) gyrase acting on relaxed DNA, (**c**) both topoisomerases acting on negatively supercoiled DNA, and (**d**) both enzymes acting on relaxed DNA. Shaded areas represent standard deviations obtained from multiple simulations.

Figure 6 shows the number of bound enzymes throughout the calibration process, indicating that the algorithm converges to a fixed value. Note that the transient behaviour at times less than a 100 s has no physical meaning and reflects the convergence of the calibration process. For DNA topoisomerase I, we observe that the DNA remains relaxed in spite of the presence of the enzyme, as expected as topoisomerase I primarily relieves superhelical stress [2] (see Figs 4a and 6a). In contrast, gyrase activity decreases as the DNA becomes hypernegatively supercoiled (see Figs 4b and 6b). The steady-state curves when both enzymes are fully active (Fig. 6c and d) show that ~2.5 DNA topoisomerases and two gyrases are bound to the DNA on average and maintain a steady-state superhelicity of $ -0.046$, which is consistent with *in vivo* observations [58]. This behaviour suggests that at least one enzyme of each type is present per 1 kb of DNA, which aligns with the average gene size in prokaryotes [54].

Single-molecule experiments [59, 60] have shown that gyrase introduces negative supercoils in multiples of two DNA turns and can perform several catalytic cycles before dissociating. In contrast, our current model can change the twist by a non-integral number of turns, which depends on the local superhelical density. Nevertheless, introducing the gyrase multi-kinetic step mechanism into TORCphysics is possible under level 1 modifications (see Supplementary Methods, Section 3) to reproduce single-molecule data [59, 60], where gyrase could introduce finite amounts of twist per catalytic cycles similar to recent modelling approaches [42].

### Impact of gene architecture on expression within the one-step transcription model (V0)

DNA supercoiling and gene expression are intimately linked. Boulas *et al*. [18] investigated this relationship *in vivo* within the simple genomic context of a system consisting of a single gene enclosed within a topological domain. Their experiments showed that gene expression is strongly influenced by the distance between the promoter and the upstream topological barrier, while their biophysical model proposed that topoisomerase I represses initiation and facilitates elongation. Here, we simulate this system with TORCphysics using the calibrated topoisomerase models and the one-step transcription model.

**Figure 7.**
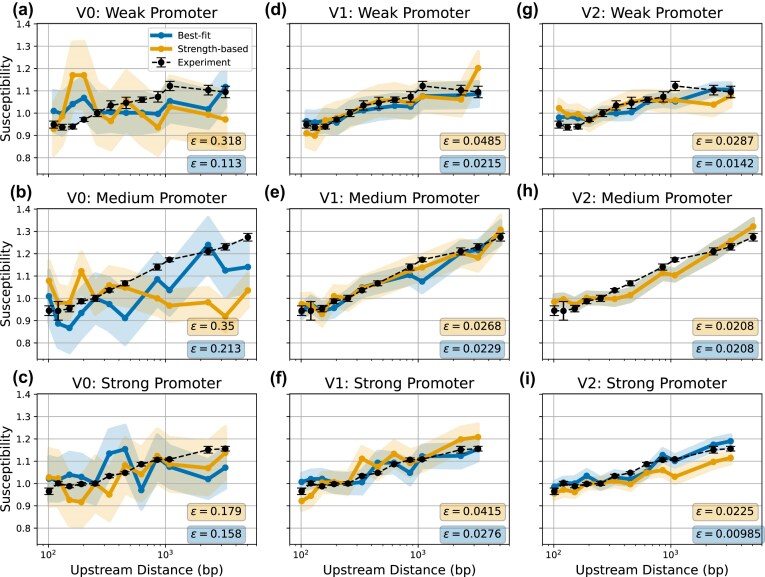
Averaged susceptibility calculated from TORCphysics as a function of upstream barrier distance for the weak, medium, and strong promoters, compared with the experimental susceptibility measured by Boulas *et al*. [18] (black dashed line). Parameterizations that best reproduce the experimental susceptibility are shown in blue, whereas those selected to match the experimental relative expression rates while also capturing the experimental susceptibility are shown in yellow. The TORCphysics results were obtained by the calibration process for three models V0 a–c, V1 d–f, and V2 g–i (as in section ‘Calibrating promoter supercoiling responses from genetic architecture experiments’). The RNAP twist-rate parameter ($\gamma$) was set to 0.05 in V0, 0.1 in V1, and 0.157 in V2. Standard errors are represented as shaded areas for the TORCphysics simulations and as error bars for the experimental data.

Figure 7a–c shows the susceptibility calculated as a function of the distance between the promoter and the upstream barrier following the calibration protocol (as in section ‘Calibrating promoter supercoiling responses from genetic architecture experiments’), for both the best-fit solutions and those selected based on relative expression rates from the experiments (strength-based solutions). Of the three models investigated, V0 gives the least favourable comparison with the experimental data. Gene expression levels are notably low, with the strong promoter producing approximately one transcript every 8 min and the weak promoter one every 30 min (see Supplementary Figs S20a–c and S21a). However, Supplementary Fig. S22d shows that RNAP binding events occur much more frequently, ranging from one event per minute for the weak promoter to one every 25 s for the strong promoter.

**Figure 8.**
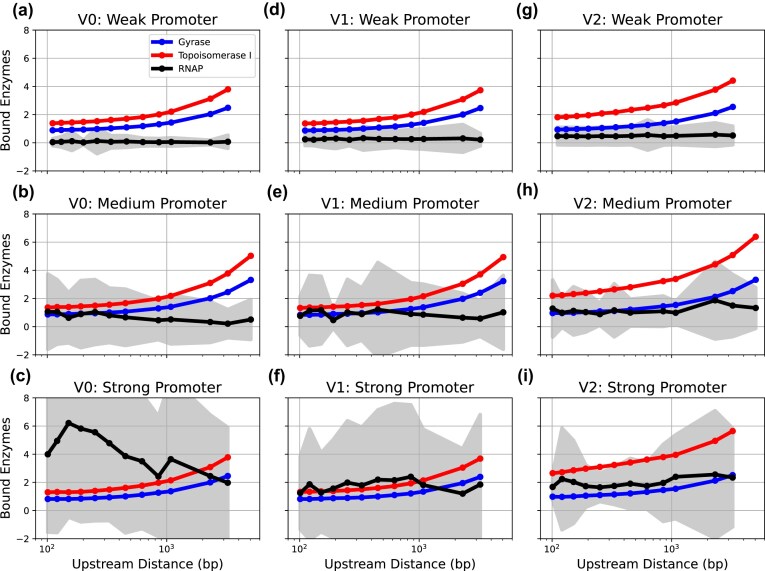
Average number of bound enzymes as a function of the distance between the upstream barrier and the promoter, using the optimal promoter parameterizations for the three models: V0, V1, and V2. Topoisomerase I is shown in red, gyrase in blue, and RNAPs in black, with standard deviations represented as shaded areas.

To understand this discrepancy, we analysed the average number of bound topoisomerases and RNAPs (see Fig. 8a–c). For both topoisomerase I and gyrase, we see a higher number of bound enzymes as the distance between the promoter and the barrier increases, as there is more space available for these molecules to bind. The number of elongating RNAPs is low for the weak and medium promoters because their initiation rates are low and their activation requires a hyper-negative superhelical density (see Supplementary Fig. S22a and S22d). However, for the strong promoter, the number of elongating RNAPs saturates. Although its SIST modulation profile is similar to that of the other promoters, this saturation likely arises from its higher initiation rate combined with the accumulation of supercoils that stall transcriptional activity.

Supplementary Fig. S23a–c shows the superhelical density both globally and at the promoter. The results indicate that both superhelical densities are similar for the weak and medium promoters due to their low initiation rates. In contrast, for the higher initiation rate of the strong promoter, we see that the superhelical densities fluctuate more and that for shorter distances, the superhelical density at the promoter is lower in magnitude compared to the global value, with the two becoming more similar as the distance increases. This behaviour arises from uncontrolled transcription initiation, which produces more transcribing RNAPs (see Supplementary Fig. S22d). At short distances, the rapid accumulation of negative supercoils leads to transcriptional stalling (see Supplementary Fig. S1). Once the machinery stalls, the generation of excessive negative supercoils is reduced, which lowers the average negative supercoiling at the promoter but also decreases transcription. As the distance increases, the buildup of negative supercoils is alleviated, reducing stalling and allowing transcription to proceed more stably. Nonetheless, these results indicate that the one-step transcription model is not capable of representing the experimental system.

### Impact of gene architecture on expression within the three-step transcription model (V1)

We now consider how the expression rate changes with distance between the promoter and the topological barrier using the three-step transcription model within TORCphysics, as shown in Fig. 7d–f. As well as improved agreement with the experimental data, we see from Supplementary Figs S20d–f and S21b that higher expression rates are obtained. One transcript is produced every 11 min by the weak promoter and one every 3 min by the strong promoter. Supplementary Fig. S22e shows the rates obtained for the three promoters that resemble the relative expression rates from experiments and demonstrates that promoter activity depends on a complex balance of kinetic properties, consistent with previous studies [18, 61].

While the number of bound topoisomerases is indistinguishable for V0 and V1, the number of transcribing RNAPs for V1 is lower (on average 2 RNAPs for the strong promoter in V1 compared to 4–6 for the V0 model), which is surprising given that the transcription rate is higher for the three-step transcription model (see Fig. 8d–f). While the one-step transcription model employed in V0 enables faster initiation events, the number of elongating RNAPs saturates for high initiation rates. However, the three-step model in V1 allows the RNAP to pause during initiation, which prevents stalling and leads to a higher gene expression rate. This is reflected in the superhelical density at the promoter, being larger in magnitude than the global value across the three promoters (see Supplementary Fig. S23d–f). For V1, reduced stalling allows RNAPs to continue transcribing and thereby induce additional negative supercoils at the promoter. Nevertheless, the expression rate is still only around twice as fast for the three-step compared to the one-step transcription model.

### RNAP tracking by topoisomerase I

Recent ChIP-Seq studies have shown that *E. coli* DNA topoisomerase I directly interacts with transcribing RNAPs [23–25]. To produce a model of this interaction for use within the TORCphysics framework, we built an average topological domain including a single central gene and implemented the calibration protocol (as in section ‘Modelling the tracking of RNAP by topoisomerase I’). Figure 5a shows the computed fold enrichment for both topoisomerase I and gyrase, as well as the RNAP densities calculated from the simulations using the optimized parameter set. Topoisomerase I shows an enrichment of around 1.3 along the TU, compared to the ChIP-Seq data value of 1.4 reported in [25]. For the correlation coefficient $\rho$ between topoisomerase I and RNAP, the model obtains nearly 92%, compared to the experimental value of 94%. In addition, the models show a 93% correlation between the simulated RNAP densities and the RNAP ChIP-Seq data (see Supplementary Fig. S11). However, our model is not able to reproduce the spike of topoisomerase I and RNAP at the termination site due to the assumption of instantaneous termination. Capturing these spikes would require more detailed models of both Rho-dependent and Rho-independent termination, which are within the capabilities of TORCphysics but beyond the scope of this study (level 1 modifications; see Supplementary Methods, Section 3.1).

The effective distance $d$ and multiplier $\alpha _E$ obtained from the calibration procedure (described in section ‘Modelling the tracking of RNAP by topoisomerase I’) indicate that the RNAP position enhances the binding of topoisomerase I by 18 fold, and this effect extends to between 400 and 500 base pairs behind bound RNAPs (see Table 1). However, the maximum fold-enrichment of topoisomerase I is only around 2, because the topoisomerase I binding rate is so low (see Fig. 5a). The estimated rate of twist injection $\gamma$ induced by elongating RNAPs extracted from our calibration procedure ranges from 0.03 to almost 0.30 (see Table 1). Previous studies have employed detailed models that account for both the balance of torques and transcript length to estimate the amount of twist induced in DNA, while other approaches similar to ours have instead used fixed values of $\gamma$ ranging from 0.01 to 1.0 [37–39], although the exact ratio *in vivo* remains unknown.

Although an effective distance of 400–500 bp may seem large for a molecular interaction, our model does not aim to mechanically reproduce the physical interaction but rather to simulate its functional effect along the TU. This large effective distance emerges from the parameterization procedure associated with the model and not directly from the ChIP-seq data.

Figure 5b shows the promoter rates for the three-step transcription models that resulted from the optimization process in the presence of RNAP tracking by topoisomerase I. The rate-limiting step is open-complex formation $ k_\mathrm{open}$ (which is ~1 event every 35 s). The balance of these rates indicates that there are successive rounds of failed initiation and short-lived intermediate states before successful transcription initiation, consistent with previous observations [62–64]. Moreover, a recent two-step kinetic model based on *in vivo* data by Xiao *et al*. [65] reported that the rate-limiting step is initiation (promoter escape). In the context of our model, their RNAP–DNA step is equivalent to ours during open- and/or closed-complex formation, meaning that in this context, the two models are in agreement. However, open-complex formation is not always a limiting step, as each promoter has its own unique kinetic properties, which together determine promoter strength and response [18, 61].

### Impact of gene architecture on expression in the presence of RNAP tracking by topoisomerase I (V2)

Finally, we use the calibrated three-step transcription model, including RNAP tracking by topoisomerase I, to calculate the gene expression rates as a function of distance between the promoter and the topological barrier, for comparison with the experimental data from Boulas *et al*. [18]. Figure 7g–i shows that the average error in the susceptibility across the three promoters is the least for V2, where the three-step model is employed in combination with the tracking of RNAP by topoisomerase I.

Supplementary Figs S20g–i and S21c show that the average gene expression rates increase approximately threefold from V1 to V2, reaching about one transcript per minute for the strong promoter. The number of bound topoisomerase I enzymes increases by 1 for the weak promoter and by 2 for the medium and strong promoters (Fig. 8g–i), while the number of bound RNAPs decreases in comparison with V0 and V1, and gyrase activity remains unchanged. These results suggest that the RNAP tracking mechanism included in V2 provides around 1.5 extra bound topoisomerase I enzymes on average. This results in increased RNAP elongation, where rounds of transcription are characterized by a single RNAP transcribing at a time for the weak and medium promoters while two are present for the strong promoter. The additional topoisomerase I enzyme reduces the global superhelical stress from a density of −0.05 to around −0.04, while the value at the promoter also decreases from −0.06 to around −0.05 when comparing V1 and V2 models, respectively (see Supplementary Fig. S23g–i). The tracking mechanism provides less superhelical stress at the promoter, which reduces RNAP stalling, thereby increases the transcription rate (see Supplementary Figs S20g–i and S21c).

Taking into account all these features reveals a complex network of mechanisms. Equation (3) implies that negative supercoils accumulate faster when the transcribing RNAPs are closer to the topological barrier. These negative supercoils can both promote transcription (by melting the promoter) or stall transcribing RNAPs due to the increasing levels of torsion. Including the tracking of RNAP by topoisomerase I within the model facilitates the removal of supercoils when RNAP is stalled. When the upstream distance from the promoter to the topological barrier is larger, the buildup of negative supercoiling is slower. RNAPs can advance longer distances before stalling, and topoisomerases have more accessible space for binding and removing the excess supercoiling, in agreement with observations by Goldberg *et al*. [42]. This results in a higher susceptibility and greater gene expression rates. It is possible for a gene to both promote and repress itself through its own transcription, depending on the size of the topological domain.

Additionally, to test how gene expression responds to different topoisomerase levels, we varied the baseline concentrations by $\pm 25\%$ (see $E_\mathrm{topoI}$ and $E_\mathrm{gyrase}$ on Table 1). Maintaining the baseline ratio of topoisomerase concentrations resulted in higher production rates, whereas deviations from this ratio led to reduced expression in the conditions tested (see Supplementary Fig. S24). The number of bound topoisomerase I molecules shows a linear dependence on concentration. However, gyrase activity is influenced by both enzymes because it is more sensitive to fluctuations in superhelical density and therefore indirectly depends on topoisomerase I activity (see Supplementary Fig. S25). These results highlight the complex interdependence of gene expression on topoisomerase levels. Moreover, as gyrase activity is ATP-dependent and closely linked to the bacterial growth phase, more refined models based on experiments that explicitly incorporate ATP availability are required to achieve predictive capability for living systems. Furthermore, the absence of topoisomerase I can lead to the formation of R-loops, which can cause genome instability [66]. Level 2 modifications would allow TORCphysics to model R-loop formation, while Level 3 modifications could enable RNase H-mediated degradation to be modelled (see Supplementary Methods, Section 3). We also tested how changing the distance to the upstream and downstream barrier affects gene expression for promoters of varying strengths and obtained results in broad agreement with Boulas *et al*. [18] (see Supplementary Methods Section 8 and Supplementary Fig. S26).

Our model shares conceptual similarities with the model of Boulas *et al*. [18]. Both yield consistent results showing that topoisomerase I facilitates elongation by relieving excess negative supercoils and assisting stalled RNAPs; however, in Boulas *et al*. [18], topoisomerase I can have an antagonistic effect by repressing open-complex formation. The RNAP tracking by topoisomerase I in our framework is comparable to the specific topoisomerase I activity acting on the upstream region in their study, which is essential to reproduce the experimentally observed susceptibility, as it considerably speeds up the removal of supercoils. In TORCphysics, the tracking mechanism also speeds up elongation by approximately two-fold. It is interesting that this feature naturally arises as essential in both models even though they were calibrated through a different procedure and setup. There are also key differences between the models that allow us to investigate distinct aspects of transcription-driven supercoiling. In TORCphysics, we explicitly model the stochastic binding and action of topoisomerases. A major factor influencing susceptibility in both models is RNAP stalling. Boulas *et al*. [18] introduce a stalling threshold at $\sigma _s = -0.062$, whereas in TORCphysics, stalling emerges from the torque balance mechanism and typically occurs when upstream negative supercoils accumulate beyond $\sigma = -0.0813$. Calibration procedures also differ, resulting in higher number of parameters in TORCphysics. In particular, TORCphysics uses a fitting procedure to deduce the RNAP twist injection ratio $\gamma$. Overall, these differences highlight the importance of developing complementary modelling approaches to study supercoiling-driven gene regulation, and TORCphysics offers a flexible platform for testing and integrating such mechanistic models.

## Conclusion

In this work, we introduced TORCphysics, a simulation framework based on physical models that describe DNA-enzyme interactions to investigate supercoiling-driven transcription in genetic circuits. The novelty that TORCphysics offers is versatility, enabling users to define distinct activity models for different types of macromolecules and DNA sites, such as promoters. Here, we make use of that versatility by testing models with increasing complexity (V0, V1, and V2) to investigate transcription and supercoiling dynamics in single-gene architectures. TORCphysics’ flexible framework allows for the integration and reproduction of diverse regulatory behaviours and builds on previous simulations based on the twin-supercoiling domain model and the response of DNA to twisting stress. TORCphysics also introduces new complexity, such as stochastic topoisomerase activities that depend on local superhelical density. However, this flexibility also requires a large number of fitting parameters that result in many potential solutions that are compatible with experimental data.

Our results highlight the importance of incorporating these key factors, but the model could still be further refined to include more complex mechanics. In particular, the current linear model of gyrase activity assumes constant ATP availability, an assumption that does not always reflect conditions *in vivo*. Because gyrase activity depends on the ATP/ADP ratio, which varies across bacterial growth phases [6, 67], transcription-driven supercoiling is also affected. Experiments where the ATP/ADP ratio, topoisomerase activity, and gene expression are monitored and controlled would allow us to model how this influences the dynamics of transcription and supercoiling across growth phases. Level-3 modifications (see Supplementary Methods, Section 3.3) to TORCphysics are necessary to include mechanisms sensitive to ATP/ADP levels, because changes in the environment are not as yet included in the framework. We will therefore include these extensions in TORCphysics in the future.

We also leveraged ChIP-sequencing data from Sutormin *et al*. [25] to parameterize and simulate the interactions between DNA topoisomerase I and transcribing RNAPs, where we estimated an RNAP twist injection ratio of $\gamma =0.157$, which describes the relative rotation between DNA and RNAPs during elongation. This ratio plays a crucial role in transcription, but its value may depend upon molecular crowding, transcript length, and the presence of translating ribosomes. Genes involved in membrane-tethered transcription, such as *tetA*, commonly used in pBR322-based plasmids, may exhibit a significantly higher twist injection ratio [68]. At the upper limit ($\gamma =1$), elongating RNAPs introduce one linking number for every turn of DNA transcribed. This parameter merits further experimental investigation. In the future, a more sophisticated model of RNAP dynamics may include a time-dependent twist injection ratio $\gamma$, and TORCphysics provides a platform for investigating how this would affect gene expression.

We also estimated an effective distance of 400–500 bp for the interaction of transcribing RNAPs with DNA topoisomerase I. Although this distance may seem large for a molecular interaction, our model does not aim to mechanically reproduce the physical interaction but rather to simulate its functional effect along the TU. An alternative description could be obtained by fitting directly to ChIP-Seq profiles and explicitly modelling the topoisomerase I–RNAP complex as an effector, with the aim of simulating the molecular interaction observed by Vidmar *et al*. [27]. However, such an approach would require level 3 modifications (see Supplementary Methods, Section 3.3) and is beyond the scope of this study. Nonetheless, we consider our current implementation appropriate, as it successfully captures the functional interaction between topoisomerase I and RNAP without explicitly modelling the molecular interaction. These findings highlight the importance of more multimodal modelling approaches, where bioinformatics datasets can serve as a bridge between theoretical models and *in vivo* measurements, while complementary single-molecule experiments are essential for quantifying key DNA-enzyme interactions. Such studies can support the development of models with predictive power, potentially advancing applications in areas like synthetic biology and engineering biology. In this context, we propose TORCphysics as a suitable platform for testing and refining these types of experimental hypotheses.

Lastly, the methodology we developed to infer open-complex formation modulation and promoter kinetic properties can also be applied to experimental systems such as those studied by Boulas *et al*. [18] to further investigate the interplay between transcription and supercoiling. In agreement with their conclusions, we found that single genes are capable of modulating their expression through supercoiling. This modulation arises from a complex interplay between the distance to topological barriers, promoter strength, and the interactions between topoisomerases and RNAPs, where both positive and negative supercoils influence transcription. TORCphysics demonstrates strong potential as a tool for studying these complex regulatory mechanisms and offers flexibility for expansion. For instance, it can be adapted to incorporate additional models and explore a broader range of transcriptional regulation processes, including activation and repression by transcription factors and repressors, DNA looping, and beyond.

## Supplementary Material

gkag126_Supplemental_File

## Data Availability

The TORCphysics code repository is publicly available on GitHub at https://github.com/Victor-93/TORCphysics. The dedicated code branch used to generate all results presented in this manuscript, including the processed data and analysis scripts, is available at https://github.com/Victor-93/TORCphysics/tree/TORCphysics_paper. The TORCphysics software and code are also archived at Zenodo: https://doi.org/10.5281/zenodo.15644874. Further details about the contents of these repositories can be found in Supplementary Methods, Section 1.

## Supplementary data

Supplementary data is1 available at NAR online.
