# Correction to ‘TORCphysics: a physical model of DNA-topology-controlled gene expression’

**DOI:** 10.1093/nar/gkag604

**Published:** 2026-06-09

This is a correction to: Victor Velasco-Berrelleza, Penn Faulkner Rainford, Aalap Mogre, Craig J Benham, Charles J Dorman, Carsten Kröger, Susan Stepney, Sarah A Harris, TORCphysics: a physical model of DNA-topology-controlled gene expression, *Nucleic Acids Research*, Volume 54, Issue 4, 27 February 2026, gkag126, https://doi.org/10.1093/nar/gkag126

In the originally published version of this manuscript, author Carsten Kröger’s affiliation information incorrectly included an additional affiliation, “School of Mathematical and Physical Sciences, University of Sheffield, Hounsfield Road, Sheffield, S3 7RH, United Kingdom”, alongside the correct affiliation.

This error, which occurred during the production process, has now been corrected.

The article has been updated to list the author’s affiliation correctly as:

“Department of Microbiology, School of Genetics and Microbiology, Moyne Institute of Preventive Medicine, Trinity College Dublin, College Green, Dublin, D02 PN40, Ireland”.

