# Supplementary material for: TORCphysics: a physical model of DNA-topology-controlled gene expression
Source: Nucleic Acids Res. 2026 Feb 18;54(4):gkag126. doi: 10.1093/nar/gkag126 (PMC12914363; doi:10.1093/nar/gkag126)
Supplement: gkag126_Supplemental_File [file gkag126_supplemental_file.pdf]

Supplementary Material
TORCphysics: A Physical Model of DNA-Topology-Controlled Gene Expression

Victor Velasco-Berrelleza[1], Penn Faulkner Rainford[2], Aalap Mogre[3], Craig J. Benham[4], Charles J. Dorman[3], Carsten Kröger[3], Susan Stepney[2], and Sarah A. Harris[1]

[1]School of Mathematical and Physical Sciences, University of Sheffield, Hounsfield Road, S3 7RH, Sheffield, United Kingdom
[2]Department of Computer Science, University of York, Heslington, YO10 5DD, York, United Kingdom
[3]Department of Microbiology, School of Genetics and Microbiology, Moyne Institute of Preventive Medicine, Trinity College Dublin, College Green, D02 PN40, Dublin, Ireland
[4]Department of Mathematics, University of California Davis, One Shields Avenue, CA 95616, Davis, California, United States of America

January 29, 2026

Overview

Here we present the detailed methodology used in TORCphysics, which includes:

- TORCphysics code repository — see Section 1.
- TORCphysics sensitivity anaysis - see Section 2
- TORCphysics modification levles - see Section 3
- Parameterising promoter melting energy using the SIST algorithm — see Section 4..
- Marko's elastic model of supercoiled DNA and RNAP velocity — see Section 5.
- Elastic function and spacer model equivalency — see Section 6.

- Calculating global superhelical density from experimental kinetic parameters of DNA topoisomerases — see Section 7.
- Upstream vs Downstream barrier distance variations in gene architecture experiments - see Section 8
- Supplementary Figures - see Section 9.
- Supplementary Tables - see Section 10.

1 TORCphysics Repository

TORCphysics is written in Python, and the source code is available on GitHub at `https://github.com/Victor-93/TORCphysics`. The repository includes the source code, documentation, and three Jupyter notebooks examples.

TORCphysics can be installed using the command pip install TORCphysics, or downloaded manually as a ZIP file. The software can be executed via the command line or used in Python scripts.

The repository provides three basic usage examples as Jupyter notebooks, located in the Examples/ directory:

- Example_1.ipynb — Run single gene simulations and analysis.
- Example_2.ipynb — Run multiple simulations with statistical analysis.
- Example_3.ipynb — Define custom enzyme/site models using built-in models.

We also provide a dedicated branch, `TORCphysics_paper`, to reproduce the results presented in this manuscript. This branch is accessible at `https://github.com/Victor-93/TORCphysics/tree/TORCphysics_paper`. The necessary scripts and data for reproducing, processing, and plotting the results are located in the Experiments/ directory. Each subdirectory contains a `README` file with instructions for running the scripts.

These scripts are organised into three directories:

- `Topokinetics/` — Contains scripts for reproducing the stochastic topoisomerase activity experiment.
- `TopoITracksRNAP/` — Contains scripts for the RNAP tracking by Topoisomerase I experiment.
- `Genearchitecture/` — Contains scripts for the gene architecture experiments, including the preprocess of running SIST to parameterise promoter melting energies.

2 Sensitivity Analysis

We assessed the robustness of both the TORCphysics domain models and the calibration process by performing a sensitivity analysis across the three experiments. Specifically, we used a one-at-a-time approach, varying each calibrated parameter individually. Since parameters are obtained through random search, we have a collection of parameterisations, each scored according to its error (loss function). From this set, we selected the top 5% with the lowest loss and calculated the average value p and standard deviation σ_p for each parameter (see Table 1 in the main text). Each parameter was then varied independently by increasing or decreasing it by one standard deviation ($p \pm \sigma_p$).

Sensitivity was quantified using two measures: (i) the sum of squared errors ($\sum MSE$) resulted from the comparison between simulations and experiments, and (ii) the normalised sensitivity index (NSI), defined as:

$$NSI = \frac{\Delta \sum MSE / \sum MSE}{\Delta p / p} \tag{1}$$

where $\Delta p = (p \pm \sigma_p) - p = \pm\sigma_p$, and $\Delta \sum MSE$ denotes the the sum of squared errors when parameter p was varied.

The interpretation of this index is as follows:

$$NSI \approx 0 \ : \text{ no sensitivity,} \tag{2}$$

$$0 < NSI < 1 \ : \text{ low to moderate sensitivity,} \tag{3}$$

$$NSI \geq 1 \ : \text{ high sensitivity.} \tag{4}$$

2.1 Sensitivity analysis of the stochastic topoisomerase model on supercoiled DNA

Figure S16a shows the sum of squared errors from the original calibration alongside the one-at-a-time parameter variations. While the model is sensitive to changes in parameterisation, the overall error remains low and within the same order of magnitude. The NSI values (Figure S16b) reveal that parameter sensitivity is asymmetric: the width parameter ($\sigma_{w,\text{topoI}}$) of topoisomerase I has little effect on model performance, whereas the unbinding rates (k_{off}) and twist rates (k_ϕ) are among the most influential parameters, together with the threshold σ_t and the maximum superhelical density σ_0 in gyrase. Consequently, the topoisomerase I binding model behaves as if the enzyme binds exclusively to negatively supercoiled DNA, since replacing the sigmoidal binding model with a constant binding rate yields identical superhelical profiles across all four experimental conditions (see Supplemental Figure S17).

Figures S16c–f illustrate that parameter variations have clear physical consequences consistent with their underlying meaning. For instance, changing the unbinding rate

$k_{\text{off,topoI}}$ affects how quickly the superhelical density reaches the plateau when topoisomerase I acts alone on supercoiled DNA: slower unbinding rates lead to faster plateau formation because topoisomerase I remains bound to the DNA for longer. Similar effects are observed when varying the gyrase unbinding or twist rates (Figure S16d). The consistent and physically interpretable responses to parameter changes indicate that the model is robust and that parameter variations directly influence the physical processes they represent.

2.2 Sensitivity analysis of the RNAP tracking by Topoisomerase I model

The sensitivity analysis of the RNAP tracking by topoisomerase I model is shown in Figure S14a–b, presented in terms of the sum of squared errors and the normalised sensitivity index (NSI). The sum of squared errors indicates that variations in individual parameters have a minor effect on the overall error, which remains within the same order of magnitude. The NSI analysis reveals that, although the error is small, the model is highly sensitive to individual parameter variations, with asymmetric effects reflecting the non-linear influence of rate-related parameters. The most sensitive parameters are the RNAP twist rate (γ), the topoisomerase I binding enhancer ($\alpha_{E,\text{topoI}}$) and its effective distance (d_{topoI}), as well as the open complex formation rate (k_{open}).

Figure S14c illustrates the physical consequences of these individual parameter variations on simulation results, confirming that parameter changes produce physically meaningful effects consistent with their interpretation. For example, parameters associated with RNAP dynamics (γ and k_{open}) primarily affect RNAP position density while slightly influencing topoisomerase I fold enrichment, due to its dependence on RNAP activity. Conversely, variations in parameters governing topoisomerase I activity ($\alpha_{E,\text{topoI}}$ and d_{topoI}) mainly impact its fold enrichment. Overall, these results demonstrate that the model is robust and well suited to its intended purpose.

2.3 Sensitivity analysis of promoter dynamics models (V0–V2) in genetic architecture experiments

For the sensitivity analysis of the genetic architecture experiments, we used the promoter parameterisations selected to match the relative expression rates from the experimental data of Boulas et al. [1], and varied each parameter by one standard deviation instead of using the averaged parameter set. In this analysis, we refer to these selected parameterisations as the original parameterisations. The sum of squared errors for the V0 model (see Figure S9a–c) shows that the results of parameter variations remain within the same order of magnitude as the original parameterisation, with considerable deviations when decreasing the low binding rates of the weak and medium promoters. In some cases, parameter variations even reduce the error, as the original parameterisation is not necessarily the optimal one. Nonetheless, the simplest promoter dynamics described by V0 demonstrate that promoter behaviour can change depending on the transition rates. The NSI index highlights this more clearly (see Figure S10a–c), showing consistently high

sensitivity to variations in k_{on}, particularly for decreases in the rate.

For the V1 model (see Figure S9d–f), the sum of squared errors follows a similar trend to that observed in V0, with single-parameter variations generally producing errors of comparable magnitude, except for certain key parameters related to the closed- and open-complex formation. The normalised sensitivity index (see Figure S10d–f) makes this effect clearer, revealing substantial variation in parameter sensitivity across the three promoters. In particular, decreasing the k_{open} rate and the width parameter ϵ_e are among the most influential changes.

The sensitivity analysis of promoter responses in the V2 model yields comparable results to those obtained for V0 and V1, with the sum of squared errors usually remaining within the same order of magnitude (see Figure S9g–i) and exhibiting diverse parameter sensitivities (see Figure S10g–i). Notably, increasing the threshold σ_e in the weak and strong promoters significantly increases the error (see Figure S9h), a trend that is also reflected in the corresponding NSI (see Figure S10h). Decreasing this parameter produces strong sensitivity in the medium promoter as well (see Figures S9h and S10h).

Taken together, these analyses highlight that promoter dynamics are highly sensitive to promoter kinetics, both in terms of the transition rates and the shape of the Gaussian function modulating closed-complex formation. Unlike the topoisomerase and RNAP dynamics models discussed earlier, this high sensitivity does not compromise the model robustness. Instead, the three-step superhelical-dependent transcription model (used in V1 and V2) is designed to capture the responsiveness of promoter kinetics. This sensitivity allows for distinctive behaviour to each parameterised promoter, highlighting the importance of accounting for sequence-dependent effects in this type of modelling and analysis.

3 TORCphysics modification levels

TORCphysics provides a flexible framework that enables the incorporation of new mechanisms describing the behaviour of proteins of interest. We define three levels of modification, each representing an increasing degree of complexity.

3.1 Level 1

In the context of CoSMoS, Level 1 modifications refer to the incorporation of new domain models that describe the binding, effect, or unbinding of biomacromolecules. In its current state, TORCphysics readily accounts for this type of modificationS, as it has been specifically designed for this purpose, with built-in functions that support user-defined extensions.

Examples of Level 1 modifications include:

- The simple binding and unbinding of repressors that physically block promoters.

- The effects of proteins that partially isolate the superhelical density, parameterised with a leak rate.
- The implementation of more complex topoisomerase kinetics involving multiple reaction steps.
- Rho-dependent termination.

The models presented in the manuscript can therefore be considered Level 1 modifications, as they extend TORCphysics by defining new mechanisms in the V0-V2 models. Modifications that require alterations in the platform model go beyond Level 1 and fall outside the scope of the present study.

3.2 Level 2

Level 2 modifications involve slight changes to the platform model and, in some cases, to the domain models as well, without substantially deviating from the overall architecture of TORCphysics.

Examples of Level 2 modifications include:

- Direct interactions between bound molecules: for example, transcription factors that, when bound, significantly enhance RNAP binding.
- DNA looping: for instance, the lacI repressor, which requires its binding to two operator sites and can probabilistically form a DNA loop, creating two topological domains.
- Structural transitions in superhelical DNA: including B–Z DNA transitions, strand separation, or cruciform formation, which could be parameterised using the SIST algorithm [2]. These transitions may absorb superhelical density, and their parameterisation may therefore be more complex.
- R-loop formation: R-loops can form when topoisomerase I fails to prevent the accumulation of negative supercoils. In principle, they could be modelled as an additional state of stalled RNAP complexes. Because R-loops promote recombination that lead to DNA damage, their formation may be fatal for the cell [3].

3.3 Level 3

Level 3 modifications involve substantial changes to the platform model and, potentially, to the domain models as well. These modifications may deviate considerably from the current TORCphysics framework but could greatly expand its capability to model more complex biological phenomena and interactions.

Examples of Level 3 modifications include:

- Environmental depletion: modelling variations in ATP availability over time, allowing simulations that span different bacterial growth phases.
- ATP/ADP ratio across dynamic growth phases.
- mRNA degradation and translation efficiency.
- Fluorescence signal modelling: integrating fluorescence-labelled reporter genes to enable direct comparison with single-molecule or live-cell imaging experiments.
- Writhe calculations: performing in-simulation estimation of DNA writhe to capture and model possible effects of DNA topology on protein dynamics.
- Superhelical density diffusion: modelling the propagation of supercoils at finer temporal resolutions (e.g., $\Delta t \ll 1$s).
- RNase H–mediated degradation of RNA: RNase H activity can be modelled as a mechanism to resolve R-loops. These enzymes would act as *environmentals* that bind to DNA–RNA hybrids, resolving them and potentially leading to transcription termination.

4 Parameterising Promoter Melting Energy with SIST

The one-step and three-step superhelical-dependent transcription models introduce the function U_{melt}, which represents the energy required to melt the promoter. This function is used to modulate the binding of RNAPs in the one-step superhelical-dependent transcription model and to modulate the open-complex formation in the three-step transcription model. Here, we describe the methodology used to parameterise the free energy function $U_{\text{melt}}(\sigma, s_p)$ as a function of the superhelical density σ and promoter sequence s_p within the genomic sequence s. More specifically, we implement the Superhelically Induced Duplex Destabilization (SIDD) model from the SIST algorithm [2] to calculate energy profiles.

Given a genomic sequence s, the SIST algorithm can calculate the free energy profiles $G(s_i, \sigma)$ that represent the relative stability of a given base-pair i to transition to strand separation at a given superhelical density σ. A high value of $G(s_i, \sigma)$ indicates a stable base-pair with low probability of strand separation, while a low value indicates a destabilization and a high probability of melting,

For the gene architecture experiments, we parameterise U_{melt} for the week, medium and strong promoters, by flanking each promoter s_p by 250 GC base-pairs on each side. This results in the genomic sequence $s = s_{GC} + s_p + s_{GC}$ with s_{GC} representing the 250 flanking GC sequences.

We then run SIST across a range of superhelical densities from $-0.2 \leq \sigma \leq 0.0$ to obtain the free energy profiles $G(s_i, \sigma)$. Figure S6 shows these free energy profiles for the three promoters.

To capture the promoter response to melting, we calculate the average energy $\bar{G}(s_p, \sigma)$ in the promoter region. This average energy typically follows a sigmoidal curve (see Figure S7). We then fit the following sigmoidal function:

$$U_{\text{melt}}(\sigma) = a + \frac{b}{1 + \exp\left(-\frac{\sigma - \sigma_m}{\epsilon_m}\right)} \tag{5}$$

Here, the parameters $a, b, \sigma_m, \epsilon_m$ are sequenced-dependent. The threshold (σ_m) and width (ϵ_m) are the parameters directly used in the rate equations k_{on} and k_{open} from the one-step and three-step superhelical-dependent transcription models, respectively. The fitted (U_{melt}) functions for each promoter are shown in Figure S7. Note that that U_{melt}, as well as parameters a and b, are in kcal/mol units, while σ_m and ϵ_m are dimensionless.

Since we use U_{melt} to modulate rates in the transcription models, we define a simplified, dimensionless form:

$$U'_{\text{melt}}(\sigma) = \frac{\mu}{1 + \exp\left(-\frac{\sigma - \sigma_m(s_p)}{\epsilon_m(s_p)}\right)} \tag{6}$$

Where we have introduced the dimensionless parameter μ and discarded a and b.

Given a rate k (as in the transcription models), it can be modulated through U'_{melt} by:

$$k(\sigma) = k \exp\left(-U'_{\text{melt}}(\sigma)\right) \tag{7}$$

We choose μ such that the rate k is reduced to 10% of its maximum activity when $\sigma >> \sigma_m$. In other words:

$$k(\sigma >> \sigma_m) = k \exp(-\mu) = (0.10)k \tag{8}$$

this results in $\mu \approx 2.3$.

This approach can be used not only to model melting response of any promoter, but can also be applied to modeling of strand separation susceptibility of any DNA region. It is important to note, however, that the resulting energy profiles and thus the parameterised U_{melt} (see Figures S6 and S7, respectively) are highly sensitive to the surrounding DNA sequence context. For instance, sequences with AT-rich regions may be susceptible to melting, potentially competing with strand-separation at the promoter itself, thereby considerably altering both energy profiles and promoter activity [4]. When the sequence context is unknown, we recommend using the flanking GC sequence method as an approximation. Nonetheless, further investigation is needed to fully understand how local sequence context influences DNA energetics and transcriptional regulation.

5 Marko's Elastic Model of Supercoiled DNA and RNAP velocity

In TORCphysics, the velocity of transcribing RNAPs is modelled using a torque-dependent form. To compute torque as a function of superhelical density, TORCphysics employs Marko's elastic model of supercoiled DNA [5]. For a DNA segment held under a constant stretching force f (in pN) and a superhelical density σ, the torque τ is given by:

$$\tau = \begin{cases} \frac{c_s}{\omega_0'}\sigma, & \text{if } |\sigma| < |\sigma_s| \\ \frac{\sqrt{2pg/(1-p/c_s)}}{\omega_0'}, & \text{if } |\sigma_s| < |\sigma| < |\sigma_p| \\ \frac{p}{\omega_0'}\sigma, & \text{if } |\sigma| > |\sigma_p| \end{cases} \tag{9}$$

The torque scales linearly with σ until $|\sigma| = |\sigma_s|$. In this regime the DNA exists purely in the form of twist. In the coexisting regime $|\sigma_s| < |\sigma| < |\sigma_p|$, where the DNA exists in the form of twist and writhe, the torque remains constant. Beyond $|\sigma| = |\sigma_p|$, supercoiling is stored entirely as writhe (plectonemic form).

The parameter $\omega_0' = \omega_0/.34$ (rad/nm) corresponds to the contour-length rate of rotation, with 0.34 nm being the contour length of a single base-pair in relaxed B-DNA form. The parameter $p = k_B T P \omega_0'^2$ (pN) (with P in length units) describes the twist stiffness of writhed DNA. The free energies per length (pN) for stretching (g) and twisting (c_s) are given by:

$$g = f - \sqrt{\frac{k_B T f}{A}} \tag{10}$$

$$c_s = c\left(1 - \frac{C}{4A}\sqrt{\frac{k_B T}{Af}}\right) \tag{11}$$

where A (nm) is the bending persistence length, C (nm) the twist persistence length, and $c = k_B T C \omega_0'^2$ (pN) is the twist stiffness of DNA.

The critical values σ_s and σ_p, which define the boundaries of the three supercoiled regimes, are calculated as:

$$|\sigma_s| = \frac{1}{c_s}\sqrt{\frac{2pg}{1 - p/c_s}} \tag{12}$$

$$|\sigma_p| = \frac{1}{p}\sqrt{\frac{2pg}{1 - p/c_s}} \tag{13}$$

This elastic model is valid for relatively low stretching forces (a few pN). In TORCphysics, we assume all stretching forces are low and constant. The specific parameter

values used in this model are listed in Table S1. Figure S1a) shows the torque as a function of the superhelical density using this parameterisation.

The torque experienced by a transcribing RNAP is modelled as the balance of torques:

$$\tau = \tau_{\text{front}} - \tau_{\text{behind}} \tag{14}$$

where, for an RNAP elongating from left to right, the downstream torque corresponds to τ_{front} and the upstream torque to τ_{behind}. The RNAP velocity is then described by a sigmoidal function of the net torque:

$$v = \frac{dx}{dt} = \frac{v_0}{1 + \exp\left(\kappa(\tau(\sigma) - \tau_0)\right)} \tag{15}$$

In practice, we use the magnitude of the torque, $\tau_{\text{mag}} = |\tau|$, to constrain the velocity in cases where the net torque becomes large (see Figure S1b). It is important to note that TORCphysics only considers supercoiling in the form of twist, whereas Marko's model accounts for writhed states. At typical physiological superhelical densities ($\sigma \in [-0.06, -0.04]$), DNA tends to exist in the coexistence regime according to Marko's model with parameters from table S1, where σ_s is relatively low and σ_p corresponds to highly supercoiled states. We consider this model suitable for TORCphysics as this leads to an approximately constant torque for most scenarios.

In cases of hypernegatively supercoiled DNA, which is assumed to adopt a fully plectonemic structure, RNAPs stall immediately due to the sigmoidal velocity. Figure S1b illustrates this behaviour for two deterministic trajectories in a small topological domain (1520 base-pairs in size), where RNAPs stall rapidly as negative supercoils accumulate upstream and reach the plectonemic regime. Although an RNAP could in principle continue elongating when upstream and downstream superhelical densities are both large and equal, such conditions are highly unlikely in vivo because of the steady supercoiling levels maintained by topoisomerases in bacteria. In addition, due to the nature of the twin supercoils generated by elongating RNAPs, the enzyme would soon stall as these supercoils keep accumulating. A constraint could be introduced in the velocity formulation to exclude these rare cases, however, we intentionally retain them here to highlight the velocity–superhelicity landscape governing RNAP dynamics (see Figure S1b). This analysis highlights that transcription dynamics is strongly impacted by torque and supercoiling, and that efficient removal of excess supercoils is essential for a smooth elongation, as only a narrow region of the velocity–superhelicity space permits RNAP progression.

6 Elastic function and spacer model equivalency

The three-step superhelical-dependant transcription model used in TORCphysics incorporates an elastic function G_{elastic} to emulate the closed-complex formation. A previous model proposed by Forquet et al. [6] predicts the relative activation of promoters based on their spacer length and orientation as a function of DNA supercoiling. Here, we demonstrate that both models are equivalent.

The spacer length model is defined as:

$$G_{\text{spacer}}(\sigma, n) = \frac{n}{2} k_\theta \left(\frac{\theta_p}{n} - \alpha_0(1+\sigma) \right)^2 \tag{16}$$

where n is the spacer length, $k_\theta = 71.4\,\text{kBrad}^{-2}$ is the DNA sequence twist stiffness, $\alpha_0 = 34$ is the average twist angle, and θ_p is the optimal twist angle between the -35 and -10 promoter regions.

The optimal twist angle, θ_p, can be related to the optimal superhelical density $\sigma_0 = -0.06$ as follows:

$$\theta_p = n\alpha_0(1+\sigma_0) \tag{17}$$

Thus, the spacer length model becomes:

$$G_{\text{spacer}}(\sigma, n) = n k_\theta \alpha_0^2 \frac{(\sigma - \sigma_0)^2}{2} \tag{18}$$

We associate the TORCphysics elastic function with the spacer length model as follows:

$$G_{\text{elastic}}(\sigma, \sigma_e, \epsilon_e) = G_{\text{spacer}}(\sigma, n) \tag{19}$$

$$\frac{(\sigma - \sigma_e)^2}{2\epsilon_e^2} = n k_\theta \alpha_0^2 \frac{(\sigma - \sigma_0)^2}{2} \tag{20}$$

With this, the elastic model takes the form of the spacer length model with $\sigma_e = \sigma_0 = -0.06$ and $\epsilon_e = \frac{1}{\sqrt{n k_\theta \alpha_0^2}}$.

In both forms, G_{elastic} and G_{spacer} are parameterised by the spacer length (structure) and the twist stiffness (stiffness), and unlike U_{melt}, the twist stiffness calculation of promoters is more demanding since it can be inferred from all-atom molecular dynamics simulations [7] or coarse-grained simulations [8], as well as from sequence-dependent stiffness models [9, 10], which are still under development. In this work, we determine its values indirectly by testing multiple parameterisations using random search (see the genetic architecture calibration section in the main text).

7 Calculating Global Superhelical Density from Experimental Kinetic Parameters of DNA Topoisomerases

In this section, we present the methodology for constructing the reference curves for the change in DNA superhelical density, used to calibrate the models that simulate the

stochastic activity of topoisomerases. In general, the process consists integrating reaction curves using the kinetic parameters measured by Wang et al. [11], and then inferring the global superhelical density.

7.1 Kinetic curves integration

Simple enzyme kinetics can be described by the Michaelis-Menten equation:

$$v = v_{\max} \frac{S}{K_M + S} \tag{21}$$

$$k_{\text{cat}} = \frac{v_{\max}}{E} \tag{22}$$

$$v = \frac{dP}{dt} = -\frac{dS}{dt} \tag{23}$$

Here v represents the velocity or rate of product formation P, S denotes the substrate concentration, E the enzyme concentration, P the product concentration, $v_{\max}$ the maximum velocity, K_M the Michaelis constant, and k_{cat} the catalytic rate constant. This equation describes how reaction rates vary with changes in enzyme and substrate concentration. The general reaction scheme of an enzyme-catalyzed reaction is illustrated in Figure S3a).

Given an initial substrate concentration and a set of kinetic parameters ($v_{\max}, K_M, k_{\text{cat}}$), we can numerically integrate the Michaelis-Menten equation using the Euler method:

$$P_{i+1} = P_i + v_i \Delta t \tag{24}$$

$$S_{i+1} = S_i - v_i \Delta t \tag{25}$$

By iterating and updating these simple set of equations, we can generate the resulting kinetic curves.

7.2 Topoisomerase I (topo I) kinetics

The reaction scheme for the relaxation reaction catalyzed by *E. coli* DNA topoisomerase I (topo I), as proposed in [11], is shown in Figure S3b. According to this scheme and following the Michaelis-Menten equation21, the substrate would be the concentration of supercoiled DNA, to which topo I binds, resulting in the production of relaxed DNA. In their kinetic study, an initial plasmid concentration of 0.75nM supercoiled DNA and a constant topo I concentration of 17.0 nM were utilized. By incorporating these concentrations and the kinetic parameters derived in their study (see Table S2), we were able to numerically integrate and obtain the relaxation curves shown in Figure S4, where topo I relaxes supercoiled DNA, transitioning the total plasmid concentration from supercoiled DNA to relaxed DNA.

We can associate the reaction curves to the change in superhelical density in the plasmid by assuming a linear relationship between the concentration of relaxed DNA and the superhelical density:

$$\sigma(t) = a + R(t)b \tag{26}$$

where R is the concentration of relaxed DNA (product) as a function of time. When the superhelical density is zero, the concentration of relaxed DNA corresponds to the total plasmid concentration of .75nM. We then assume that at the superhelical density of -0.11 suggested as the maximum superhelical density induced by DNA gyrase according to previous observations [12], the total plasmid concentration shifts to supercoiled DNA, hence $R = 0$. Any state in between corresponds to a mixture of superhelical and relaxed DNA. Given these conditions, the previous relationship takes the form:

$$\sigma(t) = -0.11 + 0.11\frac{R(t)}{0.75\text{nM}} \tag{27}$$

Figure S4 shows the resulting relaxation curve in terms of the concentration of relaxed DNA (panel a), the concentration of supercoiled DNA (panel b), and the corresponding superhelical density calculated using equation 7.2 for the where topo I interacts solely with supercoiled DNA (red curve). For this case, we set the initial superhelical density to $\sigma = -0.11$, which corresponds to a fully supercoiled DNA state ($R = 0$).

7.3 Gyrase kinetics

Similar to topo I, the reaction scheme for the supercoiling-induced reaction catalyzed by *E. coli* DNA Gyrase is shown in Figure S3c, as proposed by Wang et al. [11]. According to this kinetic pathway, gyrase binds the relaxed DNA substrate, producing supercoiled DNA, and releasing the gyrase enzyme. Although *E. coli* gyrase has two substrates, relaxed DNA and ATP, we do not consider the gyrase-ATP complex formation in this study. Instead, we assume that gyrase alone interacts with relaxed DNA. However, it is important to note that the kinetic parameters obtained in [11] and utilized in this study (see table S2, maintained the ATP concentration at 1.75 mM during their assay.

By implementing and integrating the Michaelis-Menten equation (see equations 21-23, and utilizing the gyrase kinetic parameters obtained in [11] (see table S2), we are able to generate the supercoiled-induced curves for DNA Gyrase interacting with relaxed DNA (see Figure S4b, represented by the blue curve). We start from a relaxed DNA state corresponding to $R = 0.75$nM and $\sigma = 0$. By applying the relationship between the superhelical density and the concentration of relaxed DNA, we can infer the superhelical density using equation 7.2. Figure S4c (blue curve) illustrates the resulting superhelical density for DNA gyrase interacting with relaxed DNA.

7.4 Both topo I and gyrase kinetic curve

In addition to the scenarios involving DNA gyrase or topo I interacting independently with DNA, we simulate two cases where both enzymes interact simultaneously with the DNA by integrating the Michaelis-Menten equation 21. Here, topo I acts on the supercoiled DNA substrate (C)) and produces relaxed DNA (R), while gyrase acts on relaxed DNA substrate and produces supercoiled DNA (see Figure S3b-c). The corresponding set of kinetic equations is as follows:

$$C_{i+1} = C_i + (v_{\text{gyrase}} - v_{\text{topoI}})\Delta t \quad (28)$$

$$R_{i+1} = R_i - (v_{\text{gyrase}} - v_{\text{topoI}})\Delta t \quad (29)$$

The rates v_{gyrase} and v_{topoI} are determined by substituing the corresponding gyrase and topo I kinetic parameters and substrates (relaxed DNA and supercoiled DNA) into equation 21. We initiate the kinetic simulations at the superhelical level of 0, corresponding to a relaxed state with concentration of relaxed DNA of 0.75nM and 0 for supercoiled DNA, and at the superhelical level of -0.11, corresponding to a concentration of supercoiled DNA of 0.75nM and 0 for relaxed DNA (refer to Figure S4a-b). Figure S4c displays the corresponding superhelical density for both cases (green and purple curves), where both conditions tend to the plateau with a value of approximately -0.046, approximating physiological values of in vivo superhelical density in *E. coli* [13].

8 Upstream vs Downstream barrier distance variations

The experiments reported by Boulas et al. [1] indicate that increasing the distance to the downstream barrier does not affect gene expression for promoters of different strengths, whereas varying the position of the upstream barrier has a larger effect. To test this within TORCphysics, we ran the gene architecture experiment for model V2 and varied the position of one barrier while keeping the other one fixed. The barrier was positioned at 320 bp from the promoter when the downstream barrier was varied and at 320 bp from the terminator when the upstream barrier was varied. We launched the same 3900 random promoter parameterisations for both barrier variations. Each parameter set was composed of: $k_{\text{on}}, k_{\text{off}}, k_{\text{closed}}, k_{\text{open}}, k_{\text{ini}}, \sigma_e$ and ϵ_e. For each parameter set, 60 simulations were performed per distance, where the estimated susceptibility was computed using the barrier distance of approximately 250 bp as reference. The resulting distribution of susceptibilities grouped by barrier distance are shown in Figure S26.

Our results are broadly consistent with Boulas et al. [1], where we found similar ranges of susceptibilities as a function of distance to the barrier (see Figure S26). Within TORCphysics, the susceptibility values reach up to 1.3 when the upstream barrier is varied, whereas varying the downstream barrier can reduce the susceptibility to 0.7. The mean susceptibility at large barrier distances of 3 kb is approximately 1.1 for upstream barrier variation and 0.9 for downstream barrier variation. Boulas et al. [1] reported

experimental susceptibilities of around 1.2 for the upstream and in the range of 0.9 to 1.1 for the downstream at barrier distances of approximately 3 kb. The distributions observed within TORCphysics here cover those ranges.

9 Supplementary Figures

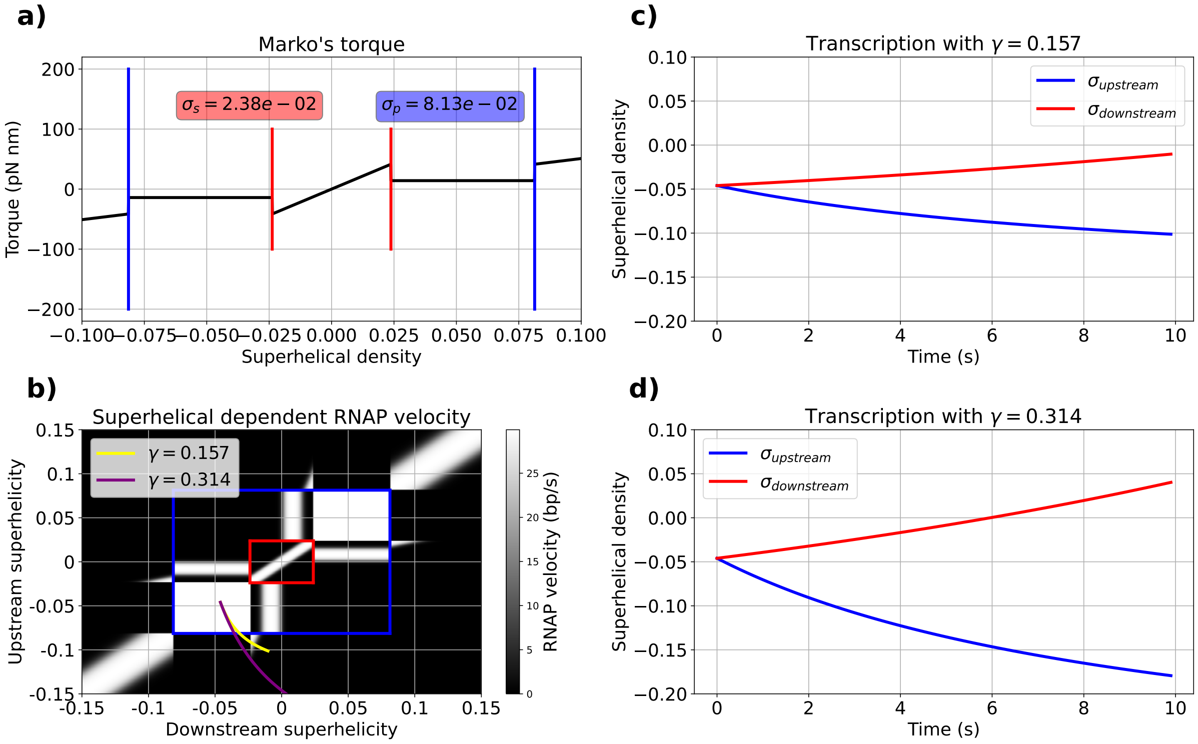

Figure S1: Torque handling and RNAP velocity in TORCphysics. (a) Torque as a function of superhelical density according to Marko's model (see Supplementary Section 5). The threshold σ_s marks the region where supercoiled DNA purely exists in the form of twist (red), whereas beyond the threshold σ_p DNA adopts a plectonemic configuration (blue). Between these two thresholds, DNA is assumed to exist in a mixed state of twist and plectonemes. (b) RNAP velocity as a function of upstream and downstream superhelicity. Red lines correspond to the σ_s threshold and blue lines to the σ_p threshold. The yellow and purple trajectories represent the first ten seconds of transcription for RNAP twist injection ratios of $\gamma = 0.157$ and $\gamma = 0.314$ from (c) and (d), respectively. (c–d) Downstream (red) and upstream (blue) superhelical densities from deterministic simulations with uniform RNAP motion (no stalling) for $\gamma = 0.157$ (c) and $\gamma = 0.314$ (d). These simulations are equivalent to the genetic architecture experiments with a gene of 900 base-pairs long with an upstream barrier at 300 bp from the start site (shortest upstream distance), and a downstream distance at 320 bp away from the termination site. Shaded grey areas indicate time intervals during which RNAP would pause due to torque if the stalling mechanism were included.

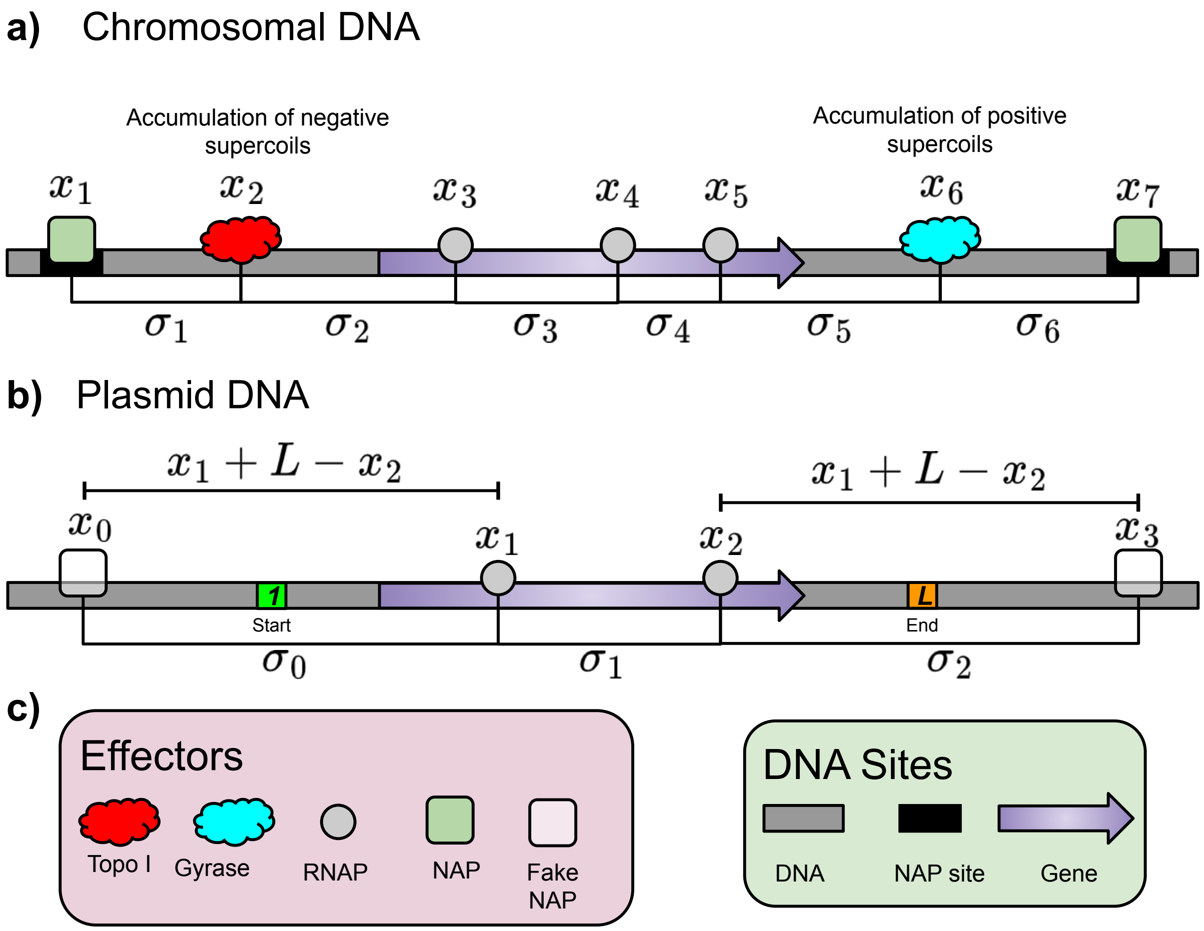

Figure S2: Representation of TORCphysics genetic circuit configurations. a) Chromosomal DNA is represented as a linear sequence flanked by boundary topological barriers that isolate supercoils from external regions. These boundary barriers are assumed to be NAPs and remain bound throughout the simulation. In chromosomal DNA, negative supercoils accumulate upstream of transcribing genes, while positive supercoils accumulate downstream, consistent with the twin-domain supercoiling model [14]. DNA topoisomerase I (topo I) preferentially binds negatively supercoiled DNA, while gyrase binds positively supercoiled DNA. In this example, the number of bound effectors is $N = 7$. b) Plasmid DNA is represented as circular DNA. The beginning of the sequence (base-pair 1) is highlighted with a green square, while the end of the sequence (base-pair L) is marked with an orange square. Since TORCphysics is a one-dimensional model, circular sequences are mimicked by adding artificial (fake) NAPs that act as topological boundaries. Boundary x_0 mimics x_2, while boundary x_3 mimics x_1. Therefore, the distance from the left boundary x_0 to the first effector x_1 is equivalent to the distance from x_2 to x_1, i.e., $x_1 + L - x_2$, where L is the length of the DNA sequence. In plasmid DNA, supercoils generated during transcription do not accumulate, as they propagate to both sides of the gene and cancel each other out. In TORCphysics, this cancellation arises naturally from the model design, which simulates the generation and propagation of transcription-induced supercoils. In this illustration, the number of bound effectors is $N = 2$. c) Description of effectors and DNA sites used in these illustrations. Environmentals are omitted for simplicity.

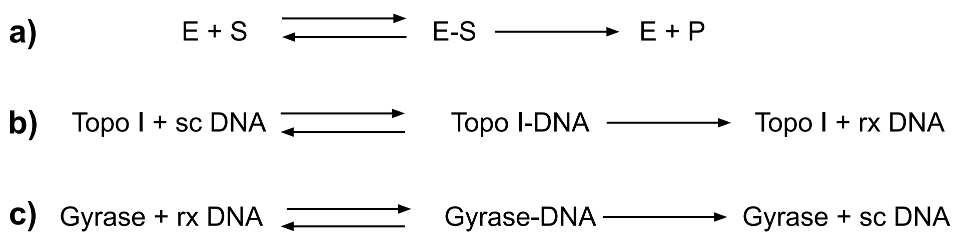

Figure S3: Topoisomerases kinetic reaction scheme in *E. coli* as proposed by Wang et. al. [11]. a) General kinetic pathway described by the Michaelis-Menten equation. Enzyme E binds substrate S to form the complex E-S, followed by product P production and enzyme E release. b) Kinetic pathway of topoisomerase I acting on supercoiled DNA. Topoisomerase I (topo I) binds supercoiled DNA substrate (sc DNA) to form DNA-topoI complex, resulting in relaxed DNA production and topo I release. c) Reaction scheme of DNA gyrase acting on relaxed DNA. Gyrase binds relaxed DNA to form DNA-Gyrase complex, leading to supercoiled DNA production. ATP concentration remains constant in this reaction scheme.

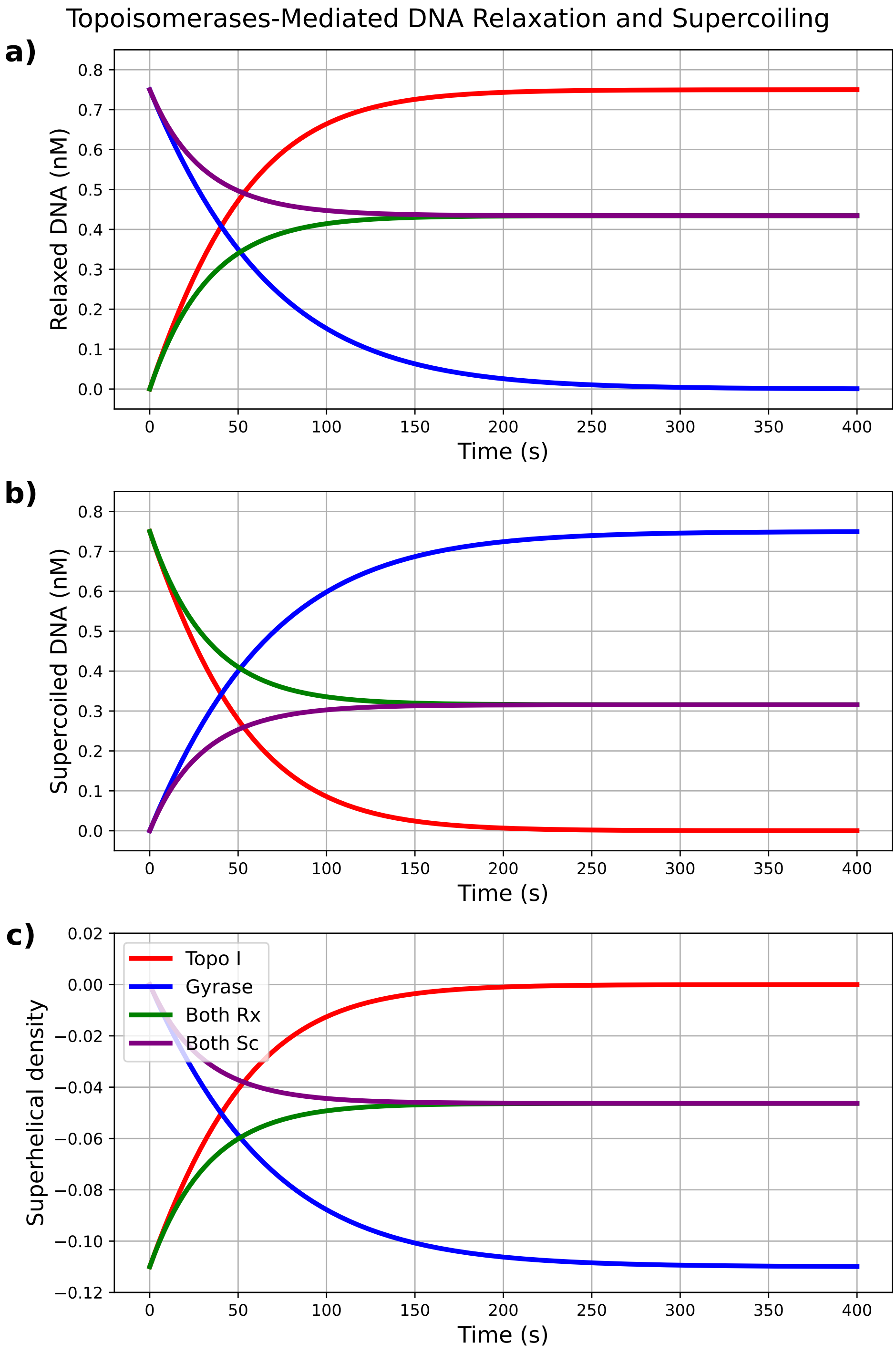

Figure S4: Time courses of relaxation (a) and supercoiling (b) reactions catalyzed by *E. coli* DNA topoisomerase I (topo I) in red, DNA gyrase in blue, both enzymes acting simultaneously on DNA starting from a supercoiled state in green, and starting from a relaxed state in purple, obtained through the integration of the Michaelis-Menten equation. Panel (c) illustrates the corresponding superhelical density determined by a linear relationship with relaxed DNA, as shown in equation 7.2, for the three scenarios. The kinetic parameters utilized are taken from [11] and shown in table S2.

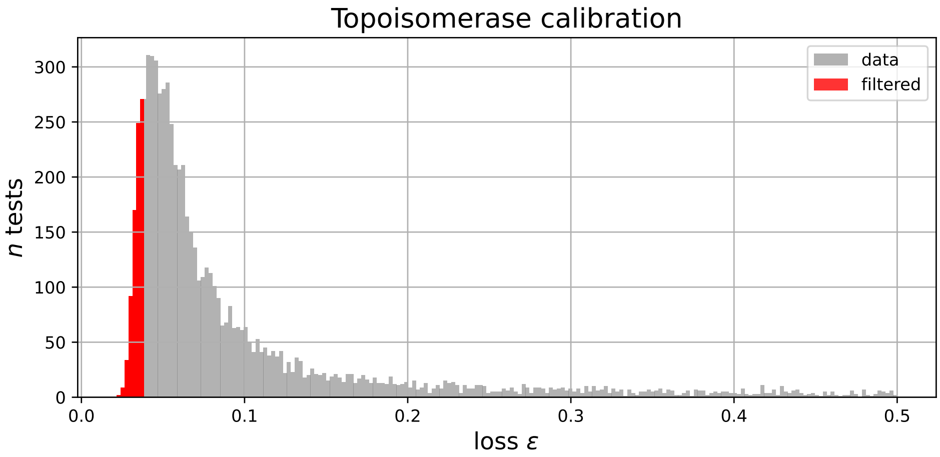

Figure S5: Distribution of losses ϵ resulting from the calibration of the stochastic activity of topoisomerases. Losses are calculated according the objective function used to evaluate the model. Red bins indicate the top 5% of best parameter sets out of 8,000 random tests.

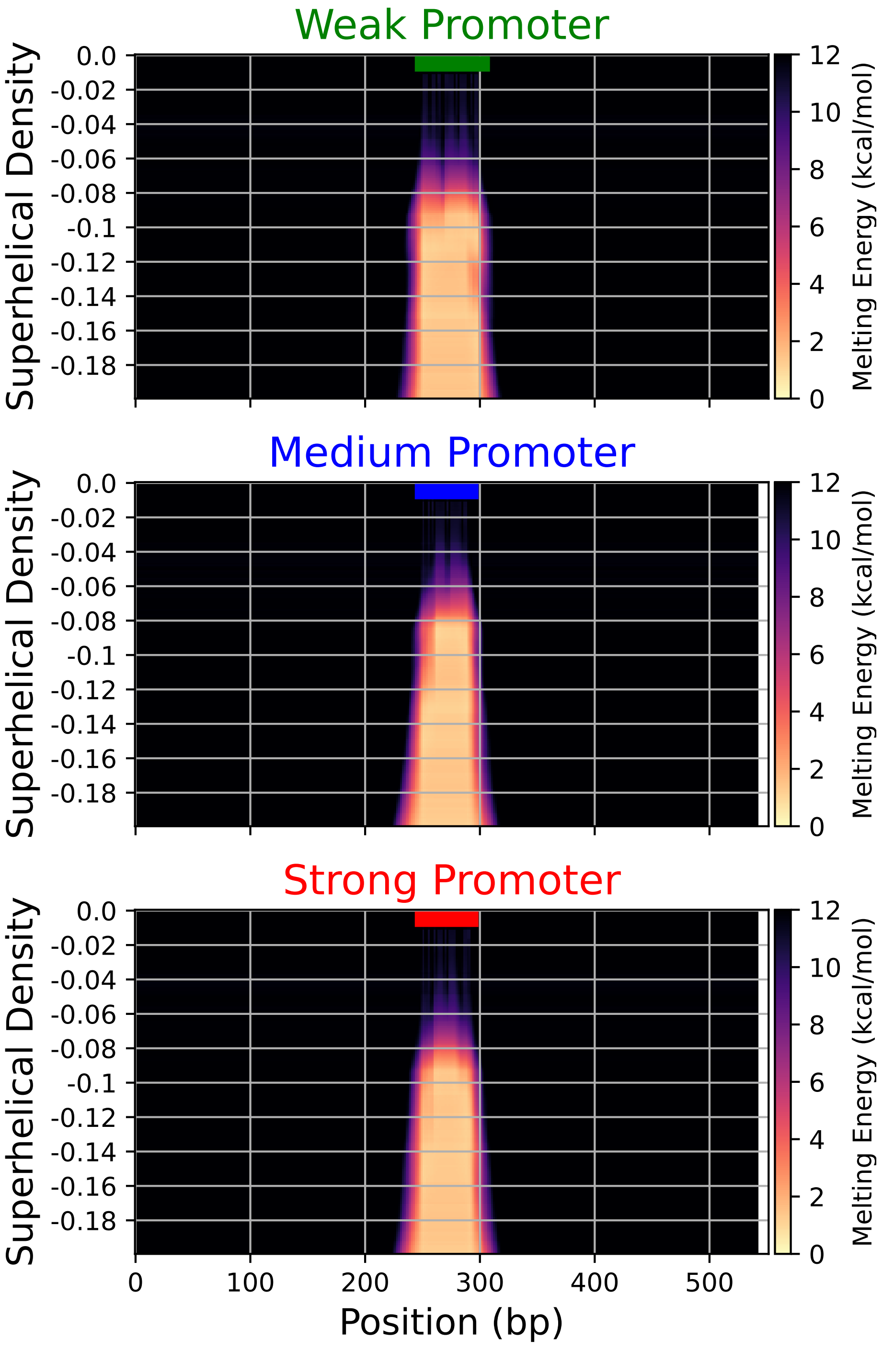

Figure S6: Melting energy profiles ($G(s, \sigma)$) for the weak, medium and strong promoters flanked by 250 GC sequences on each side. The profiles were calculated using SIST. The colored lines at the top of each subplot indicate the promoter positions: green for the weak promoter, blue for the medium promoter, and red for the strong promoter.

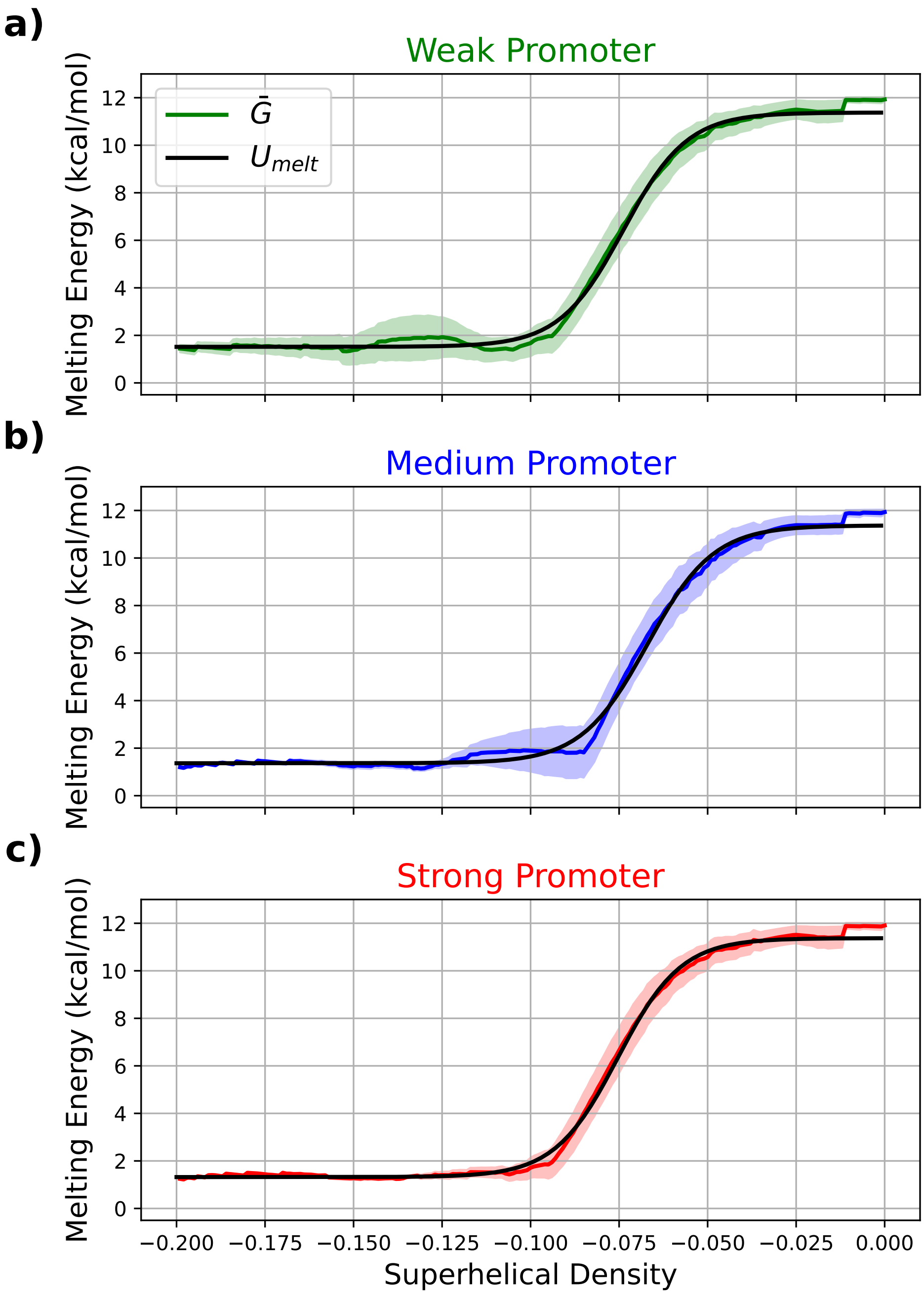

Figure S7: Promoter response U_{melt} obtained by fitting equation 5 to the averaged melting energy $\bar{G}$, for the weak (a, green), medium (b, blue) and strong (c, red) promoters. The promoter sequences were flanked by 250 GC base-pairs on each side for this case. However, in realistic scenarios, the response depends on sequence context. Energies are averaged around the -10 region, where small standard deviations indicate that the energy required for strand separation do not vary greatly in these regions.

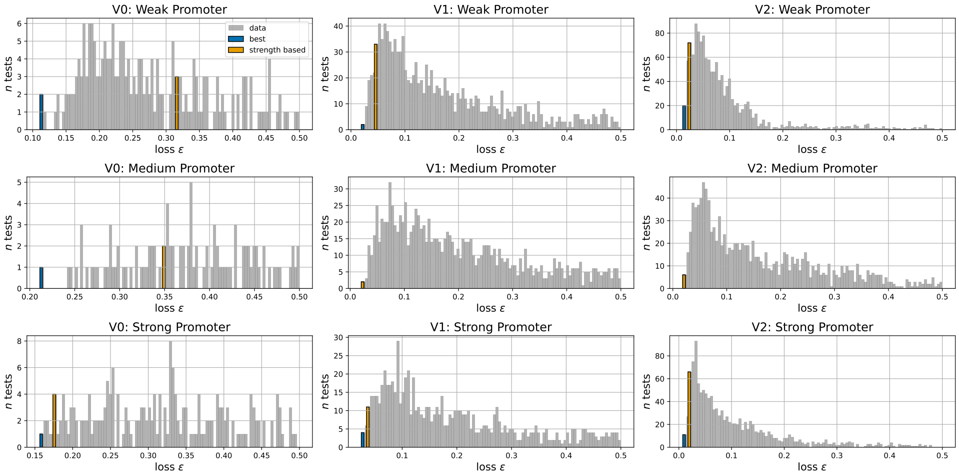

Figure S8: Distribution of losses resulted from the gene architecture calibration process for the three models V0, V1, and V2, across the weak, medium, and strong promoters. The parameter set that best fits the susceptibility is within the blue bin, while the set that also best reproduces the relative expression rates from experimental data is within the yellow bin. A total of 300 random tests were run for V0, and 1,500 for both V1 and V2.

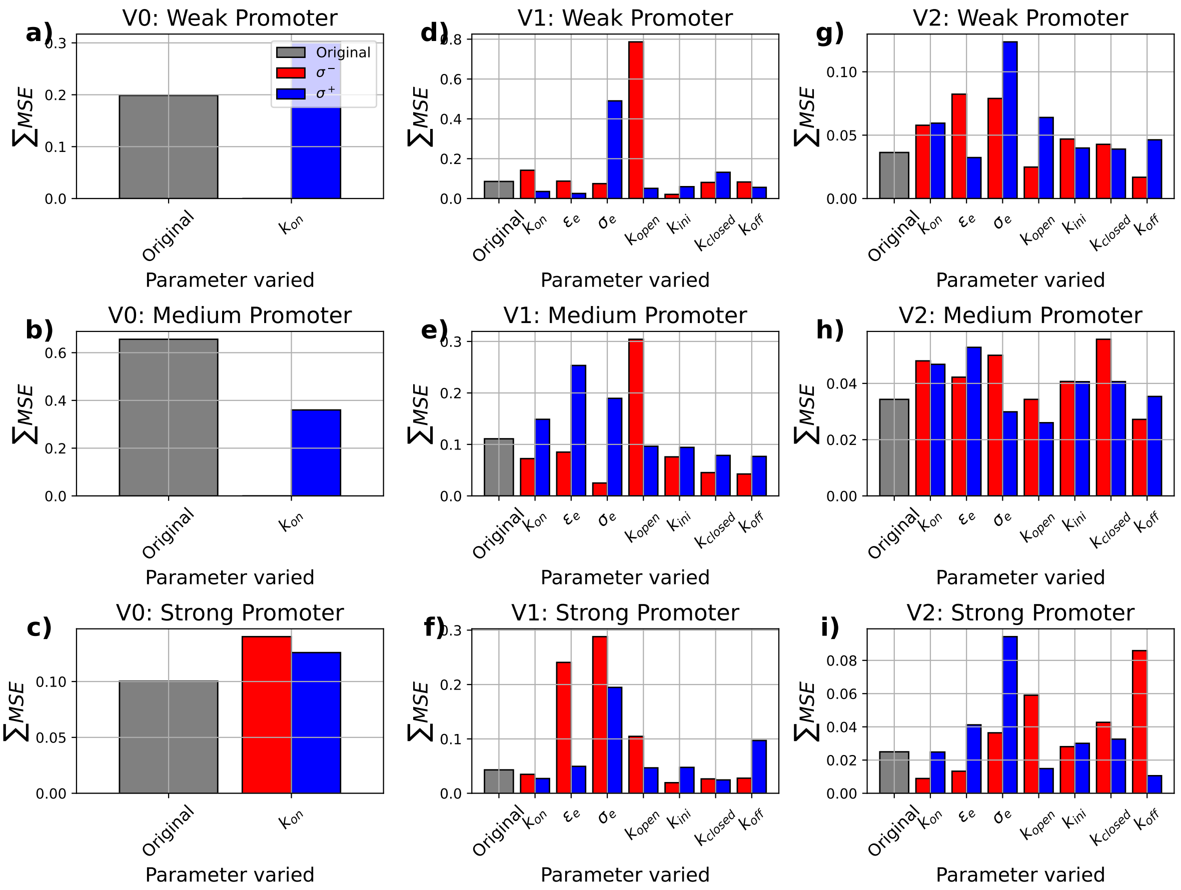

Figure S9: Sensitivity analysis of promoter dynamics models (V0–V2) in genetic architecture experiments, shown in terms of the sum of squared errors ($\sum MSE$). The first column (a–c) presents results for the V0 model, which implements the one-step transcription model for the three promoters. The middle column (d–f) shows the V1 model, which uses the three-step transcription framework, and the final column (g–i) corresponds to the V2 model, which incorporates the three-step transcription model coupled with the RNAP tracking by topoisomerase I mechanism. In this analysis, the original parameterisation was selected to match the experimental relative expression rates and does not necessarily correspond to the best-fitting parameterisation with the lowest error.

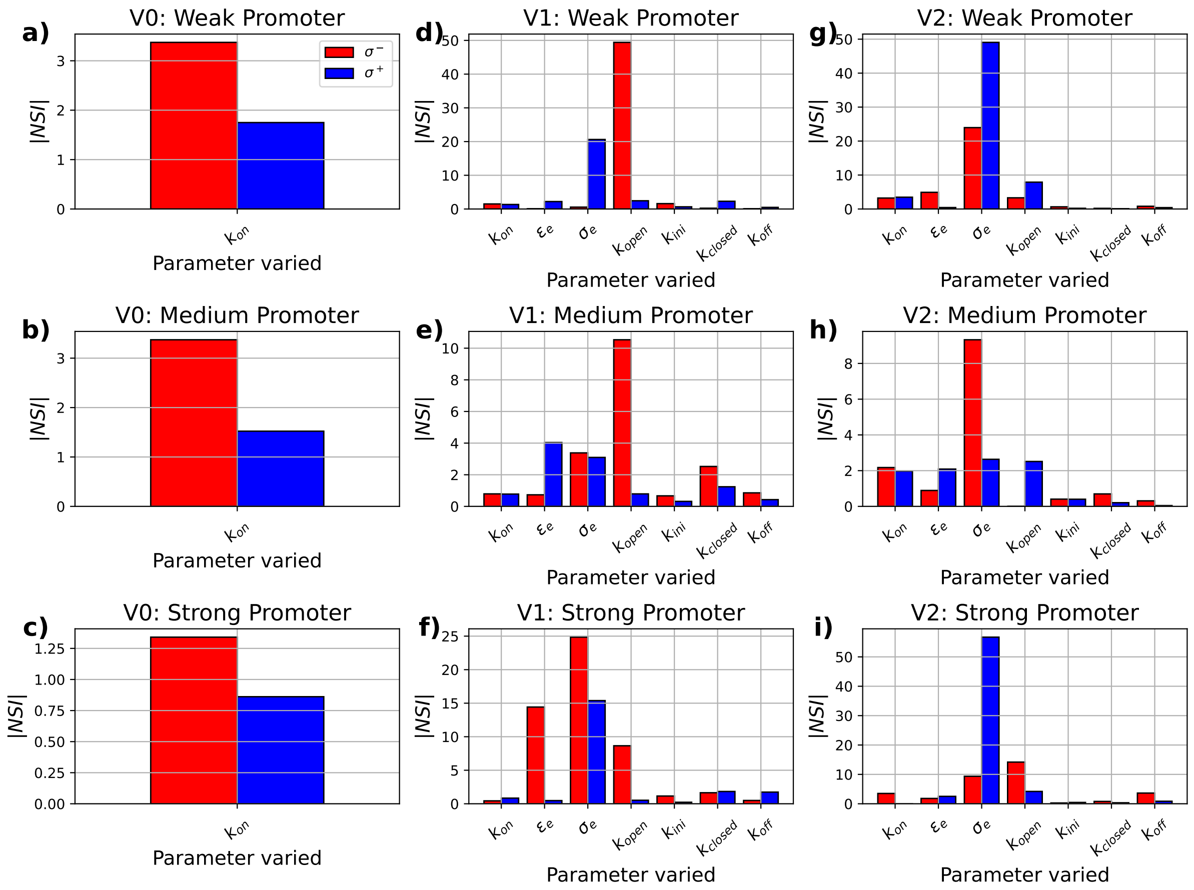

Figure S10: Sensitivity analysis of promoter dynamics models (V0–V2) in genetic architecture experiments, shown in terms of the normalised sensitivity index (NSI). The first column (a–c) presents results for the V0 model, which implements the one-step transcription model for the three promoters. The middle column (d–f) shows the V1 model, which uses the three-step transcription framework, and the final column (g–i) corresponds to the V2 model, which incorporates the three-step transcription model coupled with the RNAP tracking by topoisomerase I mechanism.

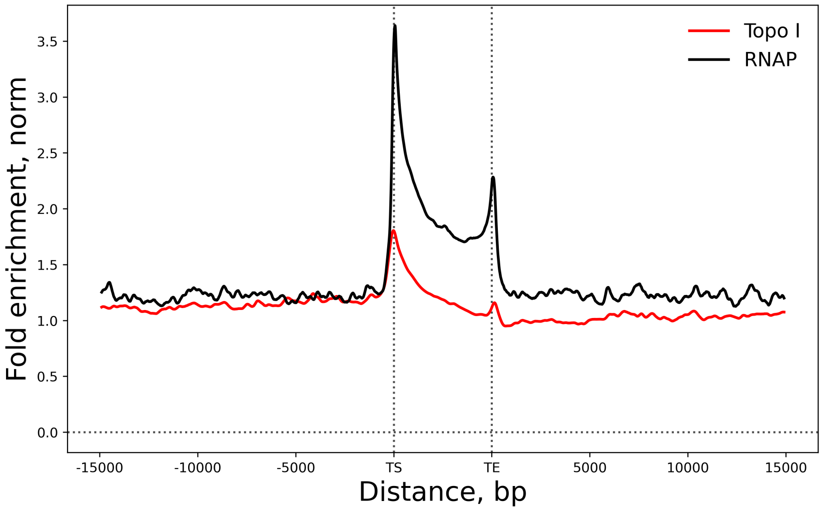

Figure S11: Topoisomerase I fold enrichment and normalized RNAP signal, obtained from Chip-Seq data used in Sutormin et al. [15]. Rather than directly using this data in TORCphysics, the model aims to reproduce equivalent averaged fold enrichment of topoisomeorase I within the TU ($\mathcal{F}_{\text{exp}} = 1.24$), the correlation coefficient between the both curves ($\rho_{\text{exp}} = 0.94$). Transcription unit is defined as the region between the transcription start site (TS) and transcription termination site (TE). TORCphysics simulates an equivalent system composed by a topological domain with a single gene (TU) at the middle.

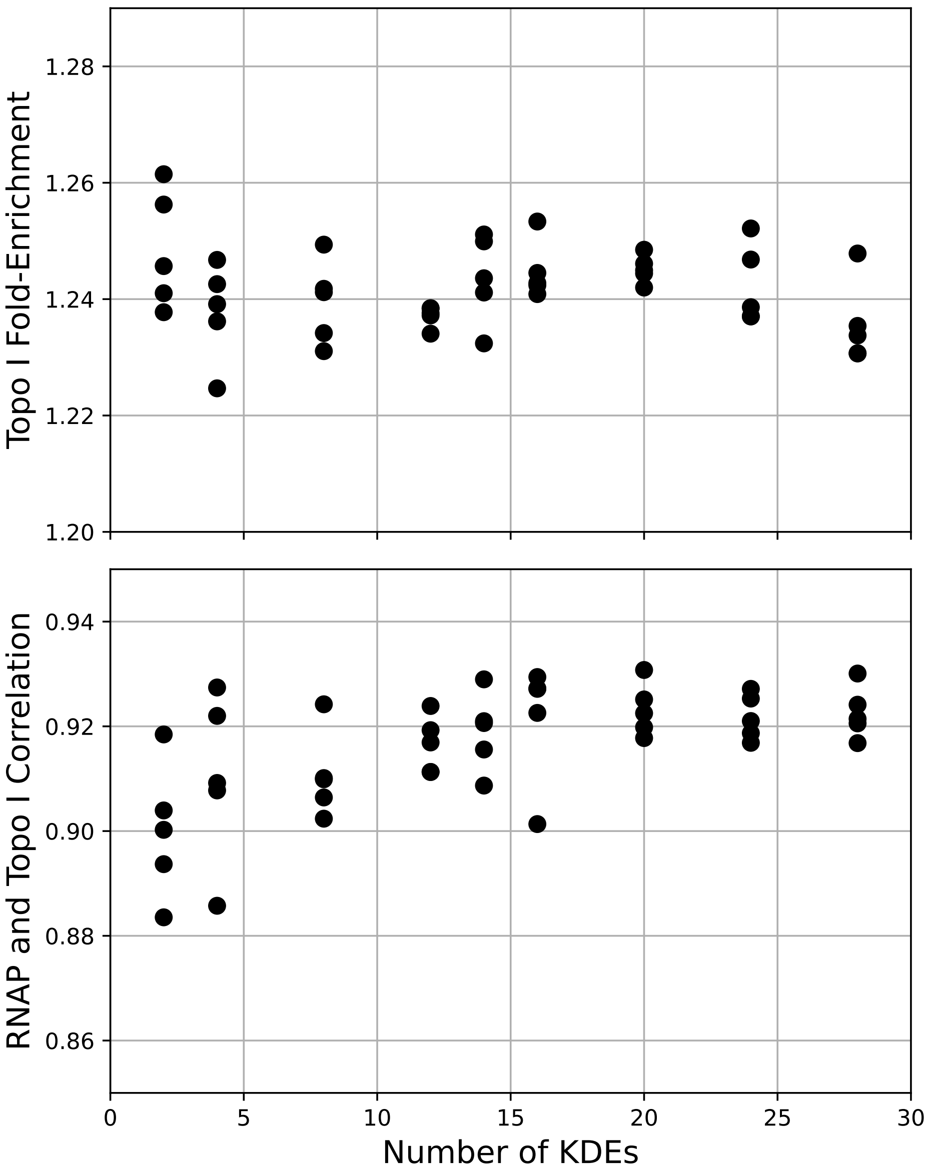

Figure S12: Test for the number of Kernel Density Estimation (KDE) samples used to stabilise the Topoisomerase I fold enrichment and the correlation between RNAP and Topoisomerase I in the RNAP tracking by topoisomerase I experiment. Each enzyme's KDE is obtained from histograms built using eight independent 3000 second simulations. Topoisomerase I fold enrichment (FE) is computed as the ratio of KDEs from simulations with active transcription (RNAP present) to reference KDEs from simulations without transcription (no RNAPs). The average FE is then calculated by averaging the KDEs across the transcription unit (TU), followed by computing a mean fold enrichment $\mathcal{F}$ as the mean of all averaged FEs. Finally, mean KDEs for RNAP and Topoisomerase I are used to compute the correlation coefficient ρ within the TU.

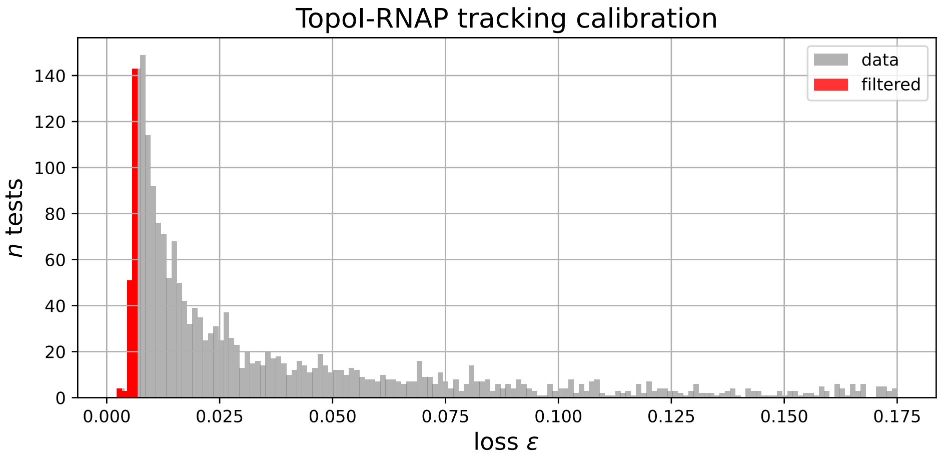

Figure S13: Loss distribution from the calibration of RNAP tracking by topoisomerase I. Loss values were computed using the objective function designed to evaluate how well the model mimics experimental behaviour. The top 5% of best parameter sets, out of 3,000 random trials, are highlighted in red.

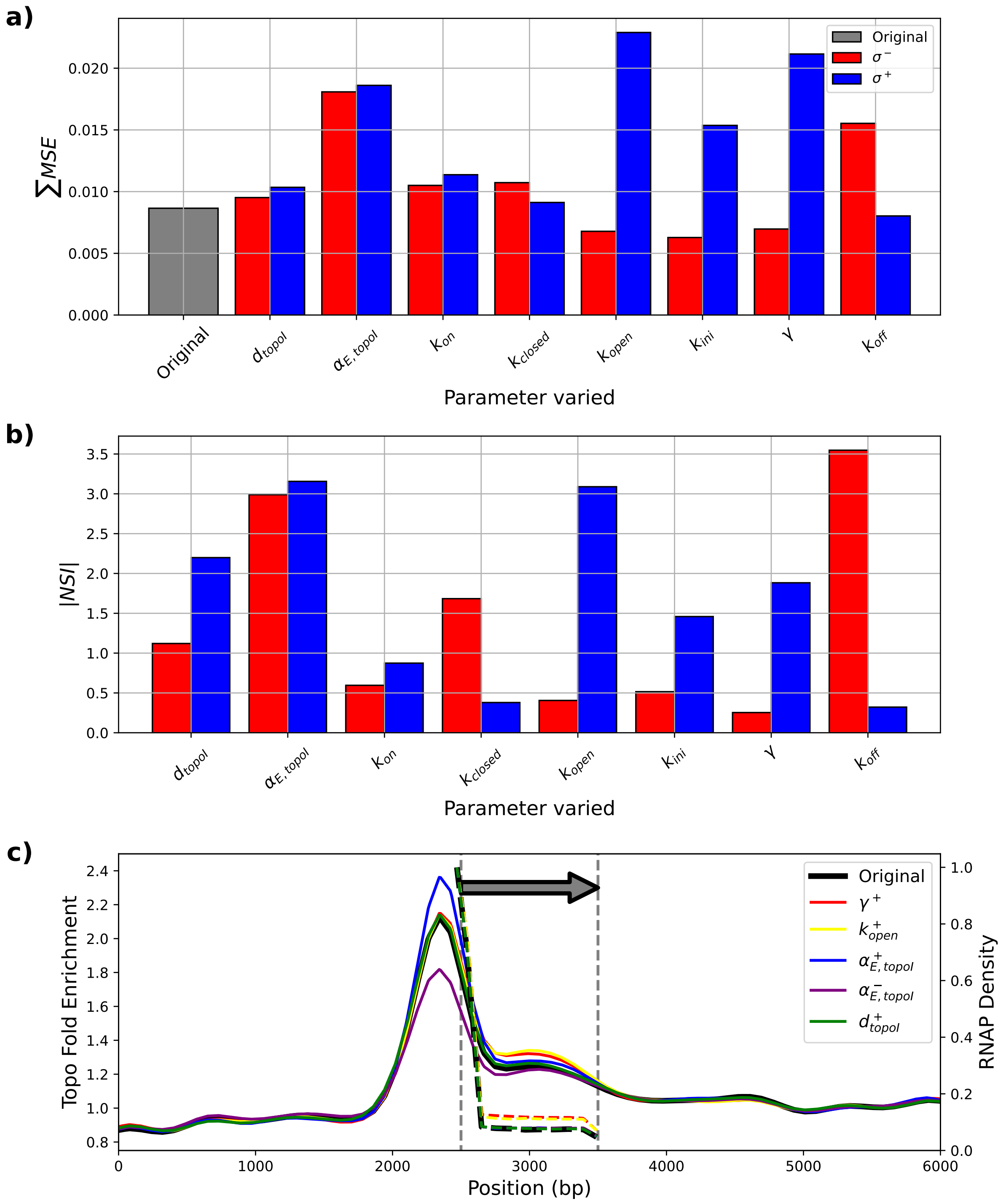

Figure S14: Sensitivity analysis of the RNAP tracking by topoisomerase I model. The sum of squared errors ($\sum MSE$) for each parameter variation is shown in (a), and the corresponding normalised sensitivity index (NSI) in (b). Simulation results for the original (averaged) parameterisation and key individual variations are shown in panel (c). The original results are shown in black, while key variations include increased RNAP twist rate (γ^+, red), increased open-complex formation rate (k^+_{open}, yellow), increased ($\alpha^+_{E,\mathrm{topoI}}$, blue) and decreased ($\alpha^-_{E,\mathrm{topoI}}$, purple) topoisomerase I binding enhancer, and increased enhancer effective distance (d^+_{topoI}, green). Solid lines represent topoisomerase I fold enrichment (left axis), while dashed lines correspond to RNAP position density (right axis). The grey arrow and dashed lines indicate the transcription region in the simulated system.

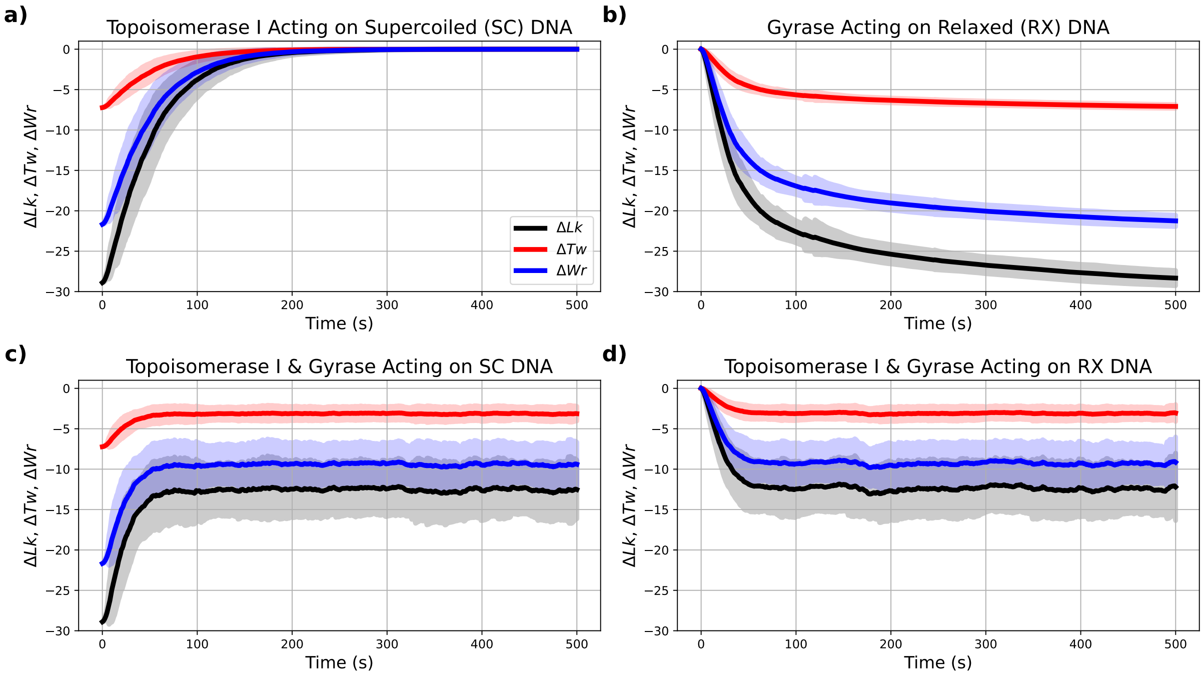

Figure S15: DNA topology calculations in terms of the linking difference ΔLk (black), change in twist ΔTw (red) and writhe ΔWr (blue) from simulations of the Modeling the stochastic activity of DNA topoisomerase I and DNA gyrase on supercoiled DNA experiment, calibrated with kinetic data from Wang et al. [11]. Twist and writhe were derived from the global superhelical density reported in the main text, using a twist-writhe ratio of 1:3 [16, 17]. Panels show: a) topoisomerase I acting on supercoiled DNA, b) gyrase acting on relaxed DNA, c) topoisomerase I and gyrase acting on supercoiled DNA, and d) topoisomerase I and gyrase acting on relaxed DNA.

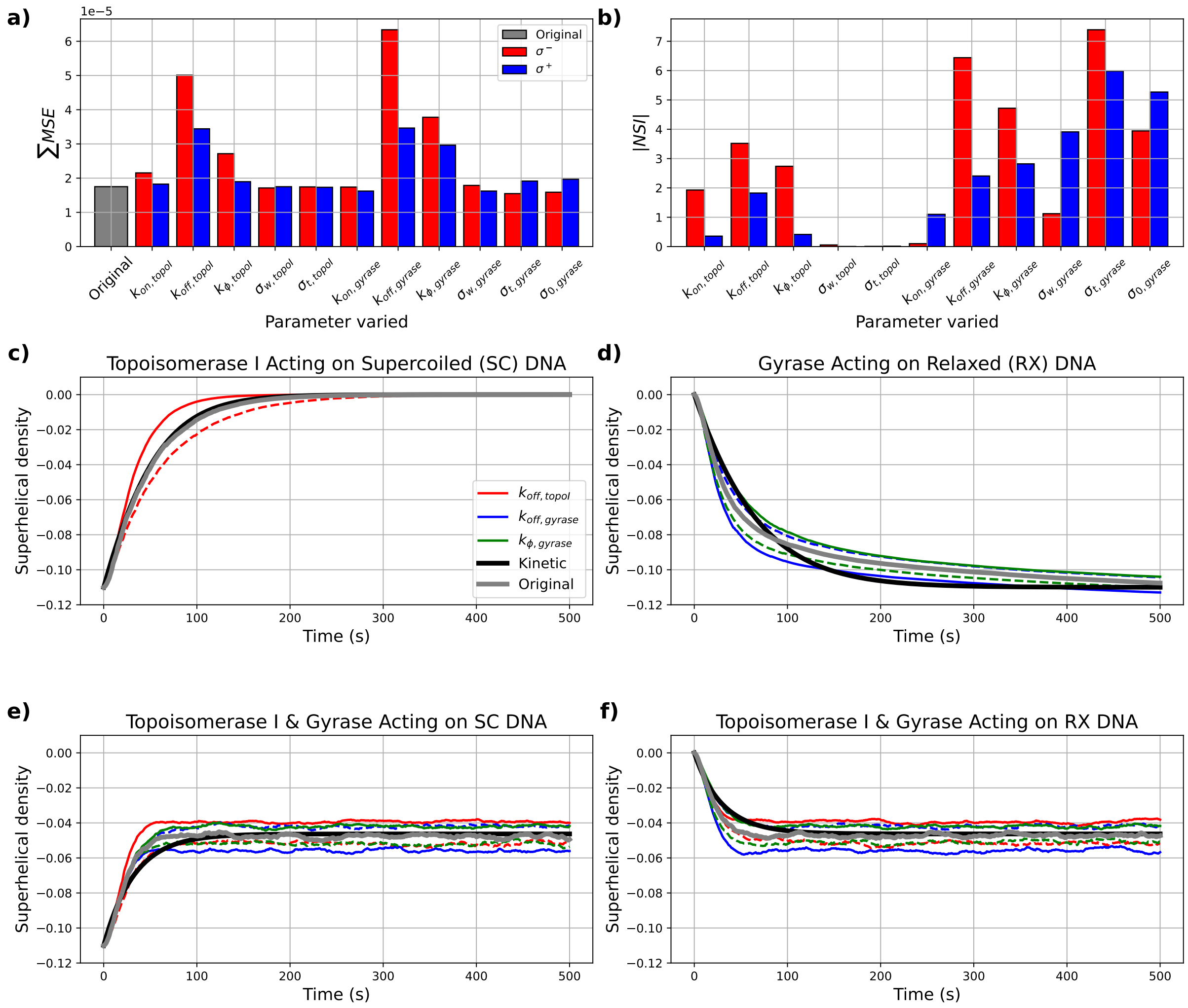

Figure S16: One-at-a-time sensitivity analysis of the stochastic topoisomerase model on supercoiled DNA, shown in terms of the sum of squared errors ($\sum MSE$) (a) and the normalised sensitivity index (NSI) (b). Simulation results for key parameter variations are shown for: topoisomerase I acting on supercoiled DNA (c), gyrase acting on relaxed DNA (d), both topoisomerases acting on supercoiled DNA (e), and both acting on relaxed DNA (f). The original (average) parameterisation is shown in grey, the kinetic model from experiments in black, variations in $k_{\mathrm{off,topoI}}$ in red, $k_{\mathrm{off,gyrase}}$ in blue, and $k_{\phi,\mathrm{gyrase}}$ in green. Solid lines indicate parameters decreased by one standard deviation (σ^{-}), and dashed lines indicate parameters increased by one standard deviation (σ^{+}).

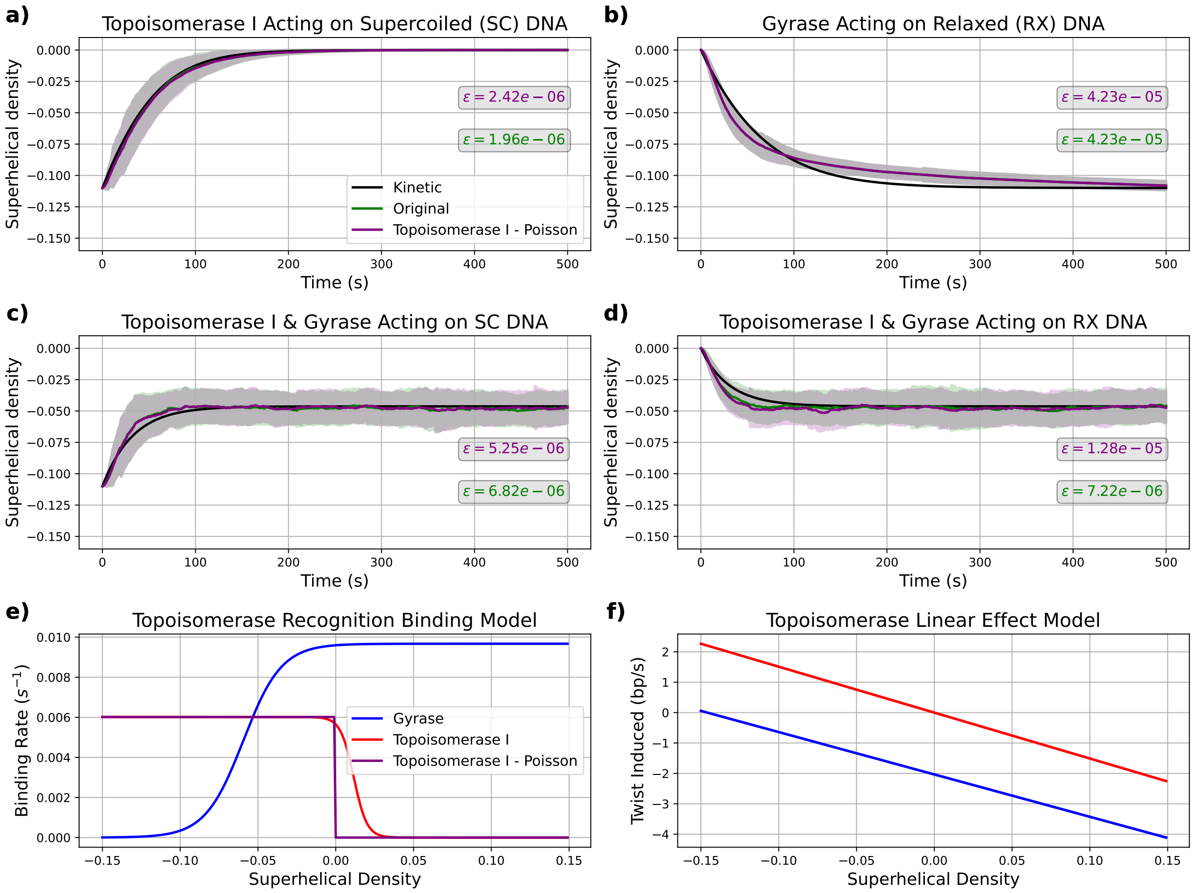

Figure S17: Stochastic topoisomerase activity parameterised from experimental data [11]. Simulated superhelical densities (a–d) are shown for TORCphysics topoisomerase models using the best-fit parameter set (green) and a simplified model with a constant topoisomerase I binding rate (purple), compared with superhelical densities inferred from experimental kinetic data (black). Results are shown for four conditions: (a) topoisomerase I acting on supercoiled DNA, (b) gyrase acting on relaxed DNA, (c) both topoisomerases acting on supercoiled DNA, and (d) both acting on relaxed DNA. Shaded areas represent standard deviations. The corresponding binding models are illustrated in (e), where the model with constant topoisomerase I binding is represented as a Poisson process active only on negatively supercoiled DNA. The effect models used in these simulations are shown in panel (f).

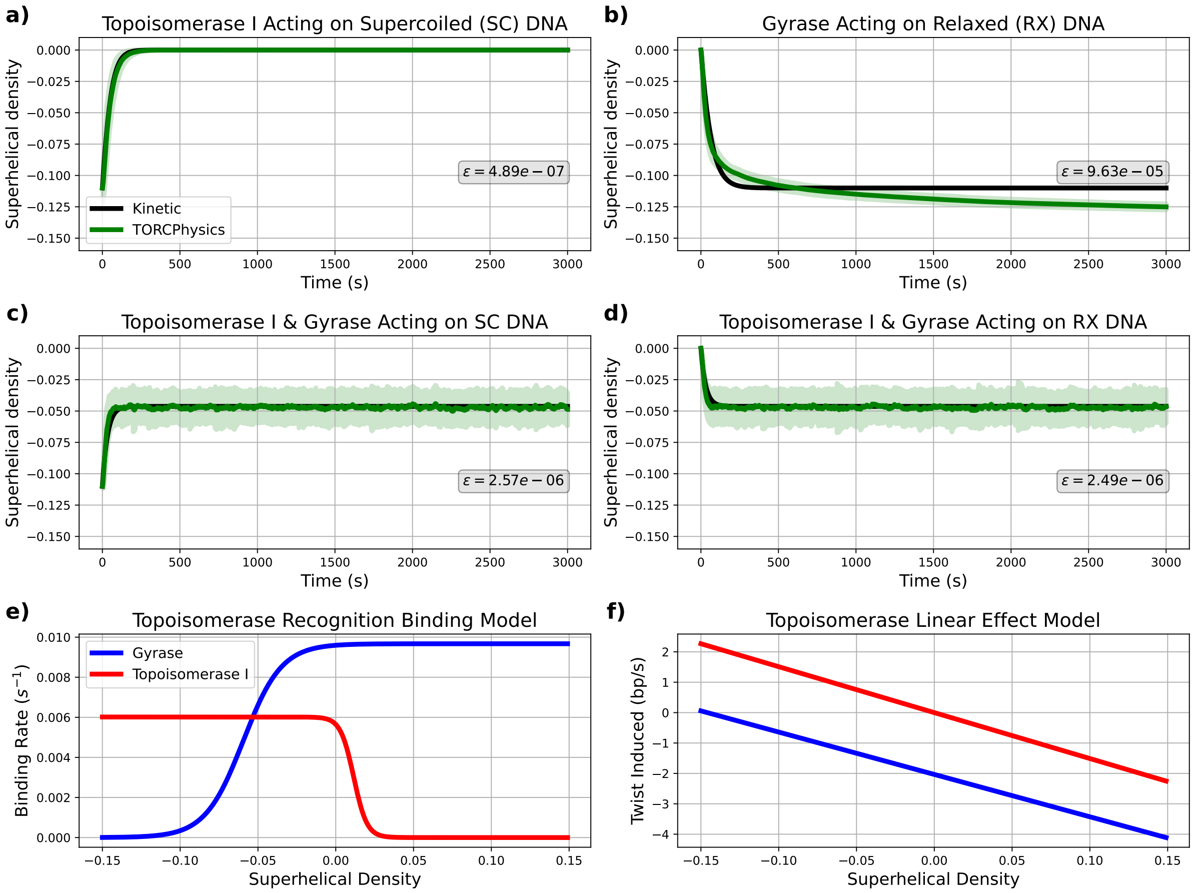

Figure S18: Extended stochastic topoisomerase activity parameterised from experimental data [11]. Simulated superhelical densities (a–d) are shown for TORCphysics topoisomerase models using the best-fit parameter set (green), compared with superhelical densities inferred from experimental kinetic data (black). Results are presented for four conditions: (a) topoisomerase I acting on supercoiled DNA, (b) gyrase acting on relaxed DNA, (c) both topoisomerases acting on supercoiled DNA, and (d) both acting on relaxed DNA. Simulations were extended to six times the duration of the original experiment, reaching 3000 seconds. Shaded areas represent standard deviations. The corresponding binding models are shown in (e) and the effect models in (f).

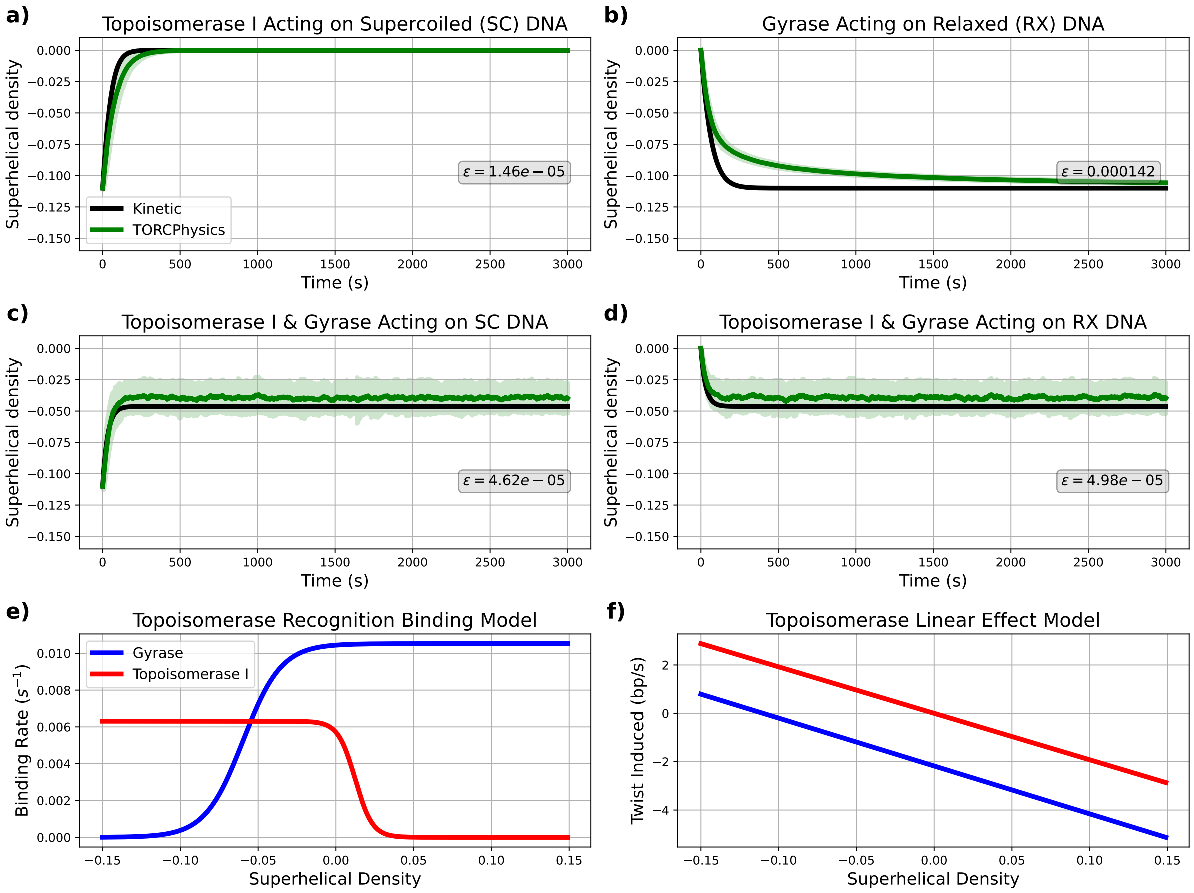

Figure S19: Extended stochastic topoisomerase activity parameterised from experimental data [11]. Simulated superhelical densities (a–d) are shown for TORCphysics topoisomerase models with the fixed parameter $\alpha_0 = -0.11$ using the best-fit parameter set (green), compared with superhelical densities inferred from experimental kinetic data (black). Results are presented for four conditions: (a) topoisomerase I acting on supercoiled DNA, (b) gyrase acting on relaxed DNA, (c) both topoisomerases acting on supercoiled DNA, and (d) both acting on relaxed DNA. For these simulations, a separate calibration process was performed to best fit the kinetic data. Shaded areas represent standard deviations. The corresponding binding models are shown in (e), and the effect models in (f).

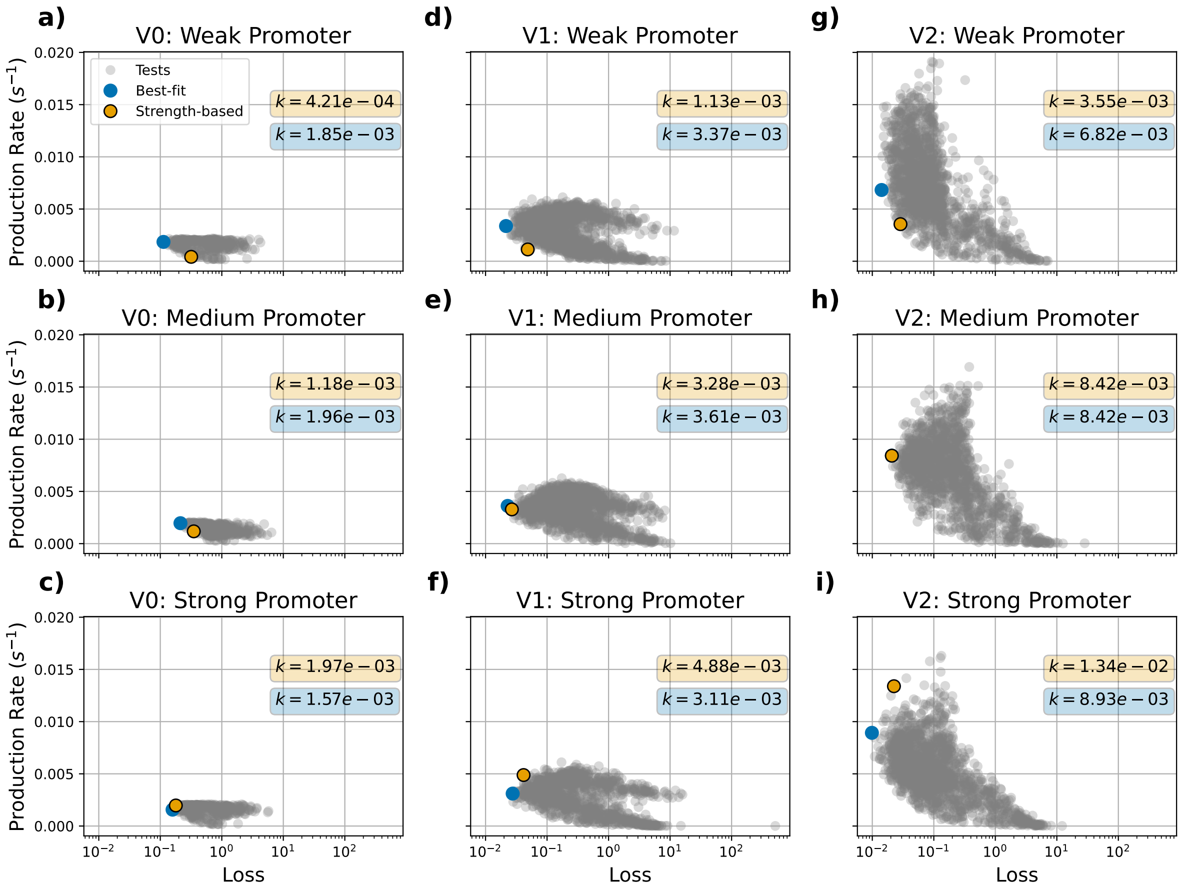

Figure S20: Averaged production rates from all parameterisation sets tested during the calibration process (gray) for the three model sets: V0 (a–c), V1 (d–f), and V2 (g–i). Parameterisations that best reproduce the experimental susceptibility with the lowest error are shown in blue, while those that also reproduce the relative expression rates observed in the experimental data from Boulas et al. [1] are shown in yellow (strength-based solutions). The corresponding averaged production rates (k) are indicated in the coloured boxes of each panel. In each test, transcription rates of the one-step model (V0) and the three-step models (V1 and V2) were varied, while the sequence-dependent sigmoid functions describing open-complex formation were pre-fitted using the SIST algorithm [2]. The RNAP twist-injection ratio (γ) was set to 0.05 in V0, 0.1 in V1, and 0.157 in V2.

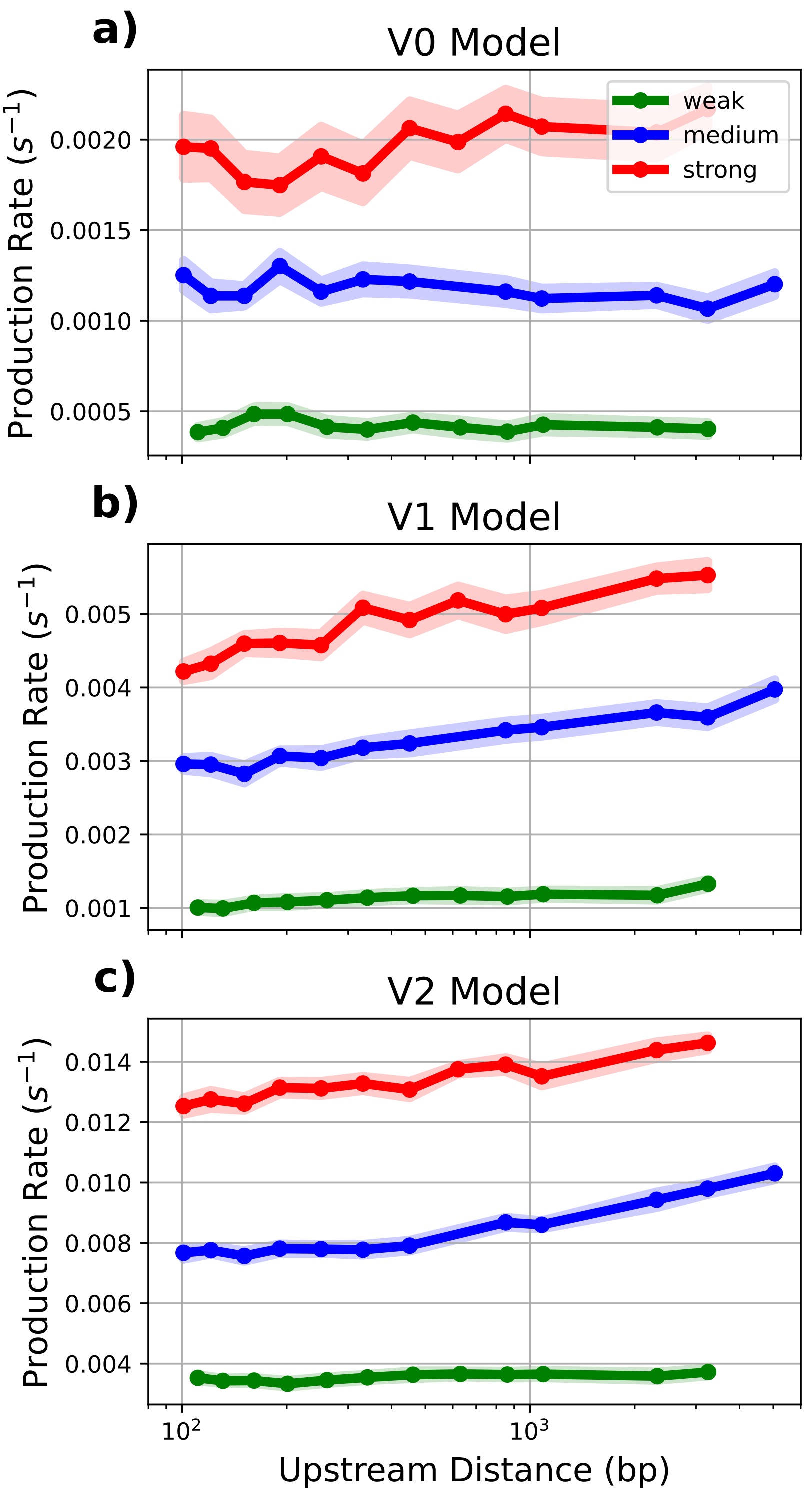

Figure S21: Averaged production rates as a function of the upstream barrier distance for the weak (green), medium (blue), and strong (red) promoters in the V0 (a), V1 (b), and V2 (c) models. The parameterisations were selected to best reproduce the susceptibility and the relative expression rates observed in the experimental data from Boulas et al. [1].

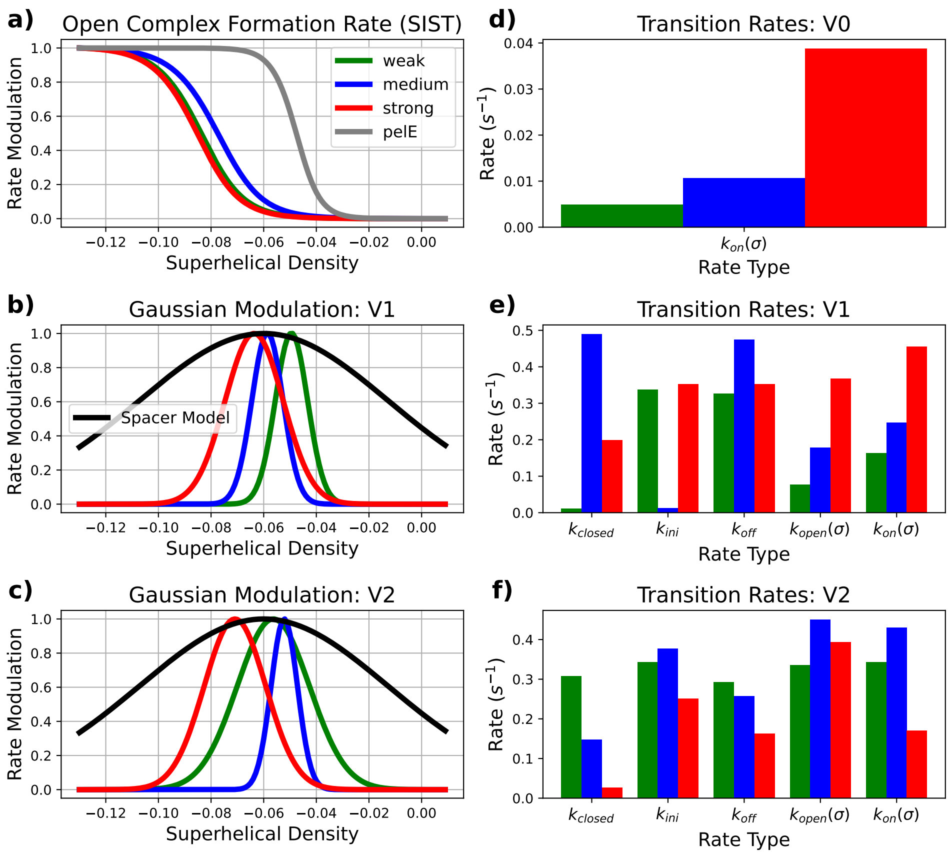

Figure S22: a) Open-complex formation rate modulation for the pelE promoter, calibrated in El Houdaigui et al. [18], alongside the modulation profiles of weak, medium, and strong promoters calibrated using the SIST algorithm [19] and sequences obtained from Boulas et al. [1]. b) and c) Closed-complex formation modulation for the weak, medium, and strong promoters in models V1 and V2, respectively, with the spacer length model from Forquet et al. [6] included for comparison. d) Binding rates k_{on} for model V0, promoter kinetic rates for models V1 e) and V2 f), resulted from the gene architecture calibration process. Both k_{on} and k_{open} rates are superhelical density dependent (σ). In V0, k_{on} is modulated by the sigmoidal function shown in (a), whereas in V1 and V2, k_{open} (e-f) is modulated by the same sigmoidal function. In V1 and V2, k_{on} is modulated by the Gaussian functions shown in (b-c).

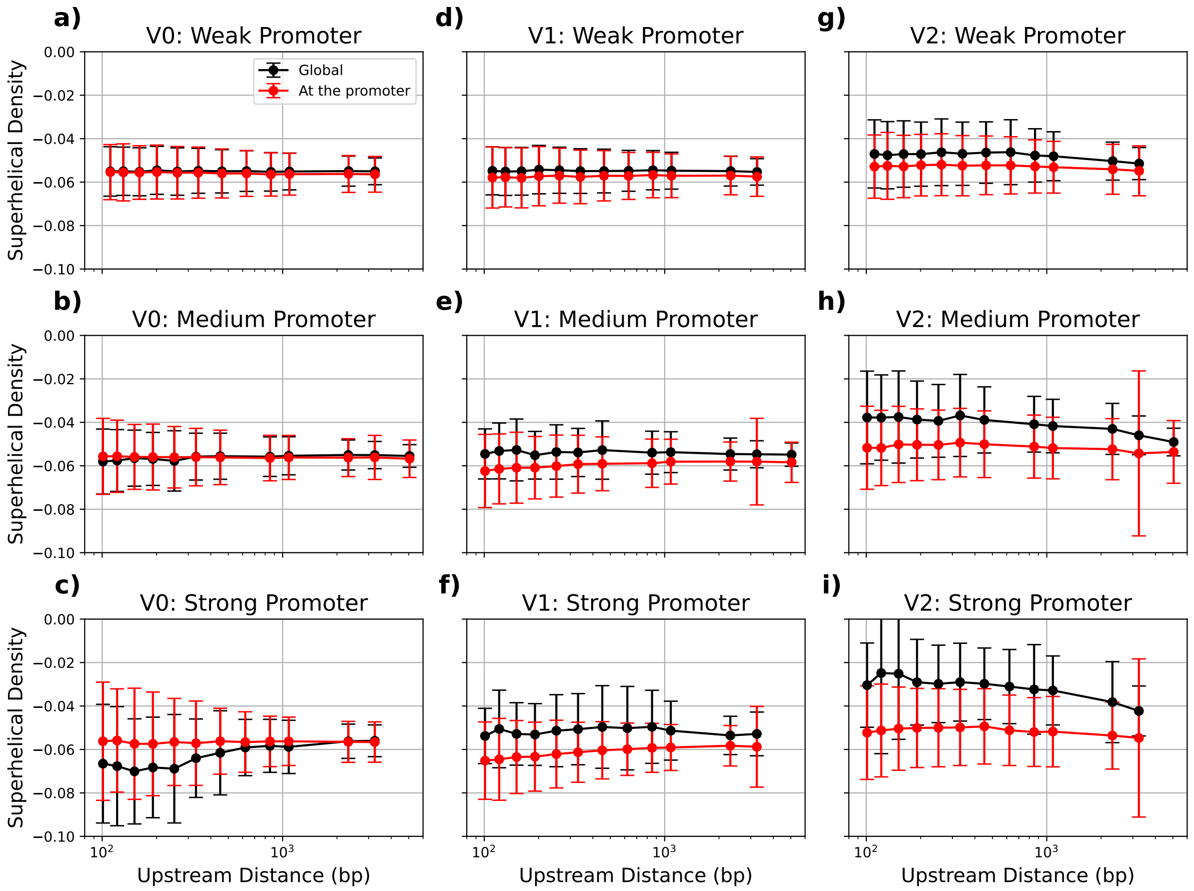

Figure S23: Global (black) and local (red, and at the promoter) superhelical densities as a function of the upstream barrier distance for the weak, medium, and strong promoters in the V0 (a–c), V1 (d–f), and V2 (g–i) models. The results correspond to parameterisations selected to reproduce both the susceptibility and the relative expression rates observed in the experimental data from Boulas et al. [1] (strength-based solutions). Data points represent averaged values, and error bars indicate standard deviations.

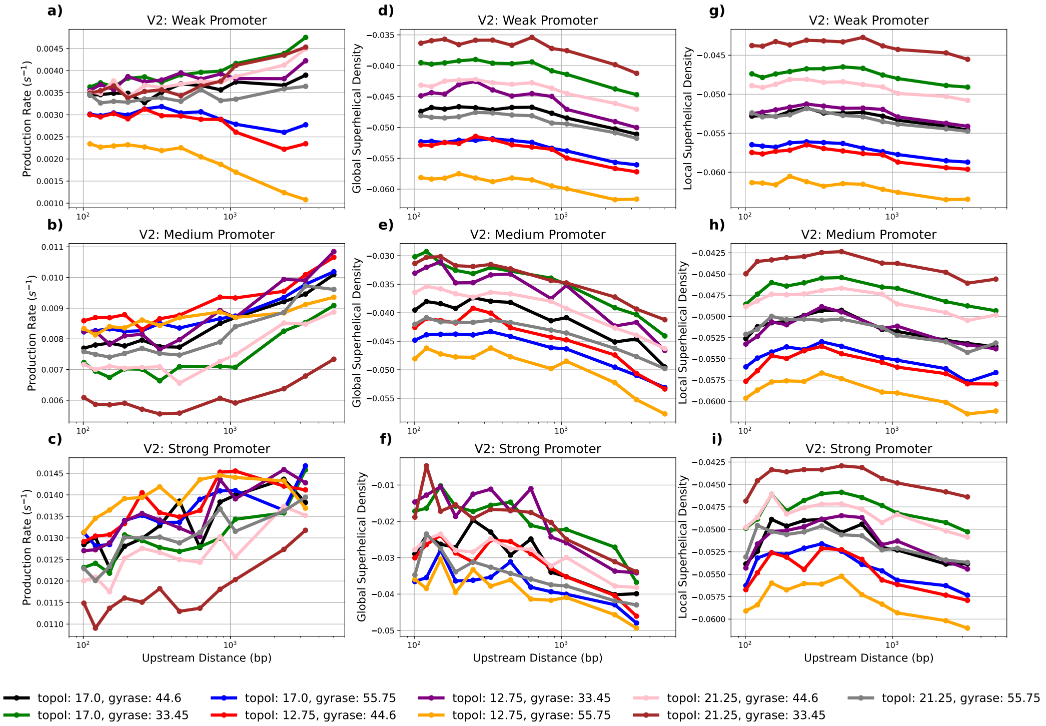

Figure S24: Averaged production rates (a–c), global (d–f) and local (g–i) superhelical density from simulations in the genetic architecture experiment for the V2 model, using promoter paramaterisations selected to reproduce the relative expression rates from the experimental data of Boulas et al. [1]. Results are shown for the weak, medium, and strong promoters using the parameterisations selected to reproduce the experimental relative expression rates, at varying topoisomerase concentrations. Topoisomerase levels were altered by ±25% relative to the baseline values used throughout the study. The base concentration of topoisomerase I is 17.0 nM, while gyrase is 44.6 nM (black line).

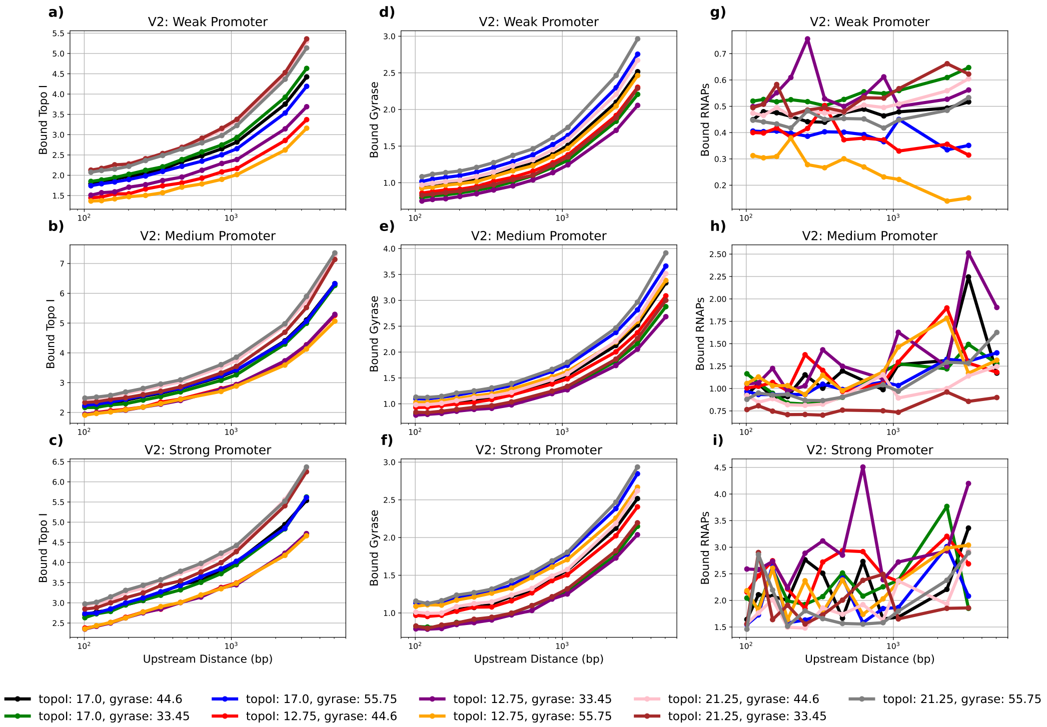

Figure S25: Averaged number of bound topoisomerase I (a–c), gyrase (d–f) and RNAP (g–i) from simulations in the genetic architecture experiment for the V2 model. Results are shown for the weak, medium, and strong promoters using the parameterisations selected to reproduce the experimental relative expression rates, at varying topoisomerase concentrations. Topoisomerase levels were altered by ±25% relative to the baseline values used throughout the study. The base concentration of topoisomerase I is 17.0 nM, while gyrase is 44.6 nM (black line).

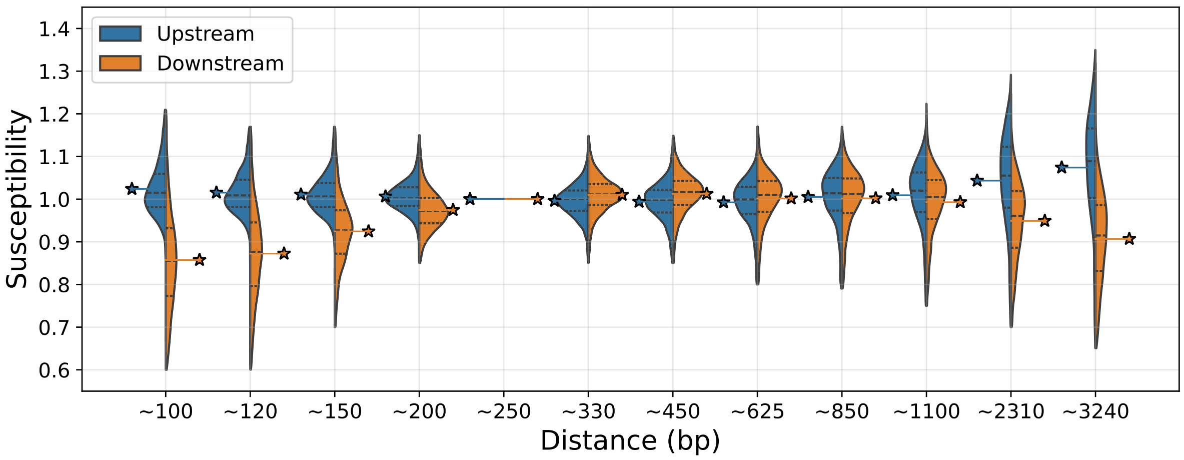

Figure S26: Distributions of gene expression susceptibilities as a function of upstream (blue) and downstream (orange) barrier distances in the V2 model. A total of 3,900 random parameterisation sets were tested. The reference distance used to calculate the susceptibility was of approximately 250 bp. The same parameter sets were used for both upstream and downstream barrier variations. Stars highlight the mean value of each distribution.

10 Supplementary Tables

Table S1: Values of parameters used in TORCphysics for calculating torques according to Marko's elastic model of supercoiled DNA [5]. The energies g and c_s are expressed in terms of free energy per unit length (i.e., units of force). The parameters p, c, g, c_s, σ_s, and σ_p were computed using the equations provided in Section 5, based on DNA stiffness and structural parameters, as well as temperature.

**Parameter**	**Value**	**Description**
f **(pN)**	1.00	Stretching forces
τ_0 **(pN nm)**	12.0	RNAP stalling torque
κ (**pN**$^{-1}$**nm**$^{-1}$)	0.50	Torque scaling factor
ω_0' **(rad/nm)**	1.76	Contour-length rate of rotation
T **(K)**	300	Temperature
A **(nm)**	50	Bending persistence length
P **(nm)**	24.0	Twist persisetence length of plectonomic DNA
p **(pN)**	304	Twist stiffness of plectonomic DNA
C **(nm)**	95.0	Twist persistence length
c **(pN)**	1206	Twist stiffness
g **(pN)**	.714	Free energy of stretched DNA
c_s **(pN)**	1042	Free energy of twisted DNA
σ_s	0.0238	Twist threshold
σ_p	0.0813	Writhe threshold

Table S2: Steady-State Kinetic Parameters of *E. coli* DNA Topoisomerase I (topo I) and DNA Gyrase, taken from the kinetic study conducted by Wang et al. (2019) [11].

Parameter	Topoisomerase I	Gyrase
k_{cat} (s^{-1})	2.3 x 10^{-3}	1.1 x 10^{-3}
K_M (nM)	1.5	2.7
$v_{\max}$ (pM/s)	40	50
E (nM)	17	45

Table S3: Promoter sequences used in this study for the gene architecture sections, taken from Boulas et al. [1]. The -10 promoter regions are highlighted in bold letters.

Name	Sequence
Weak	AAAAAGAGTATTGACTTCGCATCTTTTTGTACC**TATAAT**GTGTGGATAGCGG
Medium	TTGACATCAGGAAAATTTTTCTG**CATAAT**TATTTCATATCAC
Strong	TTGACATCGCATCTTTTTGTACC**TATAAT**GTGTGGATAGAGT

References

[1] Boulas, I., Bruno, L., Rimsky, S., Espeli, O., Junier, I., and Rivoire, O. (2023) Assessing in vivo the impact of gene context on transcription through DNA supercoiling. *Nucleic Acids Res.,* **51**(18), 9509–9521.

[2] Zhabinskaya, D., Madden, S., and Benham, C. J. (2014) SIST: stress-induced structural transitions in superhelical DNA. *Bioinformatics,* **31**(3), 421–422.

[3] Brochu, J., Vlachos-Breton, E., Irsenco, D., and Drolet, M. (2023) Characterization of a pathway of genomic instability induced by R-loops and its regulation by topoisomerases in E. coli. *PLoS Genet.,* **19**(5), 1–33.

[4] Du, X., Wojtowicz, D., Bowers, A. A., Levens, D., Benham, C. J., and Przytycka, T. M. (2013) The genome-wide distribution of non-B DNA motifs is shaped by operon structure and suggests the transcriptional importance of non-B DNA structures in Escherichia coli. *Nucleic Acids Res.,* **41**(12), 5965–5977.

[5] Marko, J. F. (2007) Torque and dynamics of linking number relaxation in stretched supercoiled DNA. *Phys. Rev. E,* **76**, 021926.

[6] Forquet, R., Nasser, W., Reverchon, S., and Meyer, S. (2022) Quantitative contribution of the spacer length in the supercoiling-sensitivity of bacterial promoters. *Nucleic Acids Res.,* **50**(13), 7287–7297.

[7] Velasco-Berrelleza, V., Burman, M., Shepherd, J. W., Leake, M. C., Golestanian, R., and Noy, A. (2020) SerraNA: a program to determine nucleic acids elasticity from simulation data. *Phys. Chem. Chem. Phys.,* **22**(34), 19254–19266.

[8] Skoruppa, E., Laleman, M., Nomidis, S. K., and Carlon, E. (2017) DNA elasticity from coarse-grained simulations: The effect of groove asymmetry. *J. Chem. Phys.,* **146**(21), 214902.

[9] Sharma, R., Patelli, A. S., De Bruin, L., and Maddocks, J. H. (2023) cgNA+web : A Visual Interface to the cgNA+ Sequence-dependent Statistical Mechanics Model of Double-stranded Nucleic Acids. *J. Mol. Biol.,* **435**(14), 167978 Computation Resources for Molecular Biology.

[10] Slavníková, P., Cuker, M., Matoušková, E., Čmelo, I., Zgarbová, M., Jurečka, P., and Lankaš, F. (2025) Sequence-Dependent Shape and Stiffness of DNA and RNA Double Helices: Hexanucleotide Scale and Beyond. *J. Chem. Inf. Model.,* **65**(17), 9208–9229 PMID: 40854823.

[11] Wang, Y., Rakela, S., Chambers, J. W., Hua, Z.-C., Muller, M. T., Nitiss, J. L., Tse-Dinh, Y.-C., and Leng, F. (2019) Kinetic study of DNA topoisomerases by supercoiling-dependent fluorescence quenching. *ACS omega,* **4**(19), 18413–18422.

[12] Cozzarelli, N. R. (1980) DNA gyrase and the supercoiling of DNA. *Science,* **207**(4434), 953–960.

[13] Higgins, N. P. and Vologodskii, A. V. (2015) Topological behavior of plasmid DNA. *Microbiol. Spectrum,* **3**(2), 10–1128.

[14] Liu, L. F. and Wang, J. C. (1987) Supercoiling of the DNA template during transcription. *Proc. Natl. Acad. Sci. U. S. A.,* **84**(20), 7024–7027.

[15] Sutormin, D., Galivondzhyan, A., Musharova, O., Travin, D., Rusanova, A., Obraztsova, K., Borukhov, S., and Severinov, K. (2022) Interaction between transcribing RNA polymerase and topoisomerase I prevents R-loop formation in E. coli. *Nat. Commun.,* **13**(1), 4524.

[16] Boles, T. C., White, J. H., and Cozzarelli, N. R. (1990) Structure of plectonemically supercoiled DNA. *J. Mol. Biol.,* **213**(4), 931–951.

[17] Adrian, M., ten Heggeler-Bordier, B., Wahli, W., Stasiak, A. Z., Stasiak, A., and Dubochet, J. (1990) Direct visualization of supercoiled DNA molecules in solution.. *EMBO J.,* **9**(13), 4551–4554.

[18] El Houdaigui, B., Forquet, R., Hindré, T., Schneider, D., Nasser, W., Reverchon, S., and Meyer, S. (2019) Bacterial genome architecture shapes global transcriptional regulation by DNA supercoiling. *Nucleic Acids Res.,* **47**(11), 5648–5657.

[19] Wang, H., Noordewier, M., and Benham, C. J. (2004) Stress-induced DNA duplex destabilization (SIDD) in the E. coli genome: SIDD sites are closely associated with promoters. *Genome Res.,* **14**(8), 1575–1584.
